# Host *tp53* mutation induces gut dysbiosis eliciting inflammation through disturbed sialic acid metabolism

**DOI:** 10.1186/s40168-021-01191-x

**Published:** 2022-01-06

**Authors:** Jae-Geun Lee, Soohyun Lee, Juhee Jeon, Hyun Gi Kong, Hyun-Ju Cho, Jong-Hwan Kim, Seon-Young Kim, Myung Jin Oh, Daum Lee, Nari Seo, Ki Hun Park, Kweon Yu, Hyun Joo An, Choong-Min Ryu, Jeong-Soo Lee

**Affiliations:** 1 Disease Target Structure Research Center, KRIBB, Daejeon, 34141 Republic of Korea; 2 KRIBB School, University of Science and Technology, 217 Gajeong-ro, Yuseong-gu, Daejeon, 34113 Republic of Korea; 3 Infectious Disease Research Center, KRIBB, Daejeon, 34141 Republic of Korea; 4 Stembio. Ltd, Entrepreneur 306, Soonchunhyang-ro 22, Sinchang-myeon, Asan-si, Chungcheongnam-do 31538 Republic of Korea; 5 Crop Protection Division, National Institute of Agricultural Sciences, Rural Development Administration, Wanju-gun, Jeollabuk-do 54875 Republic of Korea; 6 Dementia DTC R&D Convergence Program, KIST, Hwarang-ro 14 gil 5, Seongbuk-gu, Seoul, 02792 Republic of Korea; 7 Korean Bioinformation Center, KRIBB, Daejeon, 34141 Republic of Korea; 8 Graduate School of Analytical Science and Technology, Chungnam National University, Daejeon, 34134 Republic of Korea; 9 Division of Applied Life Science (BK21 plus), IALS, Gyeongsang National University, Jinju-si, Gyeongsangnam-do 52828 Republic of Korea

**Keywords:** Zebrafish, Larval intestine, Host, *tp53* mutation, Inflammation, Microbiota, Dysbiosis, Sialometabolism, Germfree, Sialidase inhibition

## Abstract

**Background:**

Host *tp53* mutations are frequently found during the early stages of colitis-associated colorectal cancer (CAC), but whether such mutations induce gut microbiota dysbiosis and chronic intestinal inflammation that contributes to the development of CAC, remains unknown.

**Results:**

We found that zebrafish *tp53* mutant larvae exhibited elevated intestinal inflammation, by monitoring the *NFκB* activity in the mid-distal intestines of zebrafish larvae using an *NFκB:EGFP* transgenic reporter line in vivo as well as neutrophil infiltration into the intestine. This inflammation was due to dysbiotic gut microbiota with reduced diversity, revealed using both 16S rRNA amplicon sequencing and a germfree larva model. In this dysbiosis, *Aeromonas* spp. were aberrantly enriched as major pathobionts and exhibited the capacity for aggressive colonization in *tp53* mutants. Importantly, the ex-germfree experiments supported the causality of the host *tp53* mutation for inducing the inflammation. Transcriptome and high-performance liquid chromatography analyses of the host gastrointestinal tracts identified dysregulated sialic acid (SA) metabolism concomitant with increased host Neu5Gc levels as the key determinant of aberrant inflammation, which was reversed by the sialidase inhibitors oseltamivir and Philippin A.

**Conclusions:**

These results demonstrate a crucial role for host *tp53* in maintaining symbiosis and immune homeostasis via SA metabolism. Disturbed SA metabolism via a *tp53* mutation may be exploited by specific elements of the gut microbiome, eliciting both dysbiosis and inflammation. Manipulating sialometabolism may therefore provide an efficacious therapeutic strategy for *tp53* mutation-induced dysbiosis, inflammation, and ultimately, related cancers.

**Video Abstract**

**Supplementary Information:**

The online version contains supplementary material available at 10.1186/s40168-021-01191-x.

## Highlights

- Zebrafish *tp53*-mutant larvae exhibit gut dysbiosis responsible for elevated inflammation
- The host *tp53* mutation dysregulates sialic acid metabolism supporting the overgrowth of pathobionts
- Normalizing the sialic acid metabolism imbalance can be a therapeutic strategy for intestinal dysbiosis and inflammation

## Background

Chronic inflammation, a steady, low-level of inflammation caused by autoimmune responses, infections, metabolic dysfunction, and environmental contaminants, predisposes humans to several types of cancer in different organ systems [1–3]. In particular, chronic inflammation of the colon (colitis) has been well established to accompany intestinal dysplasia and to eventually promote colorectal cancer (CRC) in colitis-associated colorectal cancer (CAC) [4, 5]. This process is exemplified by inflammatory bowel disease (IBD), where prolonged gut inflammation has been shown to increase the risk of CRC by two- to six-fold [6–8]. Chronic inflammation-mediated CRC is known to be promoted by alterations in the microenvironment that support tumorigenesis via increased cytokine/chemokine expression, aberrant immune cell recruitment, and elevated reactive oxygen species (ROS) that can damage both DNA and proteins [3]. During this progression, nuclear factor kappa B (NF-κB) signaling in both epithelial and immune cells plays a key role in connecting chronic inflammation to cancer development [9].

A series of genetic analyses have identified several mutations in oncogenes and in tumor suppressor genes (e.g., *APC*, *K-RAS*, and *TP53*) during CRC pathogenesis. Among these, mutations of the *TP53* tumor suppressor, or its loss, appear distinctly crucial to CAC pathogenesis. In contrast to sporadic CRC cases, they are frequently found at early stages of tumorigenesis, even before obvious CAC dysplasia is evident [10–12]. Recent studies have demonstrated that such early *TP53* mutations during CAC progression contribute to the induction of NF-κB-dependent chronic inflammation [13] and compromise the integrity of intestinal epithelial cells associated with the inflammatory microenvironment [14], supporting the idea that *TP53* mutations are critical host CAC initiation factors. In addition, augmentations in NF-κB signaling shown by *TP53* gain-of-function experiments have been well established [15, 16].

Another important factor implicated in both intestinal inflammation and CRC is the commensal microbiota that colonizes the intestine [17]. The commensal microbiota is known to play an essential role not only in the development, metabolism, and immunity of the host under physiological conditions but is also involved in a variety of inflammatory, metabolic, and neurological diseases when microbiota symbiosis is disrupted, known as microbiota dysbiosis [18–22]. In particular, such intestinal dysbiosis has been strongly implicated in the pathogenesis of IBD in both human and mouse models [23–25] and in CRC, including CAC [26–32]. For example, a decrease in the alpha-diversity (richness and evenness), represented by a reduction of *Bacteroidetes* and increased abundance of *Proteobacteria*, has been observed in IBD [33, 34]. Such dysbiosis may promote CRC either indirectly by eliciting altered pro-inflammatory responses in the host microenvironment in models of IBD, where elevated inflammation was blocked or reduced under germfree (GF) or *Tlr*/*Myd88*-deficient conditions [19, 35], or directly through the production of genotoxic/regulatory metabolites of pathogenic bacteria, such as *pks+ Escherichia coli*-derived colibactin, which alkylated DNA in a *Il10*-deficient mouse model [36, 37]. Correcting such dysbiosis may therefore have therapeutic value for treating CAC, as in experimental CAC mouse models where inhibition (using tungsten salt) of the pro-tumoral *Enterobacteriaceae* family reduced both inflammation and tumor incidence [38].

One common denominator that may affect both host *tp53* mutation- and dysbiosis-mediated inflammation/cancer is sialic acid (SA) metabolism. SA, a nine-carbon carbohydrate with a variety of modifications, has been implicated not only in physiological regulation during development but also in pathological conditions, including many cancer types. SAs often aberrantly coat cancer cells as the terminal sugars of upregulated cancer-associated sialylated glycans, such as Sialyl-Thomsen-nouveau (STn) and Sialyl Lewis-X, contributing to the survival, metastases, and immune responses of host cancer cells, while also serving as cancer biomarkers [39, 40]. At the same time, SA derivatives located at the terminal positions of host glycans in the intestinal mucosa are carbon, nitrogen, and energy sources for commensal/pathogenic bacteria to support their growth via sialocatabolic pathways or are used as cell-wall precursors to mask the bacteria in order to evade host immune-surveillance [41, 42]. Indeed, the importance of proper SA metabolism for maintaining the microbiota has been shown in β-galactoside α-2,3-sialyltransferase *St3gal4* knockout mice that displayed dysbiosis (i.e., reduced *Ruminococcaceae* and enriched *Porphyromonadaceae*) compared to wild-type mice as adults [43]. The contributions of *TP53* mutations to CAC pathogenesis is often attributed to susceptible DNA damage due to ROS production [11], NF-κB signaling dysregulation [16], or defective junctions [14] in affected intestinal cells in a cell-autonomous manner. *TP53* mutations have been also implicated in glycan metabolism, where expression of STn and Tp53 proteins was correlative in a bladder-cancer mouse model [44] and *N*-glycosylated protein stability was regulated via a UDPase (ENTPD5) as a mutant Tp53 target [45]. Despite the potential involvement of SA metabolism for mediating interactions between host genetics and the microbiome via non-cell autonomous roles for *TP53* mutations to regulate intestinal dysbiosis and inflammation, the pathological context of this intricate relationship has not been investigated in vivo in detail. We therefore hypothesized that a sialylated microenvironment containing *tp53*-mutation-harboring, tumor-prone cells would promote an abundance of SA-utilizing dysbiotic pathobionts and elicit a pro-inflammatory response that would ultimately lead to cancer development. A better understanding of the relationship between *tp53* mutations and dysbiosis with respect to SA metabolism could provide a preemptive therapeutic approach based on microbial manipulation to convert dysbiosis to eubiosis through the use of prebiotics, probiotics, antibiotics, and bacteriophages [46–49].

To study the relationship between *TP53* mutations and dysbiosis via SA metabolism, we adopted a zebrafish larval model that represents an important animal model for mechanistic and translational studies [50]. This model is genetically tractable as it utilizes knockout and transgenic animals [51]; is optically transparent, allowing high-resolution in vivo imaging [52]; and is anatomically and functionally similar to corresponding human organs, including the intestine [53, 54]. Most importantly, it is amenable to metagenomic analyses of the intestinal microbiota [55] in combination with established GF rearing conditions and ex-germfree (ex-GF) experimental settings for interrogating individual or collective functions of the microbiota [56].

Here, we demonstrate that aberrantly elevated inflammation in the intestines of zebrafish *tp53* mutant larvae is attributable to intestinal dysbiosis with enriched pathobionts. We demonstrate a dominant role for the host *tp53* mutation over dysbiotic microbiota in the inflammatory response using ex-GF experiments, and reveal aberrant sialometabolism in *tp53*-mutant intestines that is suppressed by sialidase-inhibitor treatments. These results will contribute to a rational therapeutic design whereby SA availability is limited using sialidase inhibition to treat both inflammatory diseases and colitis-associated cancer.

## Results

### Elevated immune responses in *tp53*-mutation larval zebrafish intestines are dependent on the intestinal microbiota

**Fig. 1:**
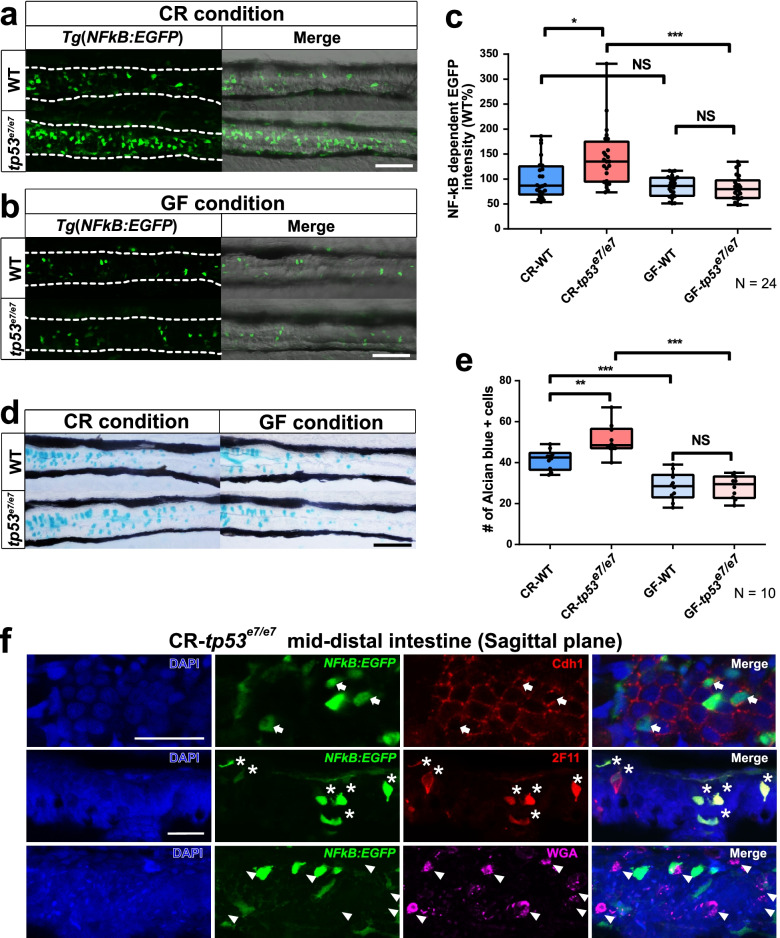
*tp53* mutants exhibit elevated NF-κB signaling in the mid-distal intestines due to intestinal bacteria. **a**, **b** NF-κB signaling in the mid-distal intestines of WT or *tp53* mutants was monitored using *Tg(NFκB:EGFP)* transgenic zebrafish larvae at 7 dpf. CR (**a**), conventionally raised. GF (**b**), germfree. Scale bar = 50 μm. White dashed lines denote boundaries of GITs based on DIC bright field images. **c** A boxed plot showing comparison of fluorescence intensity of the mid-distal intestines of WT and *tp53* mutants under CR- or GF-condition. *N* = 24 each. **d** Alcian blue staining visualizing goblet cells in the mid-distal intestines of WT or *tp53* mutants under CR- or GF- conditions. *N* = 10 each. Scale bar = 50 μm. **e** A boxed plot showing comparison of the number of Alcian blue-positive goblet cells in the mid-distal intestines of WT and *tp53* mutants under CR- or GF- conditions. **f** To reveal cell types of NF-κB positive cells, EGFP-positive cells in the sagittal sections through the mid-distal intestine of *tp53* mutant *Tg*(*NFκB:EGFP*) at 7dpf under CR condition stained for markers of pan-secretory cells (2F11), enterocytes (Cdh1), goblet cells (WGA) using immunohistochemistry. The NF-κB dependent EGFP-positive intestinal cells were clearly positive with Cdh1 (white arrows) and 2F11 (white asterisks), but negative with WGA (white arrowheads)-positive cells. Scale bar = 20 μm. The boxed plots in (**c**) and (**e**) were statistically estimated by non-parametric Friedman test followed by Dunn’s multiple comparisons test. Data are represented as mean ± SEM. *< *p* = 0.05; **< *p* = 0.01; ***< *p* = 0.005

It has been shown that *TP53* mutation contributes to the increase in NF-κB-dependent chronic inflammation in both cell lines and mouse models [13]. By using the *Tg*(*NFκB:EGFP*) zebrafish transgenic line that allows for monitoring the signaling activity of NF-κB, a master regulator of inflammation [57, 58], we compared the 7-day post-fertilization (dpf) intestinal NF-κB activity of conventionally reared (CR) wild-type (WT) zebrafish to *tp53*^*e7/e7*^ mutants harboring a missense mutation in the *tp53* DNA binding domain [59]. Consistent with previous findings, NF-κB-dependent EGFP intensity in the mid-intestines of *tp53*-mutant larvae at 7 dpf was significantly upregulated compared to that of WT larvae (142 ± 11.8% increase; Fig. 1a, c). In order to address whether this elevated NF-κB signaling observed in *tp53* mutants was caused by the intestinal microbiota, the intestinal NF-κB signaling of WT and *tp53-*mutant larvae reared under GF conditions was examined. The increased NF-κB-EGFP signal in CR *tp53* mutants was abolished under GF conditions (Fig. 1b, c), indicating that the microbiota in CR *tp53* mutants was responsible for the increased inflammation. Concomitantly, the number of goblet cells stained by Alcian blue in the mid-distal intestines of *tp53*-mutant larvae at 7 dpf was increased compared to WT controls (123 ± 5.5% increase; Fig. 1d, e), and this increase was blocked under GF conditions (Fig. 1d, e), also revealing the dependency of this phenotype on the microbiota. Furthermore, increased neutrophil infiltration into the intestine, visualized using *Tg*(*mpx:mCherry*) transgenic zebrafish, was observed in response to this elevated inflammation in CR *tp53* mutants at 7 dpf [60] (Additional file 1: Fig. S1a, b). It should be noted that similar increases in NF-κB-dependent EGFP expression and in Alcian blue-positive goblet-cell numbers in *tp53* mutants were mimicked by dextran sulfate sodium (DSS) treatment, a well-known colitis-inducing chemical reagent (Additional file 1: Fig. S1 c-f). Taken together, the elevated NF-κB signaling, infiltration of neutrophil, and the response to DSS treatment observed in *tp53* mutants all indicate the increased inflammation in gastrointestinal tracts (GITs) of *tp53* mutants.

The schemes and timelines used for the present experiments are summarized in Fig. S1a. Despite these changes, there were no gross developmental or histological differences between WT and *tp53* mutants and their gastrointestinal tracts (GITs) using general-morphology analysis (Additional file 1: Fig. S2b) and H&E staining (Fig. S1c).

To identify the types of cells with elevated NF-κB-dependent EGFP in *tp53*-mutant mid-distal intestines, sagittal sections through that region were immunostained for enterocytes (anti-Cdh1), pan-secretory (goblet and enteroendocrine) cells (2F11), and goblet cells (WGA) [61, 62]: EGFP-positive intestinal cells were both anti-Cdh1- and 2F11-positive (Fig. 1f; arrows in the first row, and asterisks in the middle row, respectively) but WGA-negative (Fig. 1f; arrowhead in the bottom row), suggesting that NF-κB-EGFP-positive cells in the *tp53*-mutant intestine are most likely enterocytes and enteroendocrine cells.

### CR *tp53* mutants exhibit gut microbiota dysbiosis with a group of aberrantly enriched bacteria

To narrow the list of possible bacterial families responsible for the increased inflammation in *tp53* mutants, we used vancomycin and polymyxin B to selectively killed Gram-positive and Gram-negative bacteria, respectively, and documented the resulting changes in Alcian blue-positive goblet-cell numbers (Additional file 1: Fig. S3a). As a result, Gram-negative removal by polymyxin B blocked the increase in goblet-cell numbers, similar to the GF-condition numbers for the *tp53* mutants (Additional file 1: Fig. S3c) and the WT larvae numbers (Additional file 1: Fig. S3b), suggesting that the increased inflammation was primarily due to Gram-negative bacteria in the microbiota.

**Fig. 2:**
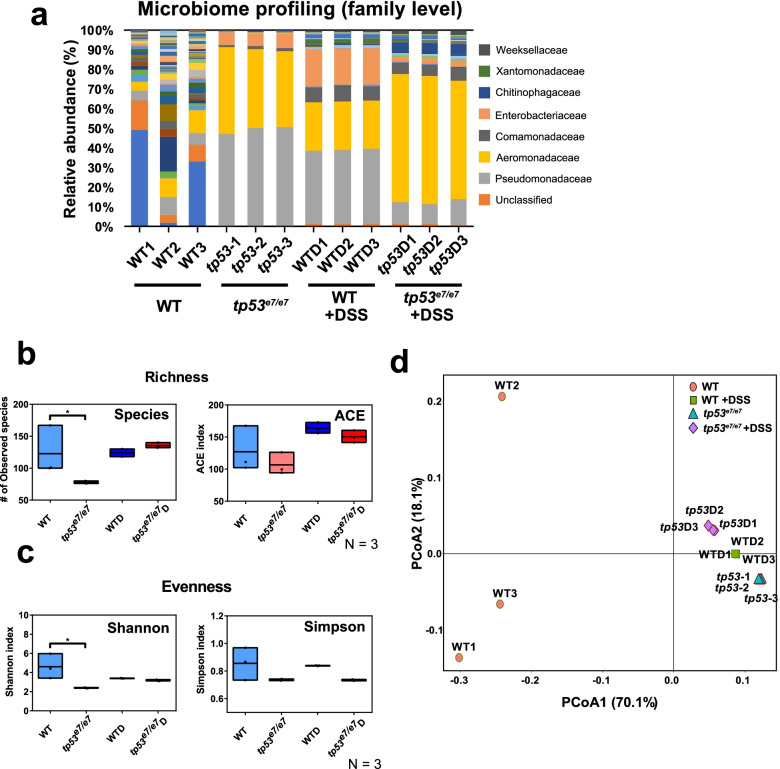
*tp53* mutants harbor intestinal dysbiosis revealed by metagenome analysis. **a** Metagenome analysis of the intestinal microbiota of WT and *tp53* mutants with or without DSS treatment at a family level. Each family is color-coded according to the key panel on the right. Each sample was repeated three times. **b**, **c** Alpha diversity (richness and evenness) comparison of the microbiota of WT, *tp53* mutants, WT+DSS (WTD), and *tp53* mutants + DSS (*tp53D*). **d** Principle coordinate analyses (PCoA, unweighted) visualizing the microbiotal relationship of samples from different conditions. PCoA scores were plotted based on the relative abundance of OTUs of taxonomic families of gut microbiota from WT and *tp53* mutants. Samples from the same conditions were grouped together except WT samples. The boxed plots in (**b**) and (**c**) were statistically estimated by one-way ANOVA followed by Newman-Keuls multiple comparisons test. Data are represented as mean ± SEM. *< *p* = 0.05

For direct evidence, a 16S rRNA amplicon sequencing analysis of GITs from zebrafish larvae at 7 dpf was performed in CR *tp53* mutants and compared to CR WTs (Fig. 2). The relative abundances of the bacterial populations within CR *tp53* mutants were analyzed at both the family (Fig. 2a) and class levels (Additional file 1: Fig. S4a). Consistent with the aforementioned results, the intestinal microbes in *tp53* mutants were much less diverse compared with the diversity seen in CR WTs. Bacteria diversity in the *tp53* mutants showed reduced richness (Fig. 2b) and evenness (Fig. 2c) compared with their WT counterparts, indicating dysbiosis of the intestinal microbiome in CR *tp53* mutants. The results of the principal coordinates analysis (PCoA) using weighted UniFrac for beta diversity of each genotype showed that CR WTs were grouped separately from CR *tp53* mutants (Fig. 2d). Interestingly, Gamma-proteobacteria, one of the main classes of Gram-negative pathogenic bacteria that is known to expand under inflamed conditions [63], were highly enriched in the *tp53* mutants, relative to Alpha-proteobacteria class (Additional file 1: Fig. S4a, 4d), whereas Bacilli and Actinobacteria did not exhibit significant differences in CR *tp53* mutants compared to the WT group (Additional file 1: Fig. S4b, 4c [64];). As controls, DSS-treated microbiomes from WT or *tp53* mutants were examined, and also displayed reduced diversities in their own PCoA profiles, suggesting distinct dysbiosis (Fig. 2 and Additional file 1: Fig. S4).

A detailed operational taxonomic unit (OTU) analysis of the bacteria enriched in *tp53* mutants based on 16S rRNA amplicon sequencing revealed that *Aeromonas*, *Pseudomonas*, and *Citrobacter* genera (all belonging to Gamma-proteobacteria) were the major bacterial groups aberrantly increased in CR *tp53* mutants (Additional file 1: Fig. S5). We also attempted to isolate culturable larval-gut bacteria to compare with the 16S rRNA gene analysis results. *Aeromonas jandaei*, *Pseudomonas otitidis*, and *Citrobacter freundii* were successfully cultivated under aerobic and anaerobic culture conditions from *tp53* mutant GITs and were classified as bacterial species aberrantly abundant in CR *tp53* mutants (refer to Methods for more details). In the following experiments, the *A*. *jandaei* strain, designated as *A*. *jandaei* TP531, was identified as a key representative pathobiont that selectively overpopulated the GITs of *tp53* mutants.

### Preferential *A. jandaei* colonization in *tp53* mutants elicits sensitized responses

**Fig. 3:**
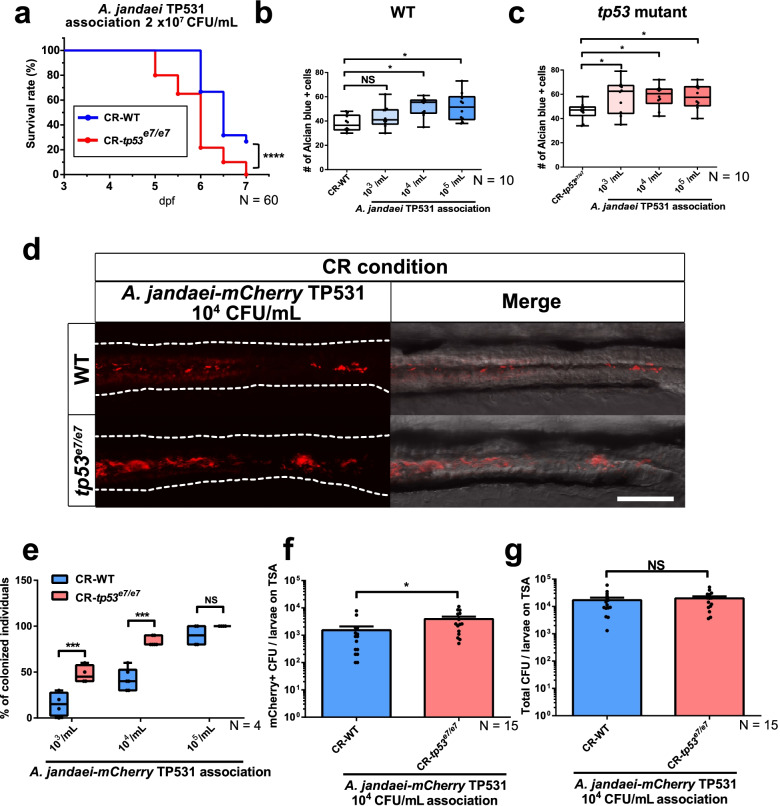
*Aeromonas jandaei* TP531 preferentially elicits increased goblet cell numbers and colonizes GITs of *tp53* mutants. **a** A lethality curve of *Aeromonas jandaei* TP531 association (2 × 10^7^ CFU/mL) with WT or *tp53* mutant larvae starting at 3 dpf. *N* = 60 each. The lethality curve was statistically estimated by Gehan-Breslow-Wilcoxon test. **b**, **c** Association of increasing titers of *A*. *jandaei* TP531 (10^3^~10^5^ CFU/mL) increased the number of Alcian blue-positive goblet cells in the mid-distal intestines of *tp53* mutants (**c**) more than of WT (**b**) at 7 dpf. *N* = 10 each. **d** Association of *mCherry*-tagged *A. jandaei* TP531 (10^4^ CFU/mL) preferred to colonize the GITs of *tp53* mutants under CR (conventionally raised) condition. White dashed lines denote boundaries of GITs based on DIC bright field images. Scale bar = 50 μm. **e** A boxed plot of percentages of WT or *tp53* mutants larvae colonized by *mCherry*-tagged *A*. *jandaei* TP531 at 7 dpf. *N* = 4 each. The boxed plots were statistically estimated by non-parametric Friedman test followed by Dunn’s multiple comparisons test. **f**, **g** Bar graph of the numbers of *mCherry*-tagged *A*. *jandaei* TP531 colonies (**f**) or total colonies (**g**) in TSA plates. *N* = 15 each. The bar graph was statistically estimated by Mann-Whitney *U* test. Data are represented as mean ± SEM. *< *p* = 0.05; ***< *p* = 0.005; ****< *p* = 0.0001. *NS* not significant

To test whether *A*. *jandaei* TP531 was a pathobiont responsible for the upregulated NF-κB signaling and increased goblet cell numbers observed in GITs of CR *tp53* mutants, lethality (bacterial pathogenicity) in zebrafish larvae was first tested by administering a high dose (2 × 10^7^ colony forming unit (CFU)/mL) of *A*. *jandaei* TP531. Under these conditions, the bacteria caused lethality in both genotypes, with *tp53* mutants being more susceptible to *A*. *jandaei* TP531 than WTs (*p* < 0.0001; Fig. 3a). In addition, exposure to increasing titers of *A*. *jandaei* TP531 resulted in increased goblet cell numbers in a bacteria concentration-dependent manner, with *tp53* mutant hosts being sensitized to the bacteria, and showing maximal responses at lower titers compared to WT hosts (Fig. 3b, c). As a control, exposures to nonpathogenic *E*. *coli* DH10B (1 × 10^8^ CFU/mL) did not induce lethality nor an increase in goblet cell numbers (*E*. *coli* 1 × 10^5^ CFU/mL) for either genotype (Additional file 1: Fig. S6). As another control, association of *Photobacterium damselae* DreWT1 (a commensal bacterial strain isolated from WT guts) neither increased lethality (1 × 10^8^ CFU/mL) nor increased NF-κB-dependent EGFP expression (1 × 10^5^ CFU/mL) for either genotype (Additional file 1: Fig. S7a-c). *A*. *jandaei* TP531 association (10^4^ CFU/mL) also elicited an elevated NF-κB signaling in GITs of WT monitored by *Tg*(*NFκB:EGFP*) (Additional file 1: Fig. S7d, 7e), suggesting that *A*. *jandaei* TP531 is an inflammation-stimulatory pathobiont. Of note, no further significant increase in NFκB-EGFP expression in *tp53* mutants was observed by the same dose-exposure to *A*. *jandaei* TP531 (Additional file 1: Fig. S7d, 7e), presumably due to saturation of the NFκB-EGFP response at this dose and considering the pre-existing *Aeromonas* spp. enrichment in *tp53*-mutant GITs. Despite this, the colonization efficiency of *A*. *jandaei* TP531 in *tp53* mutant GITs was clearly distinct from that seen in WT GITs at the same bacterial-exposure dose (10^4^ CFU/mL) (see below).

The sensitized responses of the *tp53* mutants to *A*. *jandaei* TP531 may have resulted from their enhanced ability to colonize in the GITs of *tp53* mutants. To test this possibility, *A*. *jandaei* TP531 colonization using different titers was visualized directly using *mCherry*-tagged *A*. *jandaei* TP531 (*A*. *jandaei* TP531*-mCherry*)*. A*. *jandaei* TP531*-mCherry* exhibited a strong tendency to colonize the GITs of *tp53* mutants more efficiently than WT GITs (Fig. 3e, f). *A*. *jandaei* TP531*-mCherry* also survived better and became preferentially enriched in *tp53* mutant GITs. This was also confirmed by bacterial-culture colony counts, where the number of *mCherry*-positive *A*. *jandaei* TP531 colonies from dissected *tp53*-mutant GITs was higher compared to the WT number (Fig. 3f), while the total number of bacterial colonies was comparable for each genotype (Fig. 3g). These data collectively suggest that *A*. *jandaei* TP531 is a pathobiont that possesses an aggressive colonizing ability and that can induce significant responses, particularly in the *tp53* mutant intestine.

### The elevated NF-κB signaling is caused primarily by changes to the gut microenvironment in *tp53* mutants, not by dysbiotic microbiota

**Fig. 4:**
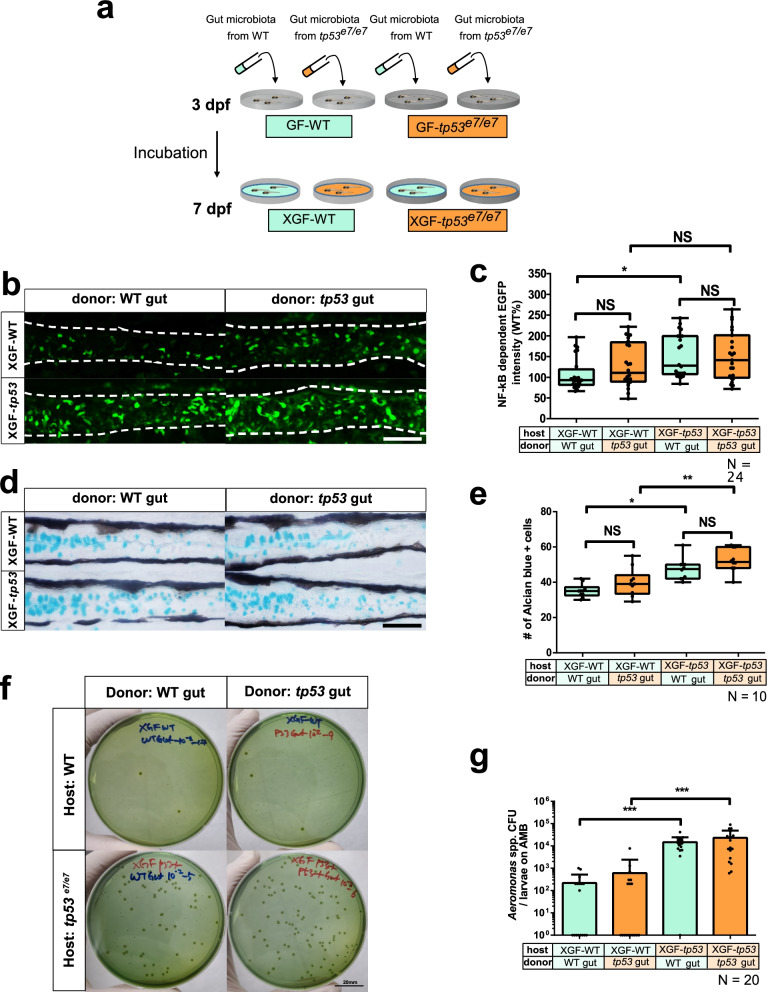
*tp53* mutation of the host, not the intestinal bacteria, plays a deterministic role in elevated NF-κB signaling and increased goblet cell numbers. **a** A schematic for ex-GF experiments where GF-grown WT or *tp53* mutant embryos were associated with 7 dpf microbiota of CR WT or *tp53* mutants at 3 dpf and examined for their responses at 7 dpf. Final density of microbiota was approximately 10^4^ CFU/mL, as assayed by aerobic growth on TSA plates at 37 °C. **b** Representative confocal images of the *Tg(NFκB:EGFP)*-based NF-κB signaling of ex-GF experiments. White dashed lines denote boundaries of GITs based on DIC bright field images. **c** A boxed plot of *NFκB*-dependent EGFP fluorescence intensity in ex-GF experiments. Significant differences were identified only in ex-GF *tp53* mutant host associated with WT microbiota. *N* = 24 each. **d** Representative images of Alcian blue-positive goblet cells of ex-GF experiments. **e** A boxed plot of the Alcian blue-positive goblet cell number in ex-GF experiments. *N* = 10 each. **f** Representative pictures of bacterial cultures from ex-GF experiments using *Aeromonas* medium base (AMB) plates. *Aeromonas* spp. was identified based on manufactural criteria (refer to Methods for details). Scale bar = 20 mm. **g** A bar graph showing a comparison of the *Aeromonas* spp. CFUs of the ex-GF experiment in AMB plates. The number of *Aeromonas* spp. colonies in *tp53* mutants host associated with WT or *tp53* mutant microbiota dramatically increased, whereas those of WT host did not. *N* = 20 each. The boxed plots and the bar graph were statistically estimated by non-parametric Friedman test followed by Dunn’s multiple comparisons test. Data are represented as mean ± SEM. *< *p* = .05; **< *p* = 0.01; ***< *p* = 0.005. *NS* not significant

Although our evidence demonstrated that *tp53* mutants exhibited an elevated intestinal immune response associated with dysbiosis, in which pro-inflammatory pathobionts (e.g., *A*. *jandaei* TP531) are selectively enriched, it is unclear whether the dysbiotic microbiota or the *tp53* host microenvironment is sufficient to elicit the increased inflammatory response. We addressed this by performing ex-GF experiments in which GF-grown WT or *tp53* mutant hosts were, in parallel or reciprocally, associated with the gut microbiota from 7 dpf WT or from *tp53* mutants beginning at 3 dpf, with any change of NF-κB signaling assessed at 7 dpf (Fig. 4a). NF-κB signaling activity in WT hosts resulting from association with dysbiotic microbiota of 7 dpf *tp53* mutant GITs (Fig. 2) was not significantly different from the WT host responses to original WT microbiota (upper panels in Fig. 4b, d; comparisons of the first two columns in Fig. 4c, e), indicating that the *tp53* mutant dysbiotic microbiota is not sufficient to induce aberrant NF-κB signaling. In contrast, the association of 7 dpf WT bacteria with *tp53* mutant hosts elicited a significantly increased NF-κB signaling response (left panels in Fig. 4b, d; comparisons of the first and third columns in Fig. 4c, e), suggesting that the microenvironment provided by *tp53* mutant hosts is crucial for enhanced NF-κB signaling. The association of *tp53* mutant hosts to *tp53* mutant bacteria did not further the response, presumably due to signaling saturation (bottom panels in Fig. 4b, d; comparisons of the last two columns in Fig. 4c, e).

We attempted to further confirm the preferential growth of intestinal *Aeromonas* spp. in the favorable microenvironment provided by the *tp53* mutant host. To test this, we first cultivated the microbiotas of both WTs and *tp53* mutants using plates with a selective medium referred to as the *Aeromonas* medium base (AMB), and confirmed that *Aeromonas* spp*.* was highly enriched in the *tp53* mutants (left panels in Fig. 6e; the first two columns in Fig. 6f). We then identified the species as *Aeromonas veronii* using genus-specific PCR primers and a 16S rRNA gene sequence (Additional file 1: Fig. S8a, b) that was consistent with our previous findings (Fig. 3; Additional file 1: Fig. S5). Then, we compared the intestinal *Aeromonas* spp. cultures under the aforementioned different ex-GF conditions using AMB plates. As a result, *Aeromonas* spp. CFUs from *tp53* mutant hosts associated with 7 dpf WT or *tp53* mutant microbiota increased dramatically (bottom panels in Fig. 4f; the last two columns in Fig. 4g), whereas those from WT hosts did not, irrespective of the origin of the associated microbiota (upper panels in Fig. 4f; the first two columns in Fig. 4g). These ex-GF experiments strongly support the notion that microenvironmental changes in the *tp53* mutant host play both causal and initiating roles in eliciting an aberrant inflammatory response, via providing pathobionts a selective advantage for colonization.

### The SA metabolism pathway is compromised in *tp53* mutants

**Fig. 5:**
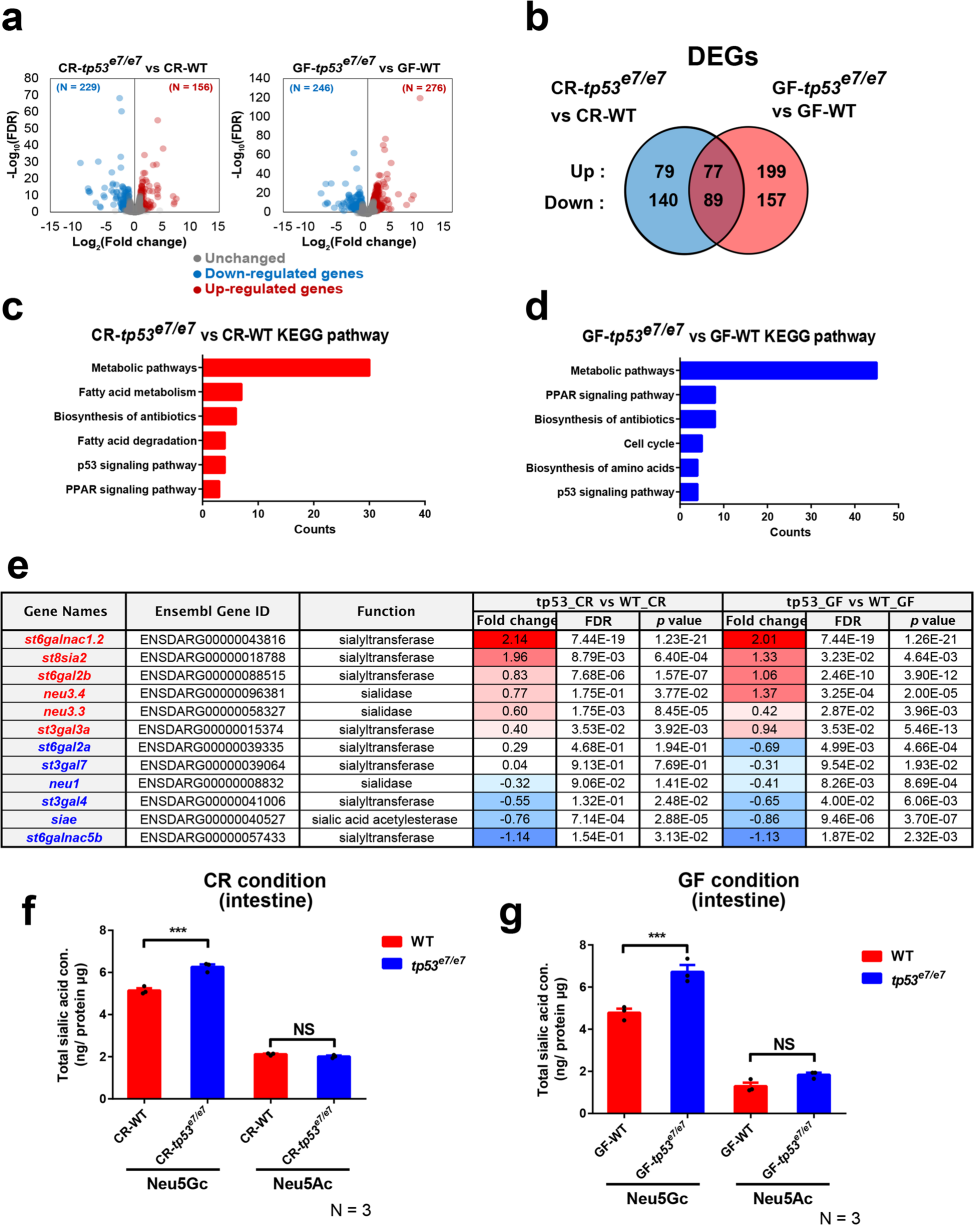
Sialic acid metabolism pathway is compromised in *tp53* mutants. **a** The volcano plots illustrating the comparison of the expression signature of the GITs of WT and *tp53* mutants under CR- or GF- conditions at 7 dpf. Each sample was repeated two times. The colored dots show the differentially up- (red) or down- (blue) regulated or unchanged (gray) genes in the GITs of *tp53* mutant compared with WT under CR- or GF- conditions at 7 dpf (|log_2_FC| > 1, FDR < 0.05). **b** Ben diagram illustrating that 385 (156 upregulated and 229 downregulated) or 522 (276 upregulated and 246 downregulated) differentially expressed genes (DEGs) were identified when CR-WT vs. CR-*tp53* mutants or GF-WT vs. GF-*tp53* mutants was compared, respectively (|log2FC| > 1, FDR < 0.05). 166 DEGs were common between those two conditions. **c**, **d** KEGG pathway analyses of CR-WT vs. CR-*tp53* mutants (**c**) or GF-WT vs. GF-*tp53* mutants (**d**) revealed major altered pathways under each comparison, with a change in metabolic pathways most affected. **e** A list of DEGs implicated in sialic acid metabolism. Fold changes (log2 scale) in red or blue denote up-regulated or down-regulated expression, respectively, with the color intensity correlated with the magnitude of fold changes. **f**, **g** HPLC-FLD analyses of total Neu5Gc and Neu5Ac amounts of WT or *tp53* mutant GITs under CR (**f**)- or GF (**g**)- condition. Bar graphs were statistically estimated by one-way ANOVA followed by Newman-Keuls multiple comparisons test. Data are represented as mean ± SEM. *N* = 3 each. ***< *p* = 0.005. *NS* not significant

To explore potential host microenvironmental changes in *tp53* mutants that may support the colonization of pathobionts and drive dysbiosis in *tp53* mutants, comparative transcriptomic analyses of WT GITs vs. *tp53* mutant GITs and CR GITs vs. GF GITs at 7 dpf were performed (Fig. 5a). We identified 385 transcripts (156 upregulated and 229 downregulated in *tp53* mutants compared to WTs) as differentially expressed genes (DEGs) for the CR condition, whereas 522 DEGs (276 upregulated and 246 downregulated) were found in the GF-conditioned WT and *tp53* mutants. Among these, 166 DEGs (89 upregulated and 77 downregulated) were commonly found under both CR and GF conditions (|log2FC| > 1, FDR < 0.05; Fig. 5b). The detailed list of DEGs is shown in Additional file 2. Initial gene ontology analysis revealed an increased signature of NF-κB signaling genes in CR *tp53* mutants (Additional file 1: Fig. S9b) consistent with heightened NF-κB signaling activity (Fig. 1a). In addition, the increased expression of *ikbaa*, a known direct target gene of NF-κB [57], in GITs of *tp53* mutants under CR conditions also have a tendency to decrease compared to that under GF conditions shown by quantitative RT-PCR (Additional file 1: Fig. S9c), consistent with the NF-κB-EGFP signal results. As expected, the *tp53* gene mutation (M214K mutation, ATG > AAG) was confirmed in *tp53* mutant samples (Additional file 1: Fig. S9a).

KEGG-pathway database analyses (https://www.genome.jp/kegg/kegg.html) identified that “metabolic pathways” was the primary category changed in the transcriptomes of both CR- and GF-conditioned *tp53* mutant GITs (Fig. 5c, d), which included the metabolisms of both carbohydrates (Additional file 1: Fig. S9c) and lipids (Additional file 1: Fig. S9d). Previous studies have suggested changes in host carbohydrate metabolism in patients with IBD and CRC, affected by the overpopulation of mucolytic bacteria [65] and a metabolic shift of the CRC patient microbiome from using dietary fiber to using host glycans during disease progression [27]. As *tp53* mutations are implicated as crucial in both IBD and CRC [10–12, 66], it is possible that carbohydrate metabolism may be compromised in *tp53* mutants. As a major carbohydrate metabolism pathway, changes to short-chain fatty acid (SCFA) metabolism (essential for host-bacterial interactions and immune modulation [67]) were examined by measuring the levels of acetate, propionate, and butyrate in WT and *tp53* mutants at 7 dpf using GC-MS (Additional file 1: Fig. S10). However, no significant differences were observed between the two genotypes (Additional file 1: Fig. S10a–c), suggesting the unlikely involvement of SCFAs in the dysbiosis of *tp53* mutants.

Next, we examined sugar metabolism as another pathway candidate, with a particular focus on SAs, a family of essential nine-carbon monosaccharides that coat host intestines predominantly at the non-reducing end of glycan chains on glycoproteins. SAs are known to be crucial for host-bacteria interactions by regulating host metabolism, bacterial survival, and immune surveillance [42]. Several genes involved in host SA metabolism were differentially expressed between WT and *tp53* mutants under both CR and GF conditions including sialyltransferases (e.g., *st6galnac1.2*, *st8sia2*, and *st6gal2b*) and sialidases (e.g., *neu3.3* and *neu3.4*) (Fig. 5e), implying aberrant SA metabolism in a *tp53-*dependent and microbiota-independent manner. The major SAs identified in the GITs of zebrafish larvae were *N*-glycolylneuraminic acid (Neu5Gc) and *N*-acetylneuraminic acid (Neu5Ac) (Additional file 1: Fig. S11a). The amounts of Neu5Gc and Neu5Ac in dissected GITs from WT and *tp53* mutants at 7 dpf were measured directly using HPLC with fluorescence detector (HPLC-FLD). Neu5Gc levels in *tp53* mutant GITs were aberrantly increased compared to levels in WT GITs under both CR and GF conditions, whereas Neu5Ac amounts did not differ significantly (Fig. 5f, g). This is consistent with the abnormal expression signature of sialometabolic genes in *tp53* mutants independent of microbiotal presence (Fig. 5e), further supporting a role for increased SAs, specifically Neu5Gc, in the *tp53* mutant host during dysbiosis and inflammation.

### The overgrowth of gut-inflaming *Aeromonas* spp. in* tp53* mutants requires their consumption of increased SAs

**Fig. 6:**
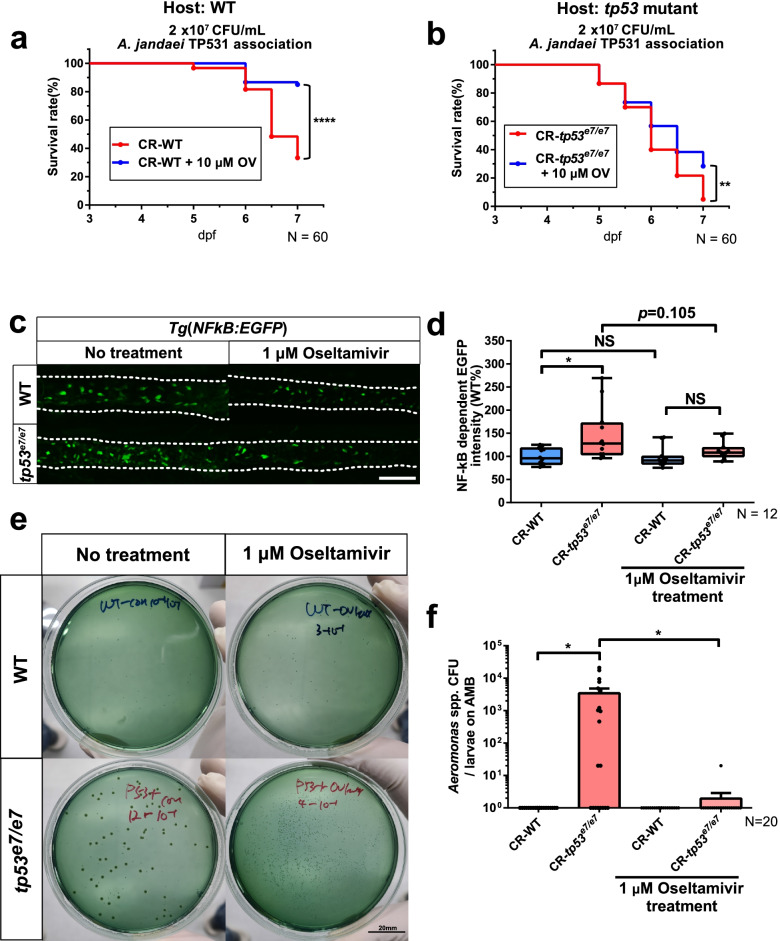
Overgrowth of endogenous *Aeromonas* spp*.* in *tp53* mutants requires a sialidase activity. **a**, **b** A lethality curve of *Aeromonas jandaei* TP531 association (2 × 10^7^ CFU/mL) with WT (**a**) or *tp53* mutant (**b**) larvae upon the treatment of oseltamivir (10 μM) starting at 3 dpf. *N* = 60 each. The lethality curve was statistically estimated by Gehan-Breslow-Wilcoxon test. **c** Representative images showing that oseltamivir (1 μM) treatment dampened an elevated inflammatory response in *tp53* mutants monitored by *Tg(NFκB:EGFP)*. White dashed lines denote boundaries of GITs based on DIC bright field images. Scale bar = 50 μm. **d** A boxed plot of changes of *NFκB* dependent EGFP intensities in the mid-distal intestines of WT and *tp53* mutants upon oseltamivir treatment. *N* = 12 each. **e** Representative pictures of bacterial cultures from WT or *tp53* mutants using *Aeromonas* medium base with or without oseltamivir treatment. *Aeromonas* spp. was identified based on manufactural criteria (refer to “Methods” for details). Scale bar = 20 mm. **f** A bar graph showing that the dramatically increased CFU numbers of *Aeromonas* spp. in *tp53* mutants were abrogated upon treatment of oseltamivir (1 μM). The boxed plot and the bar graph were statistically estimated by non-parametric Friedman test followed by Dunn’s multiple comparisons test. Data are represented as mean ± SEM. *N* = 20 each. *< *p* = 0.05; **< *p* = 0.01; ****< *p* = 0.001. *NS* not significant

*A*. *jandaei* TP531 is a pro-inflammatory pathobiont that selectively utilizes Neu5Gc as a carbon source (Additional file 1: Fig. S12a, 12b) and colonizes/survives better in *tp53* mutants (Fig. 3) with aberrantly increased Neu5Gc levels (Fig. 5f, g). We therefore hypothesized that *A*. *jandaei* TP531 colonization/survival would be suppressed if the availability of free SAs (presumed to originate from host glycans) were to be reduced. To test this possibility in our model, we administered oseltamivir (OV), a specific and FDA-approved sialidase inhibitor that prevents the release of SAs from glycan chains. With this inhibitor, larval lethality (both WTs and *tp53* mutants) after a high-exposure dose of *A*. *jandaei* TP531 (2 × 10^7^ CFU/mL) was significantly reduced by 10 μM OV treatment (Fig. 6a, b). Moreover, the enhanced NF-κB signaling and the enrichment of endogenous *Aeromonas* spp. in CR *tp53* mutants, monitored by *Tg*(*NFκB:EGFP*) and AMB plates, respectively, were significantly suppressed by 1 μM OV treatments (Fig. 6c, d and e, f, respectively). We also examined the effect of OV treatment on exogenously applied *mCherry*-tagged *A*. *jandaei* TP531 (1 × 10^4^ CFU/mL) (Fig. 3). The increased *mCherry* fluorescence, the enhanced inflammatory response, and the increased survival of *A*. *jandaei* TP531 in *tp53* mutants were nearly all abolished by 1 μM OV treatment (Additional file 1: Fig. S13), suggesting an essential role for sialidase activity in the preferential attachment, colonization, and/or survival of these bacteria in *tp53* mutant GITs. The total colony numbers using agar media (TSA plates) were not statistically different (Additional file 1: Fig. S13d), indicating the specific suppression of *A. jandaei* TP531 survival/colonization by OV treatment without disturbing overall microbial robustness and fitness. Taken together, these results indicate an essential role for sialidase activity in the enrichment of endogenous *Aeromonas* spp. as well as the overgrowth of exogenous *A*. *jandaei* TP531.

**Fig. 7:**
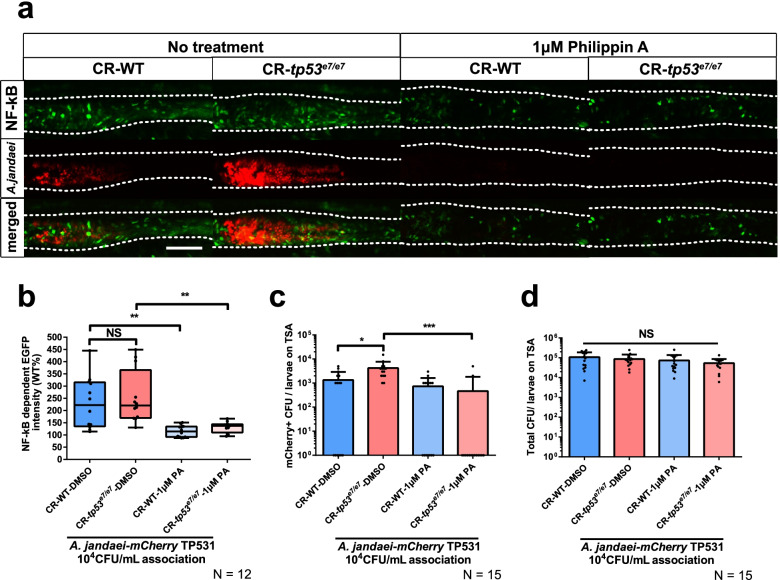
Elevated NF-κB signaling by exogenous *Aeromonas* in *tp53* mutants was abolished by Philippin A derived from nature product. **a** Representative images of elevated NF-κB signaling and colonization by exogenously added *mCherry*-tagged *A*. *jandaei* TP531 in WT and *tp53* mutants with or without Philippin A (1 μM) treatment. White dashed lines denote boundaries of GITs based on DIC bright field images. Scale bar = 50 μm. **b** A boxed plot showing that elevated NF-κB signaling monitored by *Tg(NFκB:EGFP)* in the mid-distal intestines with *mCherry*-tagged *A*. *jandaei* TP531 was abolished upon Philippin A treatment. **c** A bar graph showing that the enhanced survivability of *mCherry*-tagged *A*. *jandaei* TP531 in *tp53* mutants was abolished upon Philippin A treatment. **d** A bar graph showing that Philippin A treatment did not significantly affect total bacterial populations on TSA plates. The boxed plot and bar graph were statistically estimated by non-parametric Friedman test followed by Dunn’s multiple comparisons test. Data are represented as mean ± SEM. *< *p* = 0.05; **< *p* = 0.01; *NS* not significant

To test whether or not host sialidases are the targets of OV treatment, we examined the amounts of free and total SAs from whole-body specimens at 7 dpf, with or without OV treatment, using HPLC-FLD (Additional file 1: Fig. S11b for the experimental procedure). Both free and total whole-body levels of Neu5Gc in *tp53* mutants were aberrantly increased similar to the total GIT Neu5Gc levels observed for *tp53* mutants (Additional file 1: Fig. S11c, e; Fig. 5f). Importantly, despite the strong suppression of *Aeromonas*-associated lethality and of pro-inflammatory phenotypes by OV treatment (Fig. 6; Additional file 1: Fig. S13), there were no significant differences in free and total Neu5Gc levels with or without 1 μM OV treatment (Additional file 1: Fig. S11c, e; Fig. 5f), suggesting that host sialidases are unlikely to be the OV targets responsible for the phenotypic changes. As a possible alternative origin for the sialidase activity, we tested for the involvement of bacterial sialidases by using Philippin A (PA), a known potent bacterial sialidase inhibitor isolated from the root of *Flemingia philippinensis* [68]. PA inhibition results from a non-competitive mechanism against bacterial sialidases, and differs from the competitive inhibitory mechanism of OV [69]. Similar to the effects of OV treatment (Fig. 6; Additional file 1: Fig. S13), the elevated NF-κB signaling after associating *mCherry*-tagged *A*. *jandaei* TP531 (1 × 10^4^ CFU/mL) was significantly suppressed (Fig. 7a, b) and the number of *mCherry*-tagged *A*. *jandaei* TP531 colonies was dramatically reduced after 1 μM PA treatment (Fig. 7c). Total colony counts were not statistically different between the different conditions (Fig. 7d). Therefore, these results collectively suggest that bacterial sialidase activity is responsible for both the overgrowth of this pro-inflammatory pathobiont and the increased inflammatory response in *tp53* mutants, and its chemical inhibition suppresses these phenotypes without impacting overall microbial community density.

Finally, we tested whether exogenous Neu5Gc supplementation (already increased in *tp53* mutants) could mimic the enrichment of pathobionts in *tp53* mutants. When WTs and *tp53* mutants under CR conditions were supplemented with 250 μg/mL of exogenous Neu5Gc, the NF-κB-EGFP activity was significantly increased in GITs of both groups (Additional file 1: Fig. S14a, b). Similarly, the exogenous supplementation of Neu5Ac (250 μg/mL) to these same groups also significantly increased NF-κB signaling in WT GITs under CR conditions, but not under GF conditions (Additional file 1: Fig. S15a-d), indicating that Neu5Ac supplementation can elicit a pro-inflammatory response in a microbiota-dependent manner. Contrary to our expectations, however, the growth of *Aeromonas* spp. in both genotypes on AMB plates was only marginally changed after Neu5Gc supplementation (Additional file 1: Fig. S14c) or was even suppressed by Neu5Ac supplementation (Additional file 1: Fig. S15e). These seemingly contradictory results may be explained by the observation that *Citrobacter* spp. are distinct species of bacteria enriched in *tp53* mutants (Additional file 1: Fig. S5; S8), and an isolated strain *Citrobacter freundii* TP531 utilized the free forms of Neu5Gc and Neu5Ac more efficiently (Additional file 1: S16c-e), and therefore outcompeted *Aeromonas* spp. growth during the supplementation experiments, and was presumably responsible for the majority of the activated NF-κB signaling. In addition, incubation of OV or PA with *A*. *jandaei* TP531 did not affect its growth in the presence of either Neu5Gc or Neu5Ac *per se* (Additional file 1: S16f, g), indicating that the inhibitory effect of sialidase inhibitors on *A. jandaei* TP531 blooming in vivo is unlikely due to the direct inhibition on the bacteria. This unexpected finding reflects the complexity of microbe-host and microbe-microbe interactions mediated by sialo-metabolites and warrants further study as to how these sialic acids in the microenvironment can be utilized by pathobionts in vivo.

## Discussion

Chronic inflammation in the colon (colitis) can induce intestinal dysplasia and increase the incidence of CRC. Based on previous findings revealing associations between host *TP53* mutations and chronic inflammation during the early stages of CRC, and between intestinal dysbiosis and CRC, we reasoned that such early host *TP53* mutations may promote inflammation and subsequent CAC pathogenesis by causing dysbiosis in the intestinal microbiota. Here, we discovered increased intestinal inflammation in *tp53* mutant zebrafish model due to microbiota dysbiosis, which featured the selective and preferential blooming and colonization of pro-inflammatory pathobionts (e.g., *Aeromonas* spp.). Moreover, the *tp53* host mutation promoted dysbiosis through the supply of excess intestinal Neu5Gc (presumably derived from *tp53* mutant intestinal glycans) which was exploited by pathobionts for overgrowth. This finding is also supported by the observation that inflammation was decreased in *tp53* mutants when sialidase activity, likely of bacterial origin, was pharmacologically inhibited. Taken together, our results reveal the unappreciated role of host *tp53* in modulating intestinal symbiosis, and the importance of host *tp53* mutation-driven dysbiosis in the inflammatory response mediated by sialometabolism. Based on these findings, we propose a potential therapeutic approach for treating both chronic inflammatory diseases and CAC associated with *TP53* mutations by employing sialidase inhibition-based microbial manipulations.

### Mutation of *tp53*, a host genetic factor, is conducive for dysbiosis in the intestinal microbiota

Interactions between the host and the microbiota are essentially bidirectional: the gut microbiome residing in the host’s intestine produces metabolites and cell-associated molecules that affect host cells, while the host provides environmental niches and nutrients, producing immunoglobulin A, antimicrobial peptides, and microRNAs necessary for the survival and maintenance of the microorganisms [32, 70, 71]. Despite these symbiotic interactions, evidence supporting a causal role for host genetics to shape diversity in the gut microbiota does exist. For example, a cross-species association (microbiota swapping) study between zebrafish and mice showed that the zebrafish gut microbiota associated with GF mice was modified into an indigenous mouse-gut microbial community [72]. Likewise, in *Fut2* knockout mice, where host terminal-fucose moieties are absent, dysbiosis (decreased alpha-diversity) of the microbiota was observed [73]. Furthermore, in a human-twin cohort study, the microbiomes of monozygotic twins were found to be more similar than those of dizygotic twins [74]. In metagenome–genome-wide association studies, host-gene mutations (e.g., *NOD2*, *FUT2*, *CARD9*, and *LCT*) have been implicated in IBD and associated microbiomes, emphasizing the strong association between host genes and microbiome bacteria [70].

In CAC, both the genetic status of the host (e.g., *tp53* mutations) and dysbiosis in the microbiota have separately been shown to contribute to chronic inflammation and to cancer development [31, 75], but whether a host genetic factor, such as the present *tp53* mutation, can alter the composition of the microbiota and lead to inflammation (and ultimately tumorigenicity) has not been investigated. In the present larval zebrafish model, we have demonstrated that a host *tp53* mutation is a causative genetic factor that can consequentially lead to the microbiota dysbiosis responsible for initiating intestinal inflammation. This conclusion is based on the findings that (1) the indigenous intestinal dysbiosis in *tp53* mutant larvae resulted in inflammation, while changes in essential environmental contributions such as feeding were excluded since no feeding is required for the survival of zebrafish larvae until 7–8 dpf when most of experiments were performed (Figs. 1 and 2); (2) the host *tp53* mutation, not the dysbiotic microbiota, dictated the blooming of *Aeromonas* spp. and the inflammatory response as shown by ex-GF experiments using reciprocal association of WT and *tp53* mutant (Fig. 4); and (3) candidate pathobionts such as *Aeromonas* spp. were highly enriched specifically in *tp53* mutant GITs, with *A. jandaei* TP531 exhibiting colonization advantages (Figs. 3 and 7; Additional file 1: Fig. S13) by exploiting aberrant SA metabolism (Figs. 5, 6, and 7; further discussion below). Thus, we propose that such a host *tp53* mutation is a crucial genetic factor that can determine microbiota dysbiosis in chronic inflammation and in CAC. Once inflammation is initiated by the *tp53* mutation-induced dysbiosis, a vicious cycle may ensue where this inflammation then aggravates dysbiosis, similar to the inflammatory responses triggered by bacterial infections, genetic predisposition (*IL10* or *NEMO* knockout mice), and chemical treatments (such as DSS) that can induce intestinal dysbiosis [63, 76]. The interactions between host genetics, the environment, the microbiota, and inflammation are very complex, and it remains a challenge to fully appreciate the contribution of each in detail. The use of larval zebrafish as a whole-animal model offers the convenience of being able to exploit genetic manipulations and the accessibility of *in vivo* imaging, and the possibility to maintain GF individuals without exogenous feeding for up to 7–8 dpf, which is advantageous for the comprehensive dissection of these interactions, especially in terms of possible genetic contributions.

### Intestinal microbiota dysbiosis and overgrowth cause an inflammatory response via a SA metabolism imbalance

In host cells, SAs are generally incorporated as terminal sugar components in the glycans of cell membranes where SAs play essential physiological and pathological roles in the interactions and signaling events between host immune cells and microbes as well as between host cells [77]. In various types of epithelial cancers, the SA-containing and aberrantly truncated *O*-glycan STn onco-antigen and the key STn-synthesizing sialyltransferase, *ST6GALNAC1*, were shown to be overexpressed, with the STn antigen expressed at early stages of tumorigenesis [78]. The STn antigen not only functions in regulating tumor proliferation, metastasis, and immune evasion but is also used as a cancer biomarker and therapeutic target [40, 78]. In addition, polysialic acids (PSA), primarily synthesized by the polysialyltransferase *ST8SIA2*, are often overexpressed in aggressive and invasive cancers with poor prognoses [39, 79]. Despite their significance in cancer development, a direct relationship between upregulated STn/*ST6GALNAC1*, PSA/*ST8SIA2*, and *TP53*, “the guardian of the genome,” has been rarely considered, except for *TP53* mutants being implicated in the *N*-glycosylation of proteins via the UDPase, ENTPD5 [45], and the correlated expressions of STn and Tp53 protein in a mouse model of bladder cancer [44].

In the current study, we provide firm evidence that a *tp53* mutation indeed upregulated host SA metabolism-related genes, including *st6galnac1.2* and *st8sia2* (Fig. 5), and increased Neu5Gc amounts in CR and GF GITs from *tp53* mutants (Fig. 6), likely concomitant with the formation of STn- or PSA-glycan structures. These SA-enriched and STn- or PSA-coated *tp53* mutant cells may be pro-inflammatory, as shown in IBD patient samples [80], and acquire advantages for cancer progression due to increased proliferation, genomic instability, and metastatic activity via STAT3 and NF-κB signaling [40, 78, 81]. Importantly, increased SA metabolism-associated gene expression and Neu5Gc levels in *tp53* mutants were independent of the microbiota (Figs. 5 and 6), but the elevated inflammatory response was nearly blocked under GF conditions (Fig. 1) and by OV treatment which resulted in a reversal of *Aeromonas* spp. abundance (Fig. 6). Therefore, we propose that the SA-enriched *tp53* mutant cells are pro-inflammatory primarily because of dysbiotic gut microbiota induced by aberrant sialometabolism. In other words, SA-utilizing pathobionts such as *Aeromonas* spp. exploit the mutation-inherent and SA-enriched microenvironment of the host *tp53* mutant intestine for their overgrowth, thereby triggering dysbiosis and eliciting detrimental inflammation. The cascade of these events (*tp53* mutation inducing aberrant SA metabolism, followed by pathobiont blooming and inflammation) may be a novel mechanistic explanation for the role of *tp53* mutations at early stages of CAC progression [11, 12], and may also be consistent with a metabolic shift to host glycan utilization by the human CRC microbiome during cancer progression [27].

Of note, it has been established that aberrant accumulation of Neu5Gc promotes low-level chronic inflammation leading to cancer progression in human tumor cells [82, 83], similar to the increased Neu5Gc and elevated inflammation in zebrafish *tp53* mutant intestines in our current study. In contrast to zebrafish, humans cannot synthesize Neu5Gc because humans lack a functional cytidine monophospho-*N*-acetylneuraminic acid hydroxylase (CMAH) enzyme that converts Neu5Ac to Neu5Gc [84]. Chronic inflammation in humans may be elicited by incorporating exogenous Neu5Gc into tumor cells and interaction of auto-antigen Neu5Gc and auto-antibodies against Neu5Gc-containg glycans [83, 85]. Therefore, despite the commonality of Neu5Gc-mediated elevation of inflammation between human and zebrafish *tp53* mutants, the detailed underlying mechanisms appears to be different in that inflammation in zebrafish is caused by blooming of pro-inflammatory pathobionts capable of selectively utilizing Neu5Gc (Figs. 1 and 3; Additional file 1: Fig. S16), although these mechanisms may not be mutually exclusive. Currently, the contribution of the functional *cmah* in zebrafish to elevated inflammation in *tp53* mutants is unclear, and needs to be addressed with a further study using a zebrafish *cmah* knockout mimicking the human condition.

The present experiments confirm the *tp53* mutation as a causative host factor for the inflammatory response in a genetically predisposed and pathologically relevant in vivo context, with the potential for tumorigenesis progression. Moreover, our study illustrates the importance of interactions between host factors and the microbiota which culminate in dysbiosis mediated by a sialometabolic imbalance resulting from host genetics. Our findings can be extended to a previous study of DSS-induced colitis in a mouse model that reported the crucial dysbiosis involvement of SA catabolism utilized by *E*. *coli* for inflammation [43]. A deeper understanding of the complex interactions between host genetics, the environment, the microbiota, and inflammation, especially via essential carbohydrate metabolites such as SAs both as nutrients and as signaling molecules, will reveal their under-appreciated roles in pathogenesis as well as in development and normal physiology. This better understanding will eventually inform the design of more efficient and targeted therapies based on manipulating these interactions.

Given that SA and its diverse derivatives are rarely synthesized by bacteria, they need to be released from host glycans by sialidases to become freely available for import into the bacterial cytosol either to undergo sialocatabolic processing or to mask the bacteria for host immune-surveillance evasion [42]. Intriguingly, the origin of such sialidase activity that liberates SAs from host glycans for pathobiont colonization is unclear, although its requirement was evident in the OV- and PA-treatment experiments in our study (Figs. 6 and 7, and Additional file 1: Fig. S13). Considering that no differences between free and total whole-body SA levels of the whole body were observed after OV treatment (Additional file 1: Fig. S11) and the known instability of host sialidases in the extracellular matrix [86], host sialidases may not have been the target for OV suppression. Rather, the efficient suppression by PA treatment (Fig. 7) suggests bacterial sialidase(s) are implicated in this process. Although it is formally possible that the overpopulating *Aeromonas* spp. in *tp53* mutants provided the sialidase activity [87, 88], the mono-association of a lethal dose of *A*. *jandaei* TP531 with GF larvae hardly exhibited a lethal effect (Additional file 1: Fig. S17), arguing that the sialidase activity was conferred by other interacting bacteria of the dysbiotic microbiome. The identification of the responsible bacteria, and a detailed determination of the mechanism, warrants further investigation. This phenomenon is reminiscent of the interactions of *Salmonella typhimurium* and *Clostridium difficile* with *Bacteroides thetaiotaomicron* in antibiotic-induced pathogen expansion [89] and of *E*. *coli* with *Bacteroides vulgatus* in intestinal inflammation [43], although the present experimental conditions involving a *tp53* mutant background were endogenous and more physiologically relevant. On the other hand, since *tp53* mutations can affect multiple metabolic pathways such as production of reactive oxygen/nitrogen species [90] that may impact microbial population [91, 92], the possibility cannot be completely excluded that the sialometabolism-independent function of *tp53* mutation partly contributes to blooming of *Aeromonas* spp. Further in-depth studies to dissect detailed mechanisms will help to reveal the complete view of complex interactions of pathobionts and the host with *tp53* mutation.

## Conclusions

In this study, we found an unappreciated role of host *tp53* in modulating intestinal symbiosis using a zebrafish larvae model. The *tp53* mutation promoted dysbiosis by supplying excessive intestinal Neu5Gc and by supporting pathobiont (e.g., *Aeromonas* spp.) blooms. The increased inflammatory responses due to dysbiosis were efficiently blocked by treatment with specific sialidase inhibitors (OV and PA) that prevented the overgrowth of SA-utilizing pathobionts, presumably by decreasing the availability of free SAs. This cascade of events, promoted by the *tp53* mutation, are summarized in a proposed working model (Additional file 1: Fig. S18). Our data illustrate the importance of proper sialometabolism regulation by *TP53* to maintain symbiotic microbial diversity and to ensure appropriate immune responses. Once disrupted, however, the regulatory collapse of sialometabolism can result in dire consequences, including pro-inflammatory dysbiosis and ultimately susceptibility to the development of cancer. This scenario provides a plausible explanation for the initial involvement of *TP53* mutations in the early stages of CAC development. These findings not only reveal physiological interactions among *TP53*, the microbiota, and inflammation but also provide a valid and efficient therapeutic means for treating *tp53* mutation-driven inflammation and cancer progression based on manipulating sialometabolism followed by correcting dysbiosis.

## Methods

### Animal husbandry and genotyping *tp53*^*e7/e7*^

Zebrafish (*Danio rerio*) AB (wild-type, WT) strain and *tp53*^*e7/e7*^ mutant were maintained at 28.5 °C under standard condition. Fish were fed daily a combination of dry food and brine shrimp and maintained under a light schedule of 14 h light and 10 h dark. Zebrafish husbandry and animal care were performed in accordance with guidelines from the Korea Research Institute of Bioscience and Biotechnology (KRIBB) and approved by KRIBB-IACUC (approval number: KRIBB-AEC-17126).

Genotyping of *tp53*^*e7/e7*^ mutant zebrafish was performed as previously reported [59]. Briefly, genomic DNA was extracted individually from caudal fin clip by Direct PCR kit (Nanohelix) following the manufacturer’s protocol. Extracts were amplified by PCR using a forward primer 5′ACA TGA AAT TGC CAG AGT ATG TGT C-3′ and a reverse primer 5′-TCG GAT AGC CTA GTG CGA GC-3′. Amplified PCR products were digested by MboII (Takara) for 2 h. The mutation of *tp53*^*e7/e7*^ was determined by the resulting restriction pattern. 336bp sized PCR products containing the *tp53*^*e7/e7*^ mutation were cleaved to 140 bp and 196 bp.

### Gnotobiology

To derive gnotobiotic zebrafish, germfree condition was established as previously described [93]. Briefly, natural bred zebrafish eggs were collected in autoclaved gnotobiotic zebrafish medium (GZM) including 40 g/L Instant Ocean stock and 1.25 mL/L Bullseye 7.0 (Wardley). Fertilized eggs were incubated in antibiotic GZM with 250 ng/mL amphotericin B (Sigma-Aldrich), 100 μg/mL ampicillin (Sigma-Aldrich), and 5 μg/mL kanamycin (Sigma-Aldrich) for 6 h. Normally developed embryos were sorted using an SZX16 microscope (Olympus) and rinsed 3 times in autoclaved GZM to remove antibiotic chemicals in the hood. Next, embryos were disinfected with 0.1% polyvinylpyrrolidone-iodine (PVP-I, Sigma-Aldrich) for 2 min and washed 3 times in sterile GZM. Then, embryos were exposed to 0.003% sodium hypochlorite (Sigma-Aldrich) solution for 20 min and washed 3 times in sterile GZM. After disinfection, embryos were kept at 28.5 °C in E3 egg water (5 mM NaCl, 0.17 mM KCl, 0.33 mM CaCl_2_, 0.33 mM MgSO_4_) and changed with the autoclaved E3 egg water every day. To check the microbial contamination, 20 μL of E3 egg water growing gnotobiotic zebrafish were spotted everyday onto a TSA plate and incubated the plate aerobically at 37 °C for at least 5 days.

### Experimental designs

The layout for the experiments in the present study is shown in Fig. S2a (Additional file 1). Briefly, WT and *tp53* mutant natural bred eggs were incubated in conventionally raised (CR) condition or germfree (GF) condition following the proper procedure described above. Because the microbiota begins to colonize in the GITs at 3 dpf zebrafish after opening the mouth, chemicals or bacteria were treated at 3 dpf zebrafish. Detailed analyses were conducted at 7 dpf to reduce possible secondary effects derived from nutrient deprivation. Zebrafish larvae could be endurable at 14 dpf without food according to Bates JM, Mittge E, Kuhlman J, Baden KN, Cheesman SE and Guillemin K [94], but they start to exhibit intestinal necrosis due to nutrient deprivation after 9 dpf. All animal experiments were repeated at least twice with 10 zebrafish larvae per group and were performed at 28.5 °C.

### Confocal microscopic analyses for intestinal NF-κB activity analysis

To analyze intestinal NF-κB activity using *Tg*(*NFκB:EGFP*) with high resolution, *Tg*(*NFκB:EGFP*) of WT and *tp53*^*e7/e7*^ mutant larvae at 7 dpf in CR or GF condition with or without chemicals and bacteria treatment were fixed with 1X staining solution (4% paraformaldehyde (PFA, Sigma-Aldrich), 4% sucrose (Sigma-Aldrich), 0.15 mM CaCl_2_, 1× phosphate-buffered saline (PBS)) for overnight at 4 °C. Fixed embryos were washed briefly with 1× PBST and embedded on the glass-bottomed imaging dishes with 1% low melting point agarose. The mid-distal intestine that spans the region of 250 μm from the anus was imaged using FV1000 confocal microscope (Olympus) with the identical fluorescence laser condition. Confocal z-projections were made by stacking 10~11 sections with 5 μm thickness. Each group was expressed as a percentage relative to the mean of fluorescence intensity of WT zebrafish mid-distal intestines in CR condition using Image J software (NIH).

### Alcian blue staining

To measure alcian blue positive goblet cells, alcian blue staining was modified as previously reported [95]. WT and *tp53* mutant larvae in various conditions were fixed in 4% PFA for overnight at 4 °C and washed with 1XPBST at least 3 times. 0.05% Alcian blue solution with 1% HCl and 70% EtOH were incubated for 30 min. Then, specimens were cleared in acidic ethanol including 5% HCl, 70% EtOH for overnight at 4 °C. Next, Specimens were dehydrated in an ethanol series (25, 50, 75, and 100% ethanol) and stored in 100% glycerol at 4 °C before taking images. Alcian blue positive goblet cells in the mid-distal intestine corresponding to the region of 250 μm from the anus were counted using an SZX16 microscope and imaged with a TUCSEN Dhyana 400DC. To cover the total alcian blue positive goblet cells in the mid-distal intestines, at least 4 images having different z-axes were taken at the same location and stacked using a helicon focus 7 software (Heliconsoft).

### Cryosection and immunohistochemistry

To reveal intestinal NF-κB positive cells using *Tg*(*NFκB:EGFP*) in *tp53* mutant intestine, *tp53* mutant at 7dpf under CR condition were collected and fixed in 4% paraformaldehyde in 1× PBS. After overnight incubation at 4 °C, specimens were washed in 5% sucrose/1X PBS at room temperature and embedded in 1.5% agar with 5% sucrose. After solidifying agar, embedded specimens were soaked in 30% sucrose solution for overnight at 4 °C. The specimens were frozen in Surgipath FSC 22 (Leica) and maintained at – 80 °Cuntil sectioning at a thickness of 20 μm using CM1860 Cryostat Microtome(Leica). Frozen sections were dried at 50 °C for 2 h and subsequently stored at − 20 °C. Prior to immunohistochemistry, slides were thawed for 20 min at 50 °C. Sagittal sections were rehydrated in 1X PBS and incubated in blocking solution (1× PBS, 2% NGS (normal goat serum, Ambion), 0.4% Triton X-100, 2% DMSO). The sections were incubated overnight at 4 °C with the primary antibody diluted in blocking buffer. Primary antibodies used the mouse monoclonal Anti-Zebrafish Gut Secretory Cell Epitopes (2F11) antibody (1:200, Abcam), rabbit polyclonal anti Anti-Cadherin 1, epitherial (1:200, Genetex) for these studies. After overnight incubation, sections were washed in 1× PBST. The sections were then incubated for one hour at room temperature with the secondary antibody diluted 1:500 in 1× PBST. Secondary antibodies included Alexa Fluor conjugated goat anti-primary 488 and 594 (Molecular Probes). Sections were again washed in 1× PBST or 1× HBSS. 1× HBSS washed sections were re-stained with Alexa Fluor 647 conjugated wheat germ agglutinin (WGA, 5 μg/mL, Invitrogen) for 10 min at room temperature. After washed with 1× HBSS, sections were mounted using Vectashield mounting medium with DAPI (Vector lab). The mid-distal intestinal regions were imaged using FV1000 confocal microscope (Olympus) with the identical fluorescence laser condition.

### Ex-germfree experiment

The 20 GITs of WT and *tp53* mutant zebrafish larvae at 7 dpf under CR conditions were dissected and pooled in autoclaved E3 egg water using fine pins and forceps. After homogenizing by pestles (Sigma-Aldrich), the concentration of microbiota from 20 larvae GITs/20 mL E3 egg water was adjusted to become the final density 10^4^ CFU/mL, as assayed by aerobic growth on TSA at 37 °C. To generate ex-GF zebrafish larvae, WT and *tp53* mutant zebrafish maintained under GF conditions were associated with the intestinal microbiota from WT or *tp53* mutants E3 Egg water at 3 dpf and incubated for 4 days (Fig. 4a).

### Isolation and identification of bacteria altered in *tp53* mutants

Since the information of bacteria for isolation was lacking, intestinal bacteria were tried to be cultured in both aerobic and anaerobic conditions unbiasedly. In order to isolate enriched bacteria in *tp53* mutant GITs, first, 20 *tp53* mutant zebrafish GITs at 7 dpf under CR conditions were dissected using fine pins and forceps and pooled in sterile E3 egg water. After homogenized by pestles, GITs in suspension were serially diluted and plated on Aeromonas medium base (AMB, Oxoid) under aerobic conditions and reinforced clostridial medium (RCM, DB) under anaerobic condition at 37 °C. RCM supplemented with 1 mg/L of resazurin sodium salt for a redox indicator and maintained anaerobic conditions using anaerobic chamber (Don Whitley Scientific). A single colony with different morphologies was picked and seeded onto AMB or RCM and incubated at 37 °C under aerobic or anaerobic conditions. Single isolated colonies were used for further characterization by 16S rRNA gene sequencing. The full-length of 16S rRNA gene to identify isolated bacteria was amplified by primer pairs 27F (5′-AGA GTT TGA TCC TGG CTC AG-3′) and 1492R (5′-CGG TTA CCT TGT TAC GAC TT-3′) and sequenced at Bioneer (Daejeon, Korea) with Sanger sequencing. The complete 16S rRNA sequence of isolated bacteria was searched on BLAST (http://www.ncbi.nlm.nih.gov/BLAST/) to identify isolated bacteria.

### Incubation of isolated bacteria

In order to examine the pathogenicity of *Aeromonas jandaei* TP531 as a candidate pathobiont, WT and *tp53* mutant zebrafish was associated with the bacteria. First, a lethality test was performed using a high dose of bacteria. *A*. *jandaei* TP531, *E*. *coli* DH10B, and *P*. *damselae* DreWT1 were cultured in LB broth at 37 °C. *E*. *coli* was bought from Thermo Scientific company and *P*. *damselae* DreWT1 was isolated from GIT of WT zebrafish larvae (refer to Additional files 1: Method section for more details). *E*. *coli* and *P*. *damselae* DreWT1 were used as negative controls. Cultured bacteria were suspended using sterile E3 egg water. WT and *tp53* mutants at 3 dpf were exposed to a high dose of *A*. *jandaei* TP531 (2 × 10^7^ CFU/mL), *E*. *coli* DH10B (1 × 10^8^ CFU/mL) and *P*. *damselae* DreWT1 (1 × 10^8^ CFU/mL) by static immersion for 4 days, respectively. Survival of incubated larvae was checked at 12-h intervals. Next, to test intestinal inflammation response caused by isolated bacteria, a serial dilution of *A*. *jandaei* TP531 and *E*. *coli* DH10B was associated with WT and *tp53* mutants at 3dpf and examined at 7 dpf using Alcian blue-positive goblet cell counting and *Tg*(*NFκB:EGFP*) zebrafish. *P*. *damselae* DreWT1 (1 × 10^5^ CFU/mL) was examined inflammation responses using *Tg*(*NFκB:EGFP*) zebrafish. To directly visualize *A*. *jandaei* TP531, the *pACBB-mCherry* vector was introduced into *A*. *jandaei* TP531 by electroporation as previously reported [96].

To figure out where and how much *A. jandaei* TP531 was colonized, WT and *tp53* mutants at 3 dpf were associated with a serial dilutions of *mCherry*-tagged *A*. *jandaei* TP531 for 4 days. WT and *tp53* mutants with *mCherry*-tagged *A*. *jandaei* TP531 were imaged using the FV1000 confocal microscope and SZX16 microscope as described above. The colonization efficiency of *mCherry*-tagged *A*. *jandaei* TP531 in WT and *tp53* mutants were determined by the ratio of colonized *mCherry*-positive bacteria per 10 WT and *tp53* mutant GITs at 7 dpf. Each group was repeated four times. At the concentration of 10^4^ CFU/mL, where the biggest difference of the colonization efficiency between WT and *tp53* mutants were observed, CFU of total bacteria and *mCherry*-positive *A*. *jandaei* TP531 per larvae growing in WT and *tp53* mutants were calculated under the fluorescence microscope in TSA plates as describe above.

### Assessment of the number of *Aeromonas* spp. using AMB plates

To confirm the endogenous level of *Aeromonas* spp. under various conditions, *Aeromonas* spp. were counted using Aeromonas medium base (AMB) plates. Colonies with three distinct morphologies isolated from WT or *tp53* mutants were cultivated on AMB plates (Additional file 1: Fig. S8a). To determine which type of colonies belongs to *Aeromonas* spp., bacterial genomic DNA of the colonies with different morphologies were extracted and used as a template for PCR using pan bacteria- (27F/1492R primer) or *Aeromonas*-specific primer sets (*Aeromonas* 16S rRNA: forward primer (5′-GGG AGT GCC TTC GGG AAT CAG A-3′) / reverse primer (5′-TCA CCG CAA CAT TCT GAT TTG-3′); *Aeromonas* gyrB: forward primer (5′-GAA GGC CAA GTC GGC CGC CAG-3′) / reverse primer (5′-ATC TTG GCA TCG CCC GGG TTT TC-3′). Primer pairs of *Aeromonas* spp.-specific genes were designed and tested as previously published [97]. Amplicons of pan-bacteria (27F/1492R) were used to confirm the identity of the bacteria by Sanger sequencing. Also, the number of *Aeromonas* spp. (#1 bacteria) on AMB plates were counted under a bright field following manufactural criteria. Colonial features of *Aeromonas* spp. on AMB plates includes 0.5-1.5 mm diameter of size and appeared dark green with the opaque darker center in the middle.

### Supplementation of sialic acids

For supplementation of sialic acids Neu5Gc and Neu5Ac, WT and *tp53* mutants at 3 dpf under CR conditions were incubated with 250 μg/mL *N*-acetyl neuraminic acid (Neu5Ac,Tokyo chemical industry) and *N*-Glycolyl neuraminic acid (Neu5Gc, Carbosynth) for 4 days. To avoid side effects from acidic pH, both Neu5Ac and Neu5Gc were dissolved in E3 egg water and neutralized to pH 7 using 0.5 M KOH. After supplementation of sialic acids, inflammatory responses in the mid-distal intestines was monitored using *Tg*(*NFκB:EGFP*) transgenic zebrafish larvae and the number of *Aeromonas* spp. on AMB plates were counted.

### Treatment of sialidase inhibitors

To limit the availability of sialic acids in GITs, Oseltamivir phosphate (OV, Carbosynth) known as commercial sialidase inhibitor and Philippin A (PA) isolated from the root of *Flemingia philippinensis* were used. PA, reported as an inhibitor of bacterial sialidase [68], was dissolved in dimethyl sulfoxide. To confirm suppressive activity of OV on endogenously-colonizing, sialic acid utilizing bacteria, WT and *tp53* mutants at 3 dpf under CR conditions were treated with 1 μM OV for 4 days. In addition, to confirm suppressive activities of OV and PA against exogenously associated *Aeromonas jandaei* TP531 (1 × 10^4^ CFU/mL), WT and *tp53* mutants under CR conditions at 3 dpf were treated with 1 μM OV or PA for 4 days together with *A*. *jandaei* TP531 association. After treatment sialidase inhibitors, inflammation response in the mid-distal intestines was monitored using *Tg*(*NFκB:EGFP*) transgenic zebrafish larvae. The number of *A*. *jandaei* TP531 on TSA plates were counted *mCherry* positive colonies using SZX16 microscope with fluorescence filter (Ex 530–550 / Em 575). The number of *Aeromonas* spp. on AMB plates were counted under bright field following the proper procedure described above.

### Bacterial genomic DNA extraction and 16S rRNA gene sequencing

GITs of zebrafish larvae at 7 dpf were dissected as describe above and 30 larval GITs per group were pooled with three biological replicates for each group. Dissected specimens were mixed with STES buffer (0.5 M NaCl, 0.2 M Tris-HCl (pH7.6), 0.01 M EDTA, and 1% SDS in DNase/RNase free distilled water) to extract bacterial genomic DNA from zebrafish GITs. After bead beating to homogenize specimens, and overnight incubation at 60 °C, an equal volume of phenol:chloroform:isoamyl alcohol (25:24:1, v/v, Sigma-Aldrich) solution was added into the samples, mixed thoroughly, and centrifuged at 13,500 rpm. The supernatants were transferred into fresh tubes and mixed with an equal volume chloroform, followed by centrifugation, retrieval of the supernatants, and precipitation with a four-time volume of cold ethanol and another centrifugation at 4 °C for 20 min at 13,500 rpm. DNA pellets were washed with 70% ethanol and the dried bacteria genomic DNA was dissolved with DNase/RNase free water. The amounts and quality of quality were evaluated by NanoDrop 2000 spectrophotometer (Thermo).

16S rRNA amplicon sequencing was performed at Chunlab Inc. (Seoul, Korea) with MiSeq system (Illumina). Briefly, for preparation of MiSeq library amplicons, target gene (16S rRNA V3–V4 region) was amplified using 341F (5′-CCT ACG GGN GGC WGC AG-3′) and 805R (5′-GGA CTA CHV GGG TWT CTA AT-3′) primers, and the V3–V4 PCR amplicons were attached with Illumina indices and adapters from Nextera^Ⓡ^ XT Index Kit (Illumina). PCR amplicons were quantified with a Quant-iTTM PicoGreenTM dsDNA Assay Kit (Thermo). PCR products were purified with a FavorPrepTM DNA gel extraction kit (Favorgen). Quality assessment for confirmation of the DNA integrity and product size was conducted on a Bioanalyzer 2100 instrument (Agilent) using a DNA 7500 chip. Raw reads were processed starting with quality check and filtering of low quality (< Q25) reads. Taxonomic assignment was conducted based on the EzBioCloud database at the species level with a 97% similarity cutoff. Normalization was performed the counts of individual OTUs in a sample by dividing the total counts of all OTUs within that sample followed by a multiplication by resulting in relative abundance (RA) expressed. The centered log-ratio transformation (CLR) was used to handle compositional data as previously reported [98]. The relative abundances were converted to log-ratios of given taxa. For dimensionality reduction techniques requiring a log transform, a pseudo-count of 1 read was added to all taxa. Alpha-proteobacteria *Acinetobacter* spp. were used as reference frames for class and genus, respectively, based on consistent abundance across different experimental conditions.

### Calculation of bacteria diversity

For the calculation of alpha diversity, abundance-based coverage estimators (ACE) [99], Chao estimator [100], Shannon index [101], and Inverse Simpson index [102], as well as construction of a distance matrix and clustering, were conducted using the QIIME pipeline [103]. For principal coordinate analysis (PCoA), representative sequences were extracted from each sample and beta diversity was estimated by calculating weighed UniFrac distances [104] in QIIME.

### RNA extraction and RNA-seq analysis

GITs of zebrafish larvae at 7 dpf were dissected using fine pins and forceps manually and 25 larvae GITs per group pooled. We performed biological duplication for each group. Total RNA was isolated using Trizol reagent (Invitrogen) and RNeasy mini kit (QIAGEN). RNA quality was assessed by an Agilent 2100 bioanalyzer using RNA 6000 Nano Chip (Agilent). RNA sequencing libraries were prepared using the TruSeq RNA Sample Prep Kit (Illumina), and the sequencing was performed at Macrogen Inc. (Seoul, Korea) using the Illumina HiSeq2000 platform to generate 100 base pair paired-end reads. Trim galore! (v.0.6.5, https://www.bioinformatics.babraham.ac.uk/projects/trim_galore/) was used to trim the low quality read with default parameter, and FastQC (v.0.11.9, https://www.bioinformatics.babraham.ac.uk/projects/fastqc/) was used to perform quality check. The zebrafish reference genomes were obtained from NCBI Genome (https://www.ncbi.nlm.nih.gov/genome/), and genome indexing was performed using STAR (v.2.5.1) [105]. The sequenced reads were mapped to the *Danio rerio* (danRer 10) using STAR. The gene expression levels were quantified using the count module of STAR. The edgeR (v.3.12.1) package was used to select differentially expressed genes from the RNA-seq count data [106]. Following the analysis pipeline provided in the package, setting up the model for each comparison condition, estimating tag wise dispersion, and model fitting were performed, then the fold change and FDR values were obtained using the edgeR “topTags” function. For comparison conditions, up- or downregulated genes were calculated for CR-*tp53*^*e7/e7*^ vs. CR-WT and GF-*tp53*^*e7/e7*^ vs. GF-WT, respectively, and then the genes between the two groups were shown in a Venn diagram (Fig. 5b). Cut off for each comparison condition was set to Fold change > 2.0 and FDR < 0.05. The trimmed mean of *M* values (TMM) normalization method was used to calculate the library size, and the counts per million mapped reads (CPM) for each gene was added to 1 and log2-transformed for further analysis. To double-confirm the mutation of *tp53*, *tp53* M214K loci in RNA sequencing of WT, and *tp53* mutant GITs under CR or GF conditions were identified. All groups of *tp53* mutants in CR or GF condition were confirmed to harbor T to A transversion at the *tp53* M214K loci (Additional file 1: Fig. S6a).

### Gene set analysis of the zebrafish differently expressed genes

To functionally annotate the differently expressed genes among the samples, KEGG pathway analyses were performed using PAGE [107, 108]. Briefly, Z-score for each gene set was calculated as *Z* = (Sm–μ)*m^1/2^ / δ. The mean of fold change values of genes for a given gene set was Sm and the size of a given gene set was m. mean of total fold change values (μ) and standard deviation of total fold change values (δ) of a given data set were calculated from input data containing fold change values for each genes between two experimental groups.

### Quantitation of sialic acids by high-performance liquid chromatography

Dissected 25 larvae GITs of WT and *tp53* mutants at 7 dpf were resuspended in PBS and homogenized with a sonication. Major sialic acids in zebrafish GITs were Neu5Ac and Neu5Gc but not Kdn (Additional file 1: Fig. S11a). To quantify free sialic acids, homogenate was precipitated using ethanol to a final concentration of 80% and kept for 1 h at − 40 °C, and then centrifuged at 16,000×*g* for 20 min. The supernatant was collected and purified by solid phase extraction (SPE) with a graphitized carbon (PGC) cartridge as previously described [109]. To quantify the conjugated sialic acid, the pellet was treated with mild hydrolysis in 0.5 M formic acid at 90 °C for 30 min and then purified by PGC-SPE. Sialic acids bound on tissue were directly released by mild hydrolysis in 0.5 M formic acid at 90 °C for 30 min and then purified by solid phase extraction with a graphitized carbon (PGC) cartridge. For 1, 2-diamino-4, 5-methyleneoxybenzene (DMB, Sigma-Aldrich) derivatization, purified sialic acids were reacted with DMB reagent (8 mM DMB, 0.25 M sodium hydrosulfite, 0.8 mM 2-mercaptoethanol, and 1.5 M acetic acid) at 60 °C for 5 h. The DMB-derivatized samples were separated on a C18 reverse phase high-performance liquid chromatography (HPLC) column (2.1 × 50 mm, 1.8 μm, Agilent) by a binary gradient solvent A, 100% H_2_O and B, 100% methanol on an Agilent 1290 Infinity II LC System. The 12-min gradient was used at 0.5 μL/min of flow rate with the following solvent proportions and time points: 0–1 min, 0–15%, B; 1–10 min, 15–20%, B; 10–10.5 min, 20–95%, B; 10.5–12 min, 95%, B; 12–12.1 min, 95–15%, B. Sialic acids including *N*-acetyl neuraminic acid (NeuAc) and *N*-Glycolyl neuraminic acid (NeuGc) were detected at 448 nm using excitation at 373 nm on a fluorescence detector. They were identified by referring to the elution time of standard Neu5Ac and Neu5Gc derivatives. Individual sialic acid derivatives were quantified by integration of fluorescence intensity after HPLC separation, plotted against standard curves of corresponding authentic standards. Total sialic acids were the calculated sum of free and bounded sialic acids.

### Statistics

The number of zebrafish larvae per group is annotated in corresponding figure legends. The raw data of each group were dotted in figures. If the distribution was normal and the variances were equal, statistical analyses of the data were performed using one-way ANOVA with Newman-Keuls multiple comparisons test or a student’s *t* test. If data were not normally distributed, non-parametric Friedman tests with Dunn’s multiple comparisons test or Mann-Whitney *U* test were used. Comparison of lethality curves was performed using the Gehan–Breslow–Wilcoxon tests. All statistical analyses were performed using Prism 6 software (GraphPad). In all figures, data are represented as mean ± SEM. *p* values correlate with symbols as follows: *NS* = not significant, *p ≤ 0.05, **p ≤ 0.01, ***p ≤ 0.005, and ****p ≤ 0.001.

## Supplementary Information

**Additional file 1: Supplementary methods and Supplementary Figures. Figure S1.** *tp53* mutant GITs are aberrantly infiltrated by increased numbers of neutrophils and exhibit hyperimmune responses similar to those induced by DSS treatment. **Figure S2.** Overview of the experimental procedure and comparisons of gross anatomy between the wild type and *tp 53* mutants. **Figure S3.** The increased number of Alcian blue-positive goblet cells in *tp53* mutants are due to Gram (-) bacteria. **Figure S4.** Gamma-proteobacteria class is enriched in the GITs of *tp53* mutants. **Figure S5.** *Aeromonas* spp., *Citrobacter* spp., and *Pseudomonas* spp. are enriched in the *tp53* mutant GITs. **Figure S6.** *E. coli* does not induce the increase of Alcian blue-positive goblet cells. **Figure S7.** *Photobacterium damselae* DreWT1 isolated from GITs of WT does not induce the increase of NFκB-EGFP activity. **Figure S8.** Endogenous *Aeromonas* spp. as well as *Pseudomonas* spp. and *Citrobacter* spp. were isolated from *tp53* mutants in AMB agar plate culture. **Figure S9.** *tp53* mutation alters metabolic pathways in GITs. **Figure S10.** SCFA levels show no differences between WT and *tp53* mutants. **Figure S11.** Oseltamivir treatment does not alter free sialic acid levels of the host. **Figure S12.** Neu5Gc, but not Neu5Ac is utilized as a carbon source by *Aeromonas jandaei* TP531 for its growth. **Figure S13.** Elevated inflammation elicited by exogenous addition of *mCherry*-tagged *A. jandaei* TP531 in *tp53* mutants is abolished by limiting available sialic acids with oseltamivir. **Figure S14.** Neu5Gc supplementation elevates intestinal inflammation but does barely promote *Aeromonas* blooming. **Figure S15.** Neu5Ac supplementation elevates intestinal inflammation in a microbiota-dependent manner, but does not promote blooming of *Aeromonas* spp.. **Figure S16.** *Citrobacter* spp. may outcompete *Aeronomas* spp. when Neu5Gc and Neu5Ac are supplemented as carbon sources. **Figure S17.** Monoassociation with *A. jandaei* TP531 does not induce the lethality and the increase of Alcian blue-positive goblet cells. **Figure S18.** A proposed working model illustrates dysbiosis and intestinal inflammation by *tp53* mutation via imbalanced sialometabolism and a potential therapeutic intervention.

**Additional file 2.** Lists of differentially expressed genes (DEGs) of the gastrointestinal tracts (GITs) from WT vs. *tp53* mutants or CR vs. GF conditions at 7 dpf.

## Data Availability

NGS data has been deposited in the NCBI Gene Expression Omnibus (https://www.ncbi.nlm.nih.gov/geo/) under accession number GSE150376.

## Abbreviations

AMB: *Aeromonas* medium base
CAC: Colorectal cancer
CFU: Colony forming unit
CR: Conventionally raised
DEG: Differentially expressed gene
dpf: Days post fertilization
DSS: Dextran sulfate sodium
GF: Germfree
GIT: Gastrointestinal tract
OTU: Operational taxonomic unit
OV: Oseltamivir
SA: Sialic acid
STn: Sialyl-Thomsen-nouveau
WT: Wild-type
