# Supplementary material for: Host tp53 mutation induces gut dysbiosis eliciting inflammation through disturbed sialic acid metabolism
Source: Microbiome. 2022 Jan 6;10:3. doi: 10.1186/s40168-021-01191-x (PMC8733924; doi:10.1186/s40168-021-01191-x)
Supplement: Supplementary file 2 — Additional file 1: Supplementary methods and Supplementary Figures. Figure S1. tp53 mutant GITs are aberrantly infiltrated by increased numbers of neutrophils and exhibit hyperimmune responses similar to those induced by DSS treatment. Figure S2. Overview of the experimental procedure and comparisons of gross anatomy between the wild type and tp 53 mutants. Figure S3. The increased number of Alcian blue-positive goblet cells in tp53 mutants are due to Gram (-) bacteria. Figure S4. Gamma-proteobacteria class is enriched in the GITs of tp53 mutants. Figure S5. Aeromonas spp., Citrobacter spp., and Pseudomonas spp. are enriched in the tp53 mutant GITs. Figure S6. E. coli does not induce the increase of Alcian blue-positive goblet cells. Figure S7. Photobacterium damselae DreWT1 isolated from GITs of WT does not induce the increase of NFκB-EGFP activity. Figure S8. Endogenous Aeromonas spp. as well as Pseudomonas spp. and Citrobacter spp. were isolated from tp53 mutants in AMB agar plate culture. Figure S9. tp53 mutation alters metabolic pathways in GITs. Figure S10. SCFA levels show no differences between WT and tp53 mutants. Figure S11. Oseltamivir treatment does not alter free sialic acid levels of the host. Figure S12. Neu5Gc, but not Neu5Ac is utilized as a carbon source by Aeromonas jandaei TP531 for its growth. Figure S13. Elevated inflammation elicited by exogenous addition of mCherry-tagged A. jandaei TP531 in tp53 mutants is abolished by limiting available sialic acids with oseltamivir. Figure S14. Neu5Gc supplementation elevates intestinal inflammation but does barely promote Aeromonas blooming. Figure S15. Neu5Ac supplementation elevates intestinal inflammation in a microbiota-dependent manner, but does not promote blooming of Aeromonas spp.. Figure S16. Citrobacter spp. may outcompete Aeronomas spp. when Neu5Gc and Neu5Ac are supplemented as carbon sources. Figure S17. Monoassociation with A. jandaei TP531 does not induce the lethality and the increase of A [file 40168_2021_1191_MOESM2_ESM.zip › 3. Suppl Figures_final ver.pdf]

Figure S1.

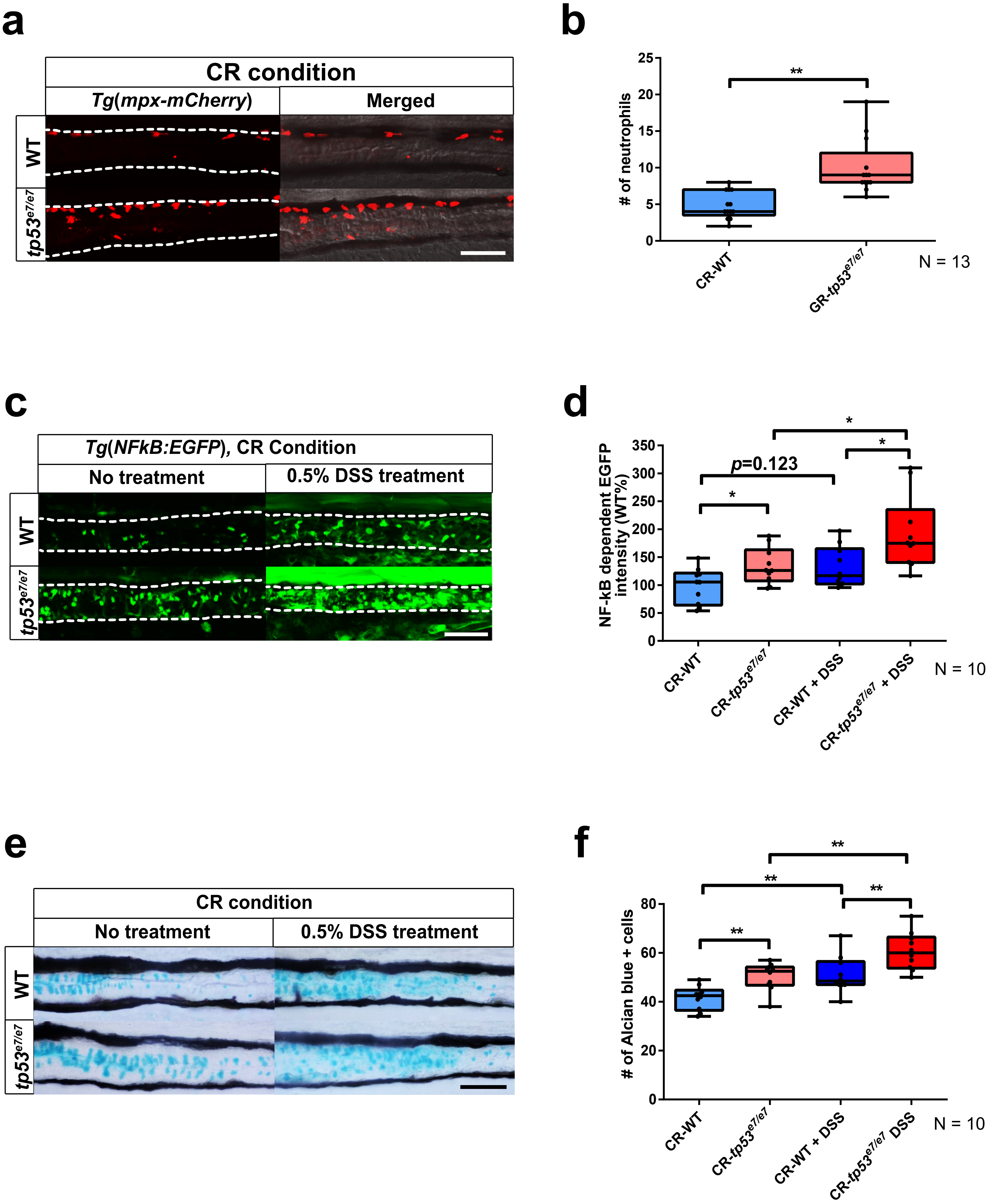
a
CR condition
Tg(mpx-mCherry)
Merged
WT
tp53e7/e7
b
# of neutrophils
**
CR-WT
GR-tp53e7/e7
N = 13
c
Tg(NFkB:EGFP), CR Condition
No treatment
0.5% DSS treatment
WT
tp53e7/e7
d
NF-kB dependent EGFP intensity (WT%)
p=0.123
CR-WT
CR-tp53e7/e7
CR-WT + DSS
CR-tp53e7/e7 + DSS
N = 10
e
CR condition
No treatment
0.5% DSS treatment
WT
tp53e7/e7
f
# of Alcian blue + cells
**
CR-WT
CR-tp53e7/e7
CR-WT + DSS
CR-tp53e7/e7 DSS
N = 10

Figure S2.

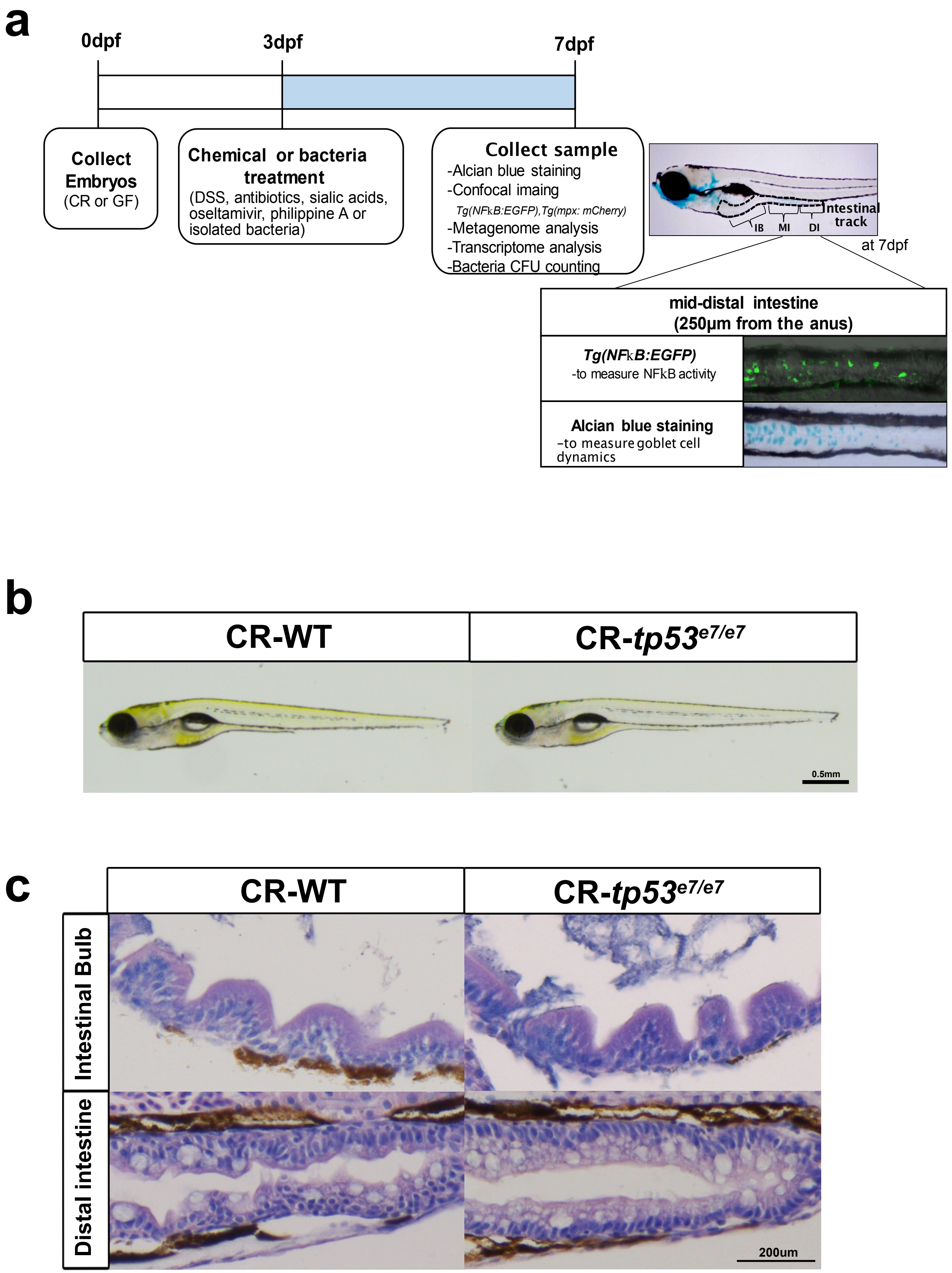

a
0dpf
3dpf
7dpf
Collect Embryos
(CR or GF)
Chemical or bacteria treatment
(DSS, antibiotics, sialic acids, oseltamivir, philippine A or isolated bacteria)
Collect sample
-Alcian blue staining
-Confocal imaing
Tg(NFkB:EGFP),Tg(mpx: mCherry)
-Metagenome analysis
-Transcriptome analysis
-Bacteria CFU counting
IB
MI
DI
Intestinal track
at 7dpf
mid-distal intestine
(250µm from the anus)
Tg(NFkB:EGFP)
-to measure NFkB activity
Alcian blue staining
-to measure goblet cell dynamics
b
CR-WT
CR-tp53e7/e7
0.5mm
c
CR-WT
CR-tp53e7/e7
Intestinal Bulb
Distal intestine
200um

Figure S3.

a

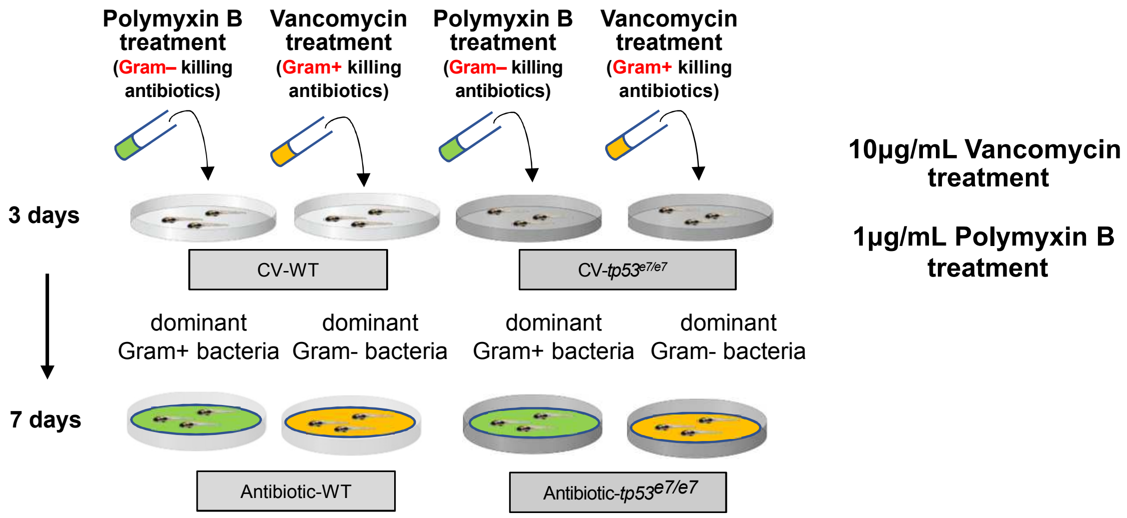

b

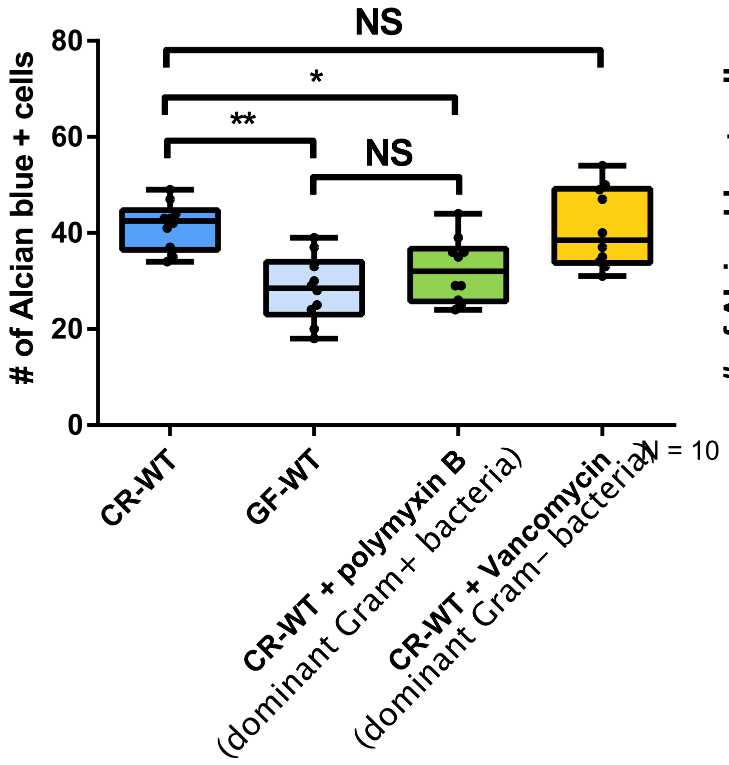

c

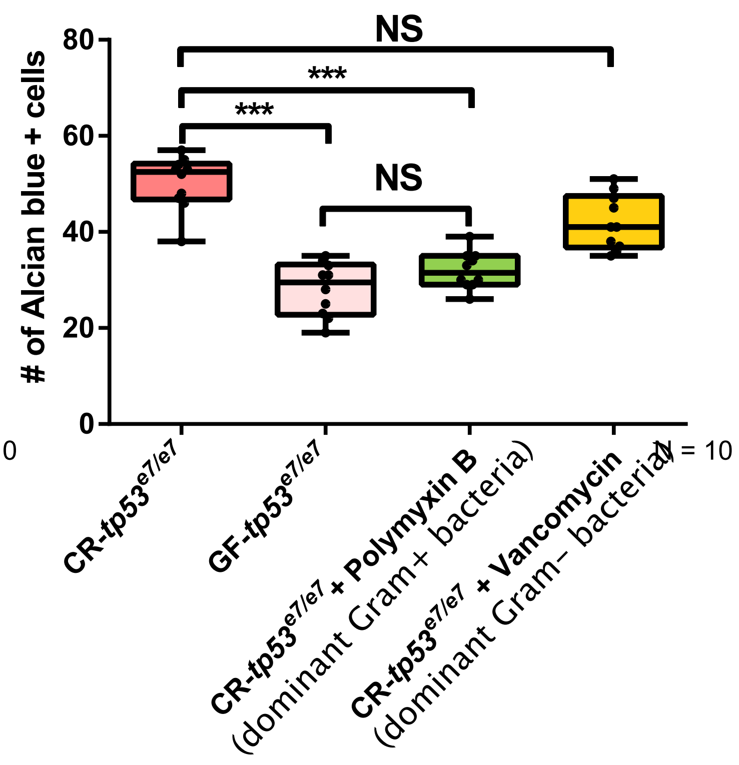

Figure S4.

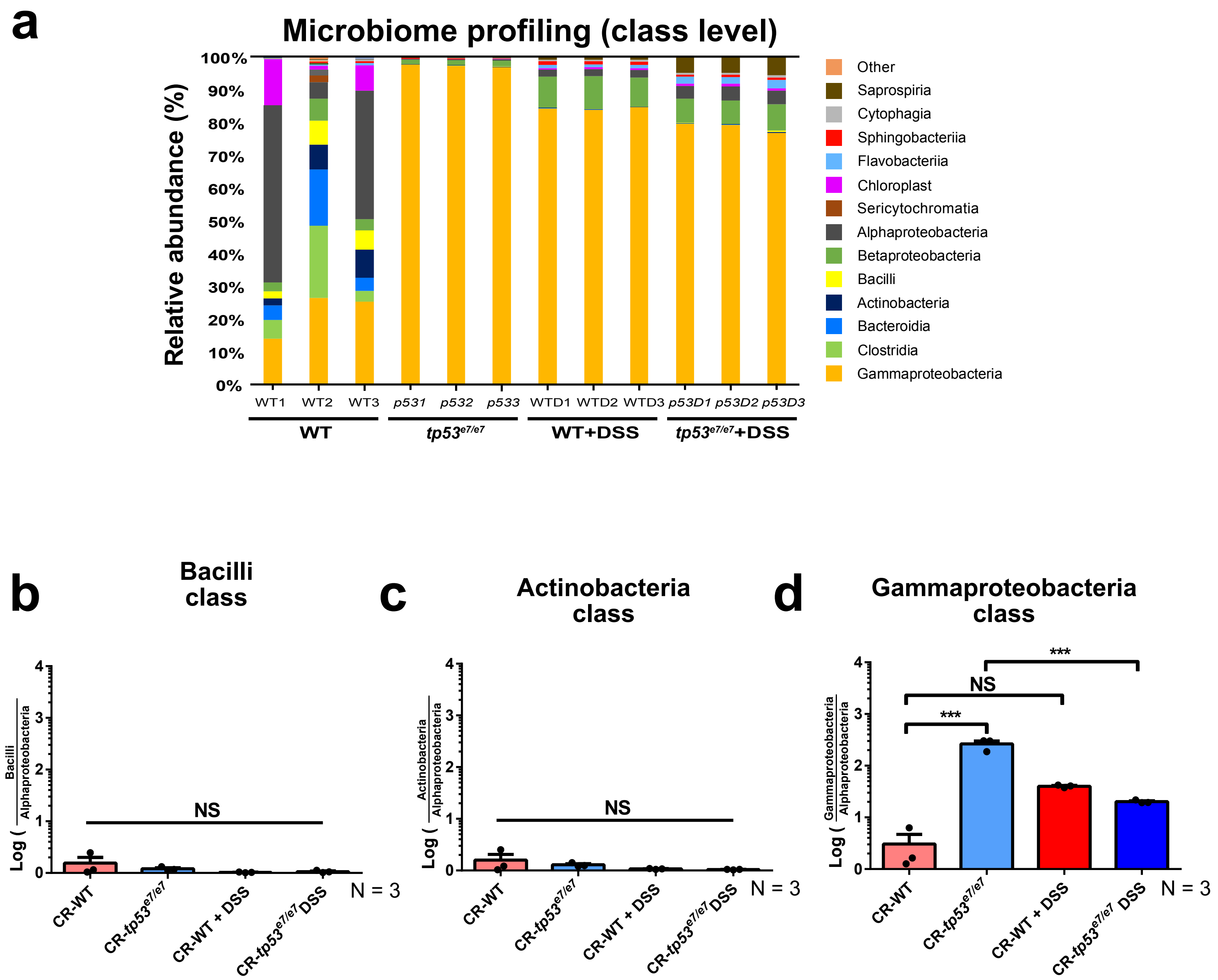

a
Microbiome profiling (class level)
Relative abundance (%)
100%
90%
80%
70%
60%
50%
40%
30%
20%
10%
0%
WT1 WT2 WT3 p531 p532 p533 WTD1 WTD2 WTD3 p53D1 p53D2 p53D3
WT
tp53e7/e7
WT+DSS
tp53e7/e7+DSS
Other
Saprospiria
Cytophagia
Sphingobacteriia
Flavobacteriia
Chloroplast
Sericytochromatia
Alphaproteobacteria
Betaproteobacteria
Bacilli
Actinobacteria
Bacteroidia
Clostridia
Gammaproteobacteria
b
Bacilli class
Log (Bacilli / Alphaproteobacteria)
NS
CR-WT
CR-tp53e7/e7
CR-WT + DSS
CR-tp53e7/e7 DSS
N = 3
c
Actinobacteria class
Log (Actinobacteria / Alphaproteobacteria)
NS
CR-WT
CR-tp53e7/e7
CR-WT + DSS
CR-tp53e7/e7 DSS
N = 3
d
Gammaproteobacteria class
Log (Gammaproteobacteria / Alphaproteobacteria)
***
NS
***
CR-WT
CR-tp53e7/e7
CR-WT + DSS
CR-tp53e7/e7 DSS
N = 3

Figure S5.

a

OTU	NCBI	Read number (normalized)				ID (%)
	Cut off ($p<0.05$, FC>2)	WT	WT + DSS	*tp53*$^{e7/e7}$	*tp53*$^{e7/e7}$ +DSS	
0	*Aeromonas rivipollensis* strain P2G1 16S ribosomal RNA, partial sequence	6512	10260	13513	34316	99%
1	*Pseudomonas guezennei* strain RA26 16S ribosomal RNA, partial sequence	644	4632	18571	1741	99%
4	*Citrobacter freundii* strain LMG 3246 16S ribosomal RNA gene, partial sequence	185	7885	3279	1529	99%
25	*Trichocoleus desertorum* strain ATA4-8-CV2 16S ribosomal RNA gene, partial sequence	1	199	10	513	82%
31	*Pseudomonas entomophila* strain L48 16S ribosomal RNA, partial sequence	172	1286	1475	1114	99%
34	*Undibacterium squillarum* strain CMJ-15 16S ribosomal RNA, partial sequence	0	267	5	241	99%
59	*Pseudomonas savastanoi* strain CFBP 1670 16S ribosomal RNA gene, partial sequence	0	436	63	17	98%
62	*Rhizobium daejeonense* strain NBRC 102495 16S ribosomal RNA gene, partial sequence	0	33	29	133	85%
255	*Bdellovibrio bacteriovorus* strain HD100 16S ribosomal RNA gene, partial sequence	0	18	6	2	93%

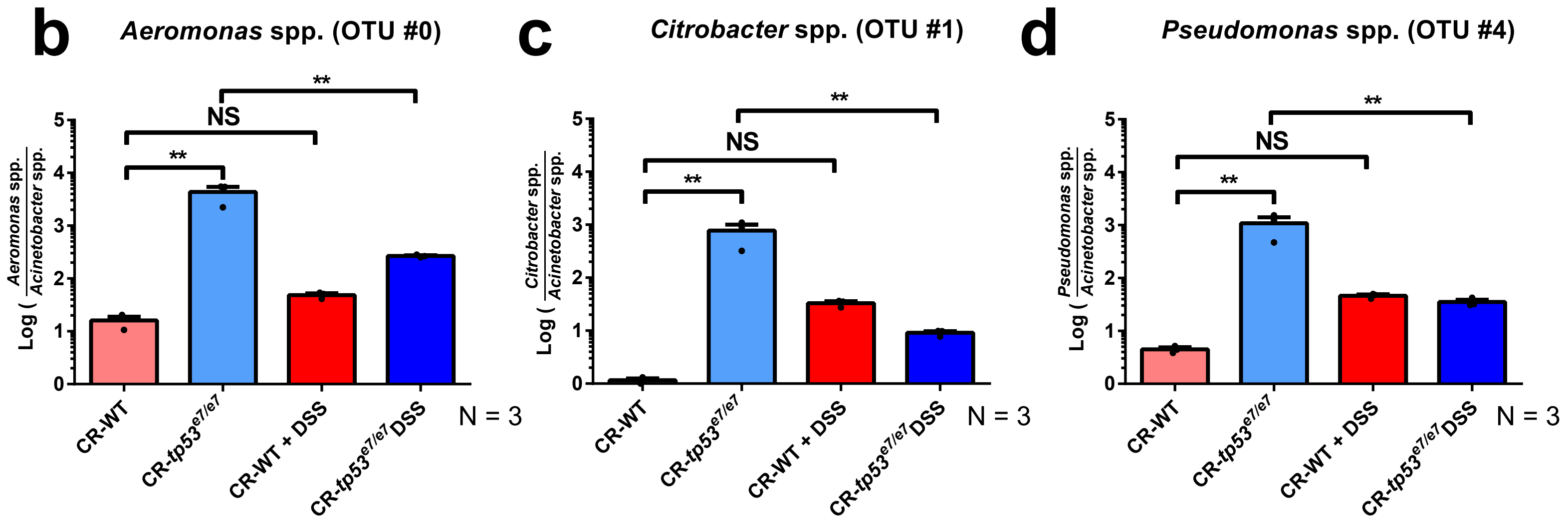

Figure S6.

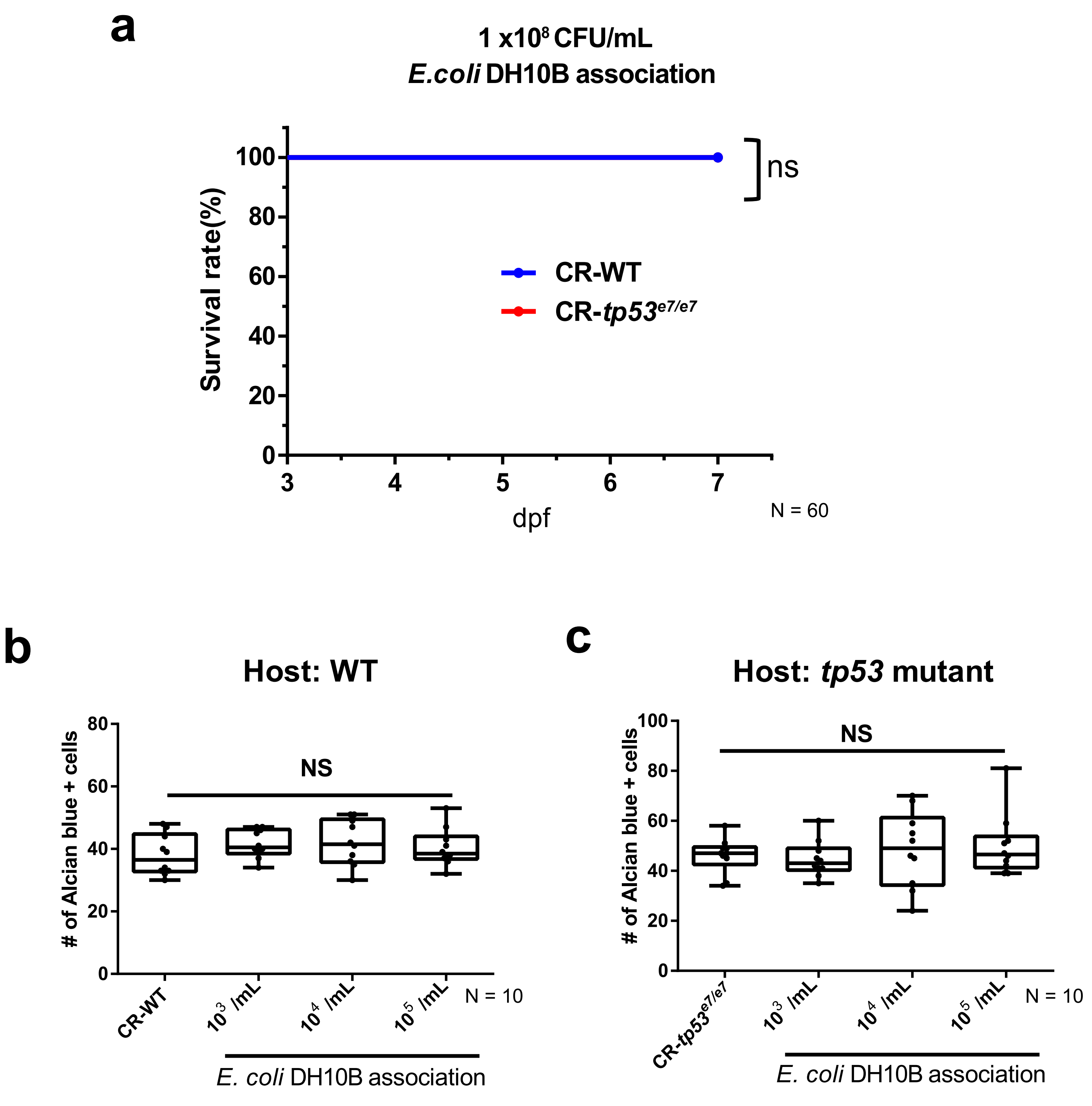
a
1 x10^8 CFU/mL
E.coli DH10B association
Survival rate(%)
100
80
60
40
20
0
3
4
5
6
7
dpf
ns
CR-WT
CR-tp53e7/e7
N = 60
b
Host: WT
# of Alcian blue + cells
80
60
40
20
0
NS
CR-WT
10³ /mL
10⁴ /mL
10⁵ /mL
N = 10
E. coli DH10B association
c
Host: tp53 mutant
# of Alcian blue + cells
100
80
60
40
20
0
NS
CR-tp53e7/e7
10³ /mL
10⁴ /mL
10⁵ /mL
N = 10
E. coli DH10B association

Figure S7.

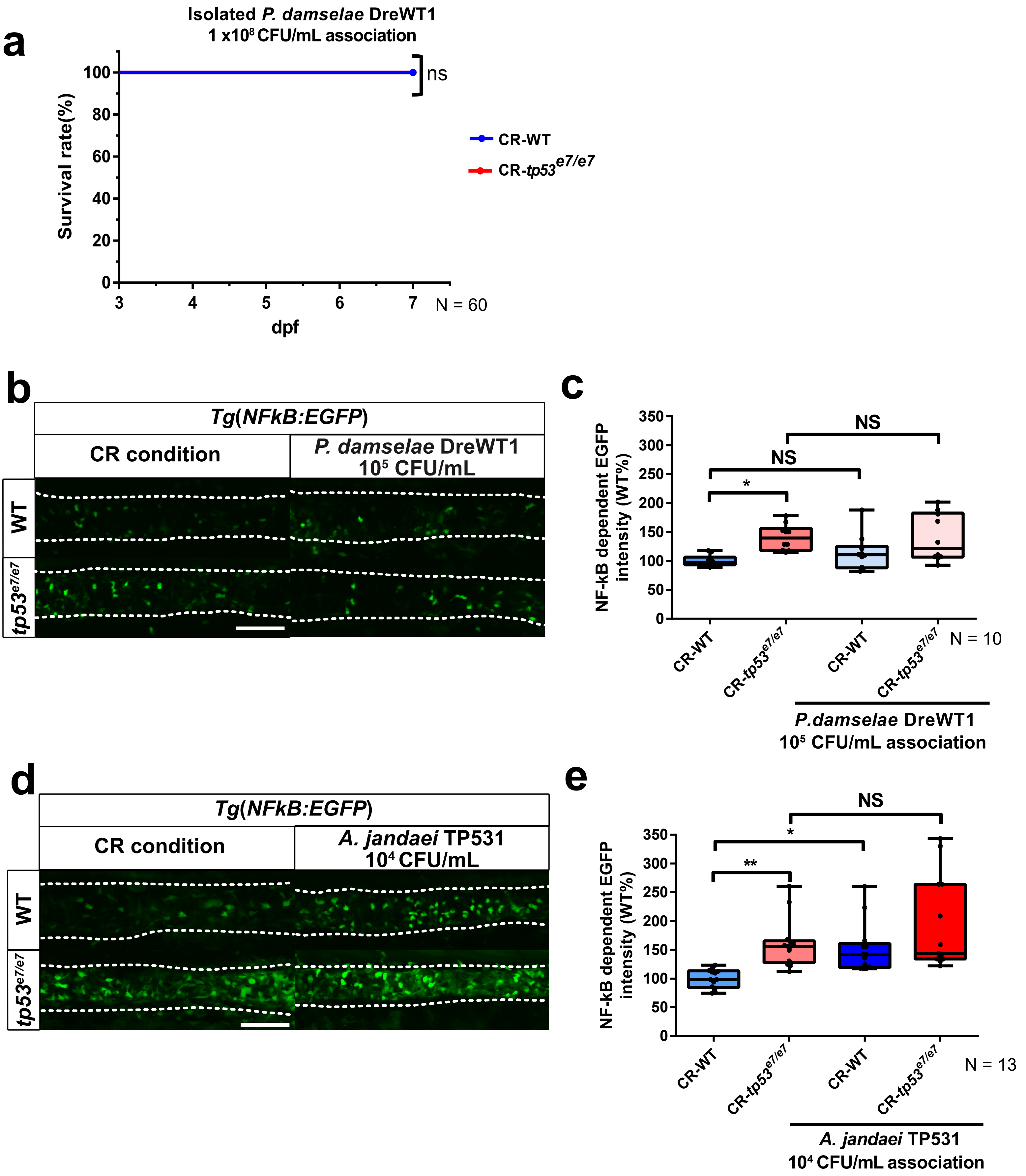
a
Isolated P. damselae DreWT1
1 x10[8] CFU/mL association
Survival rate(%)
100
80
60
40
20
0
ns
3
4
5
6
7
N = 60
dpf
CR-WT
CR-tp53e7/e7
b
Tg(NFkB:EGFP)
CR condition
P. damselae DreWT1
10[5] CFU/mL
WT
tp53e7/e7
c
NF-kB dependent EGFP intensity (WT%)
350
300
250
200
150
100
50
0
NS
NS
*
CR-WT
CR-tp53e7/e7
CR-WT
CR-tp53e7/e7
N = 10
P.damselae DreWT1
10[5] CFU/mL association
d
Tg(NFkB:EGFP)
CR condition
A. jandaei TP531
10[4] CFU/mL
WT
tp53e7/e7
e
NF-kB dependent EGFP intensity (WT%)
350
300
250
200
150
100
50
0
NS
*
**
CR-WT
CR-tp53e7/e7
CR-WT
CR-tp53e7/e7
N = 13
A. jandaei TP531
10[4] CFU/mL association

Figure S8.

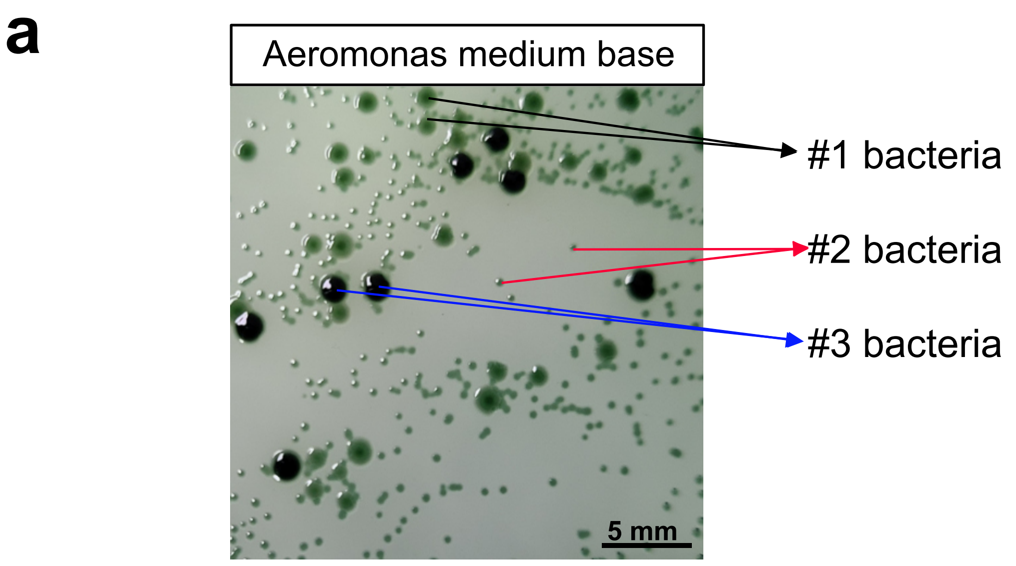
a
Aeromonas medium base
#1 bacteria
#2 bacteria
#3 bacteria
5 mm

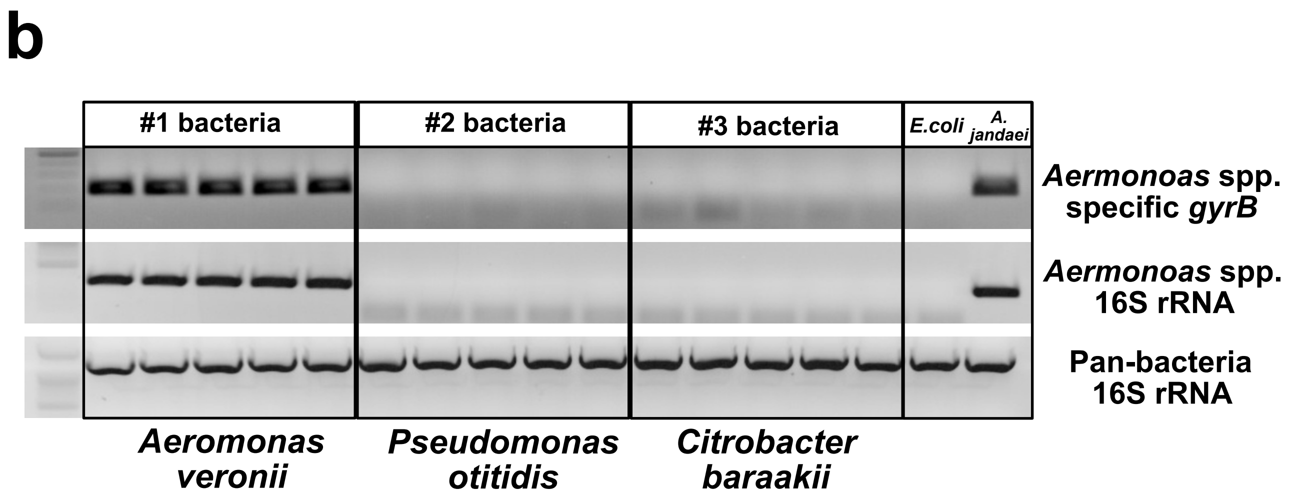
b
#1 bacteria
#2 bacteria
#3 bacteria
E.coli
A. jandaei
Aermonoas spp. specific gyrB
Aermonoas spp. 16S rRNA
Pan-bacteria 16S rRNA
Aeromonas veronii
Pseudomonas otitidis
Citrobacter baraakii

Figure S9.

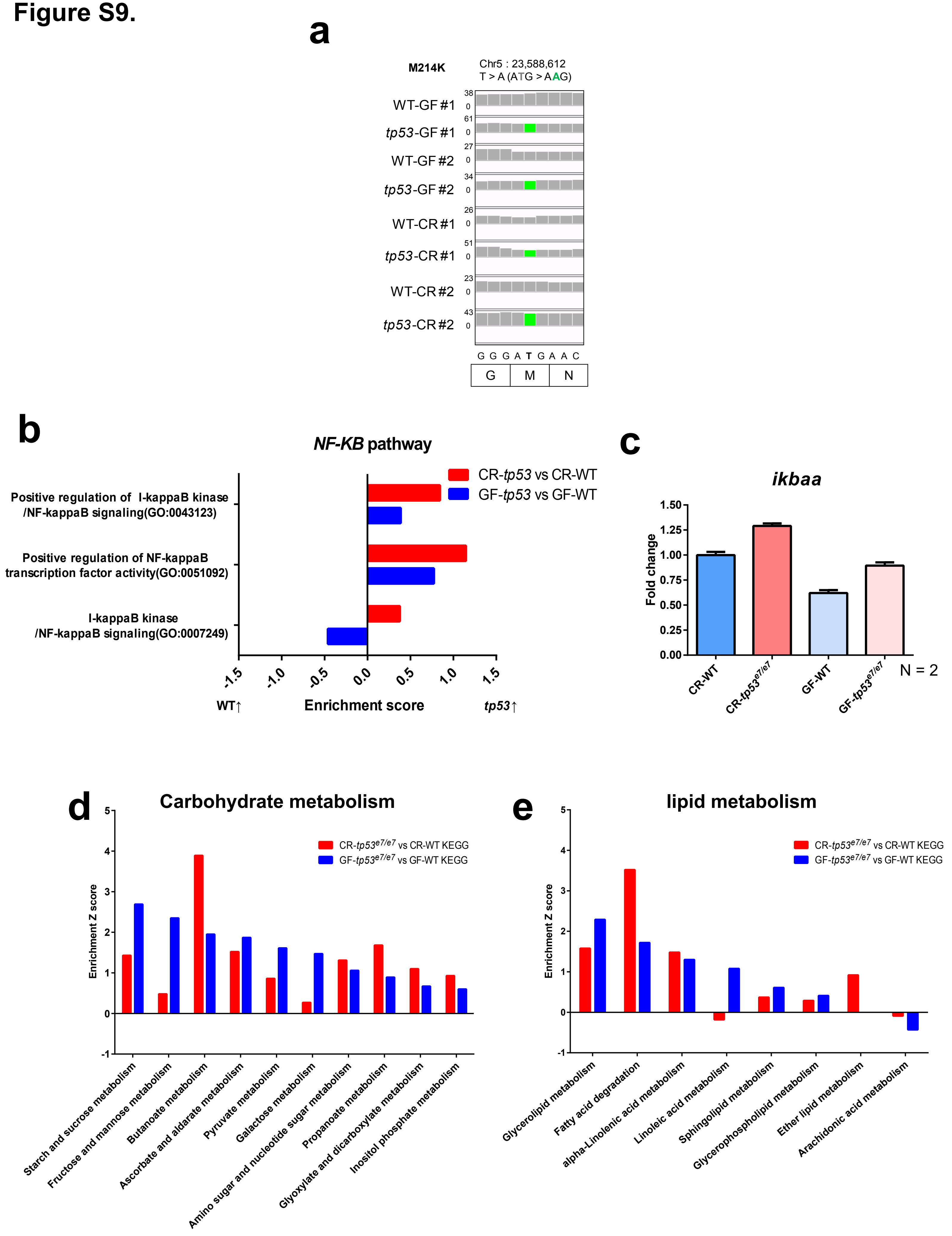
a
M214K
Chr5 : 23,588,612
T > A (ATG > AAG)
WT-GF #1
tp53-GF #1
WT-GF #2
tp53-GF #2
WT-CR #1
tp53-CR #1
WT-CR #2
tp53-CR #2
G G G A T G A A C
G M N
b
NF-KB pathway
CR-tp53 vs CR-WT
GF-tp53 vs GF-WT
Positive regulation of I-kappaB kinase /NF-kappaB signaling(GO:0043123)
Positive regulation of NF-kappaB transcription factor activity(GO:0051092)
I-kappaB kinase /NF-kappaB signaling(GO:0007249)
WT↑
Enrichment score
tp53↑
c
ikbaa
Fold change
CR-WT
CR-tp53e7/e7
GF-WT
GF-tp53e7/e7
N = 2
d
Carbohydrate metabolism
CR-tp53e7/e7 vs CR-WT KEGG
GF-tp53e7/e7 vs GF-WT KEGG
Enrichment Z score
Starch and sucrose metabolism
Fructose and mannose metabolism
Butanoate metabolism
Ascorbate and aldarate metabolism
Pyruvate metabolism
Galactose metabolism
Amino sugar and nucleotide sugar metabolism
Propanoate metabolism
Glyoxylate and dicarboxylate metabolism
Inositol phosphate metabolism
e
lipid metabolism
CR-tp53e7/e7 vs CR-WT KEGG
GF-tp53e7/e7 vs GF-WT KEGG
Enrichment Z score
Glycerolipid metabolism
Fatty acid degradation
alpha-Linolenic acid metabolism
Linoleic acid metabolism
Sphingolipid metabolism
Glycerophospholipid metabolism
Ether lipid metabolism
Arachidonic acid metabolism

Figure S10.

a

b

c

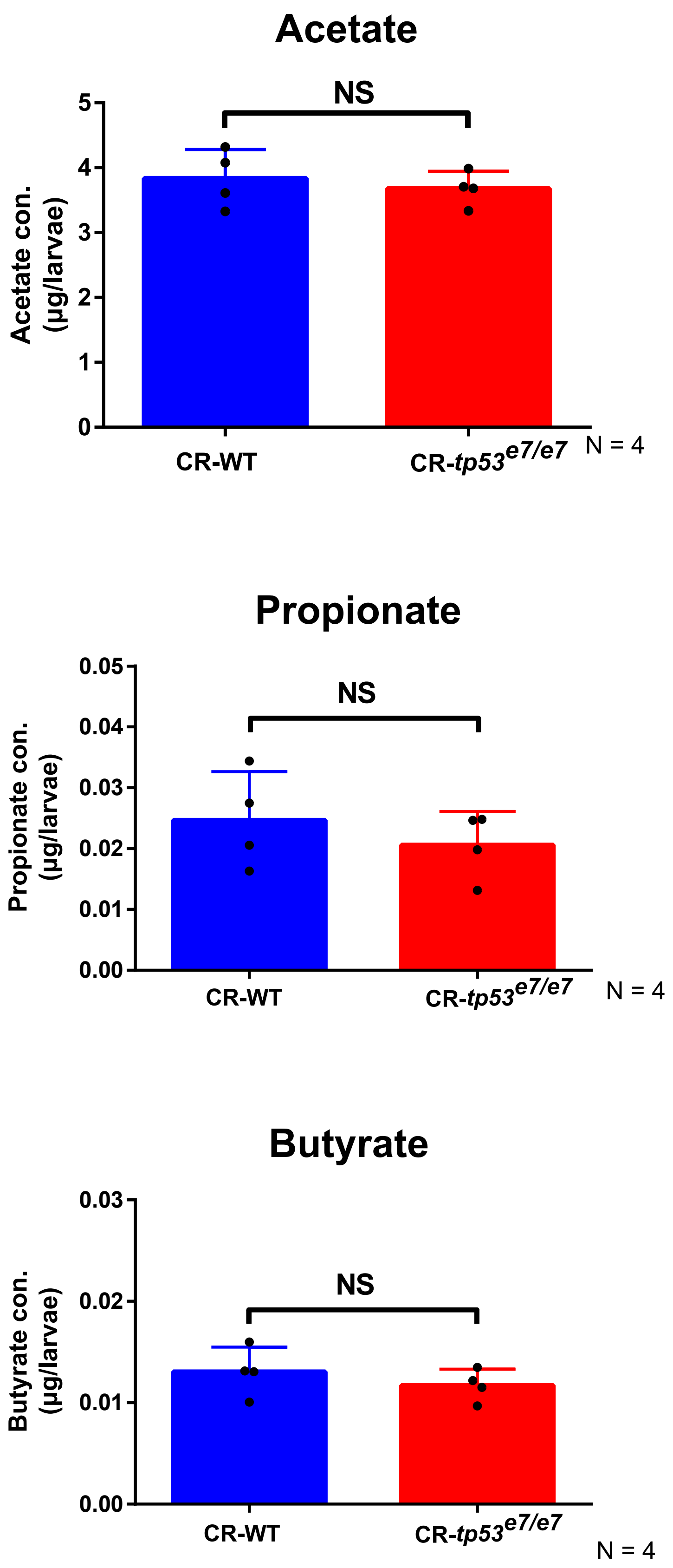

Figure S11.

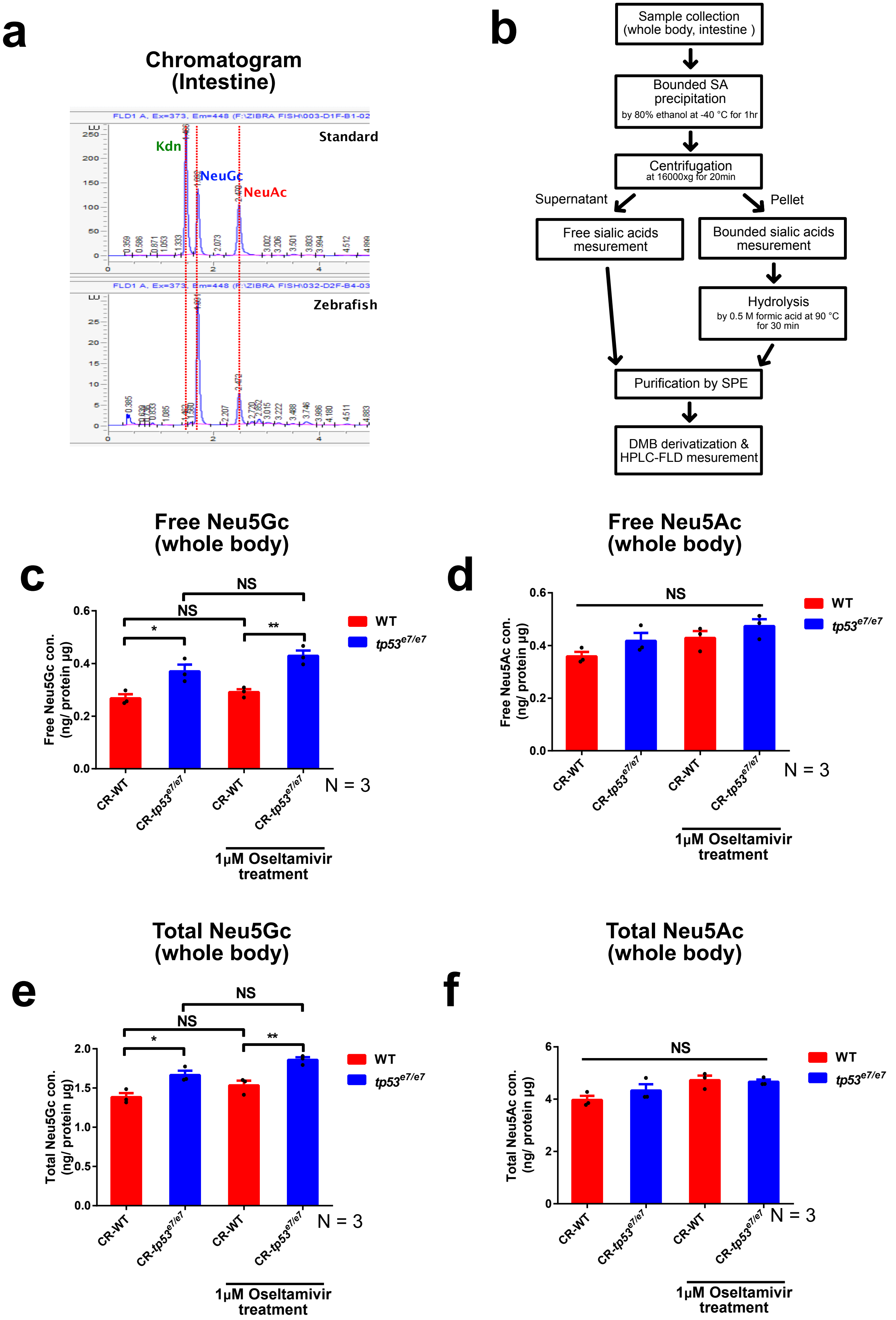
a
Chromatogram
(Intestine)
Standard
Kdn
NeuGc
NeuAc
Zebrafish
b
Sample collection
(whole body, intestine)
Bounded SA
precipitation
by 80% ethanol at -40 °C for 1hr
Centrifugation
at 16000xg for 20min
Supernatant
Pellet
Free sialic acids
mesurement
Bounded sialic acids
mesurement
Hydrolysis
by 0.5 M formic acid at 90 °C
for 30 min
Purification by SPE
DMB derivatization &
HPLC-FLD mesurement
c
Free Neu5Gc
(whole body)
Free Neu5Gc con.
(ng/ protein μg)
NS
NS
*
**
WT
tp53e7/e7
CR-WT
CR-tp53e7/e7
CR-WT
CR-tp53e7/e7
N = 3
1μM Oseltamivir
treatment
d
Free Neu5Ac
(whole body)
Free Neu5Ac con.
(ng/ protein μg)
NS
WT
tp53e7/e7
CR-WT
CR-tp53e7/e7
CR-WT
CR-tp53e7/e7
N = 3
1μM Oseltamivir
treatment
e
Total Neu5Gc
(whole body)
Total Neu5Gc con.
(ng/ protein μg)
NS
NS
*
**
WT
tp53e7/e7
CR-WT
CR-tp53e7/e7
CR-WT
CR-tp53e7/e7
N = 3
1μM Oseltamivir
treatment
f
Total Neu5Ac
(whole body)
Total Neu5Ac con.
(ng/ protein μg)
NS
WT
tp53e7/e7
CR-WT
CR-tp53e7/e7
CR-WT
CR-tp53e7/e7
N = 3
1μM Oseltamivir
treatment

Figure S12.

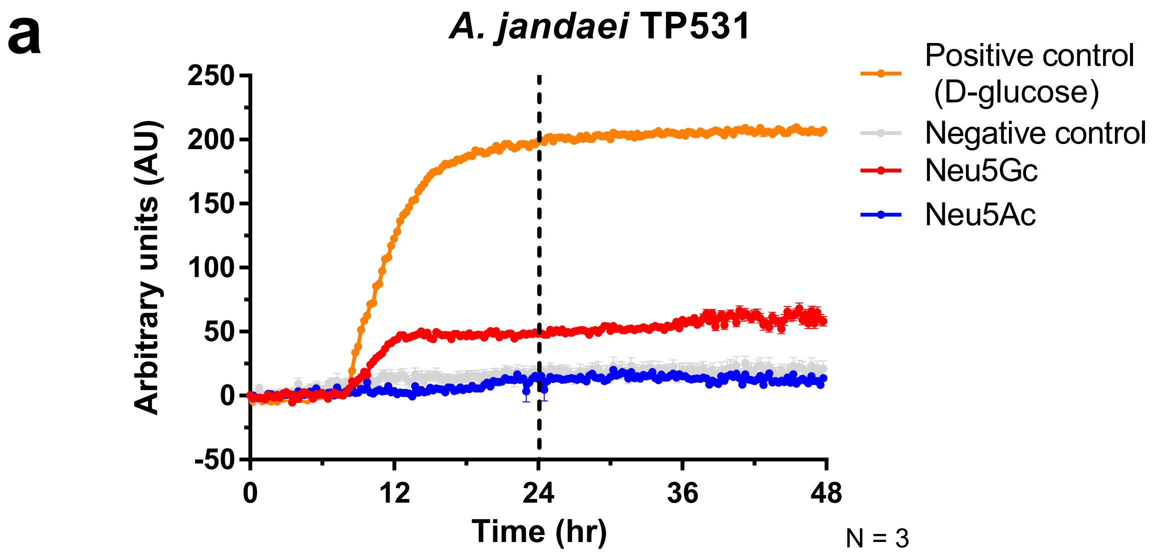

a
A. jandaei TP531
Arbitrary units (AU)
250
200
150
100
50
0
-50
0
12
24
36
48
Time (hr)
N = 3
Positive control (D-glucose)
Negative control
Neu5Gc
Neu5Ac

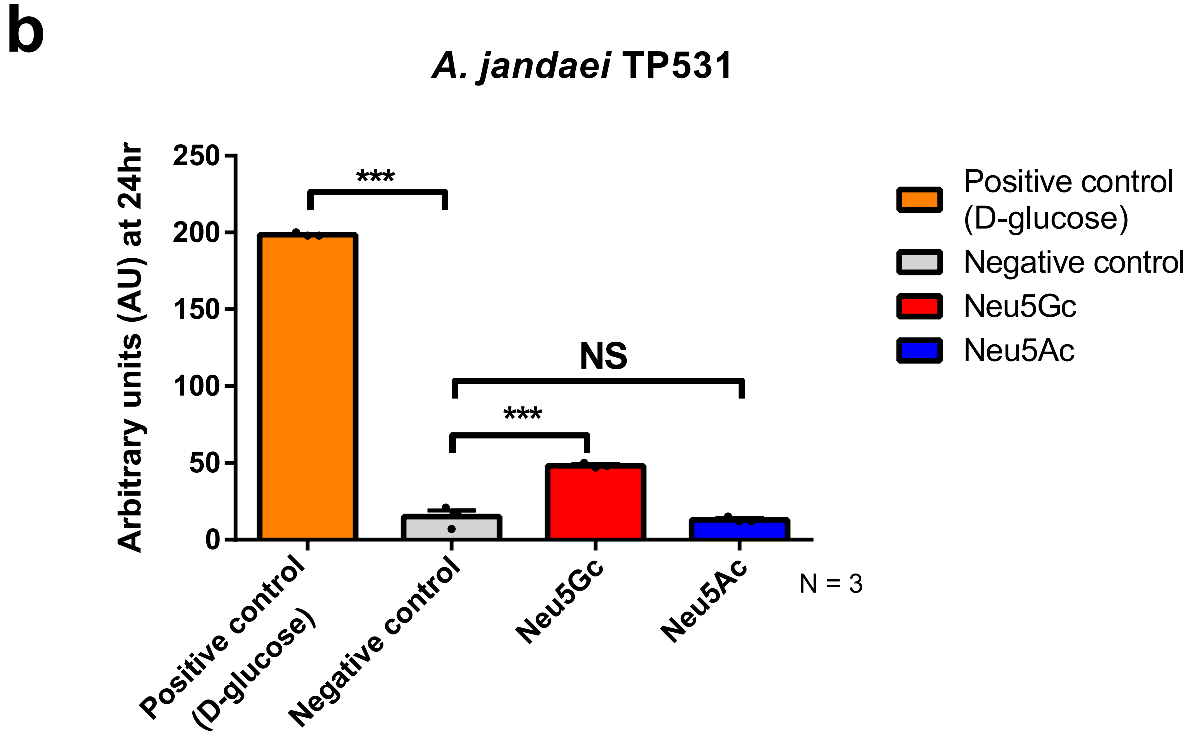

b
A. jandaei TP531
Arbitrary units (AU) at 24hr
250
200
150
100
50
0
***
NS
***
Positive control (D-glucose)
Negative control
Neu5Gc
Neu5Ac
N = 3
Positive control (D-glucose)
Negative control
Neu5Gc
Neu5Ac

Figure S13.

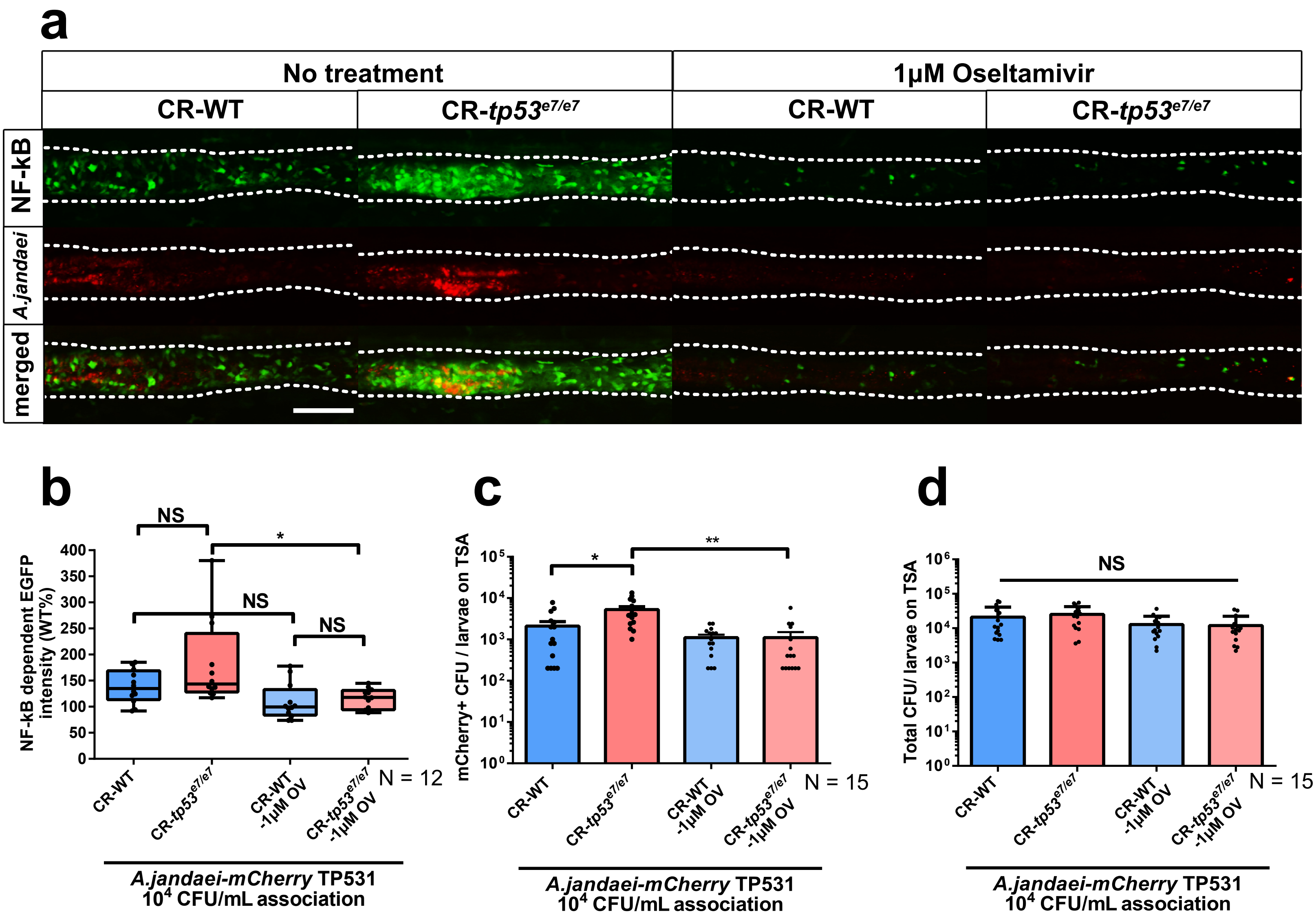
a
No treatment
1μM Oseltamivir
CR-WT
CR-tp53e7/e7
CR-WT
CR-tp53e7/e7
NF-kB
A.jandaei
merged
b
NS
*
NS
NS
NF-kB dependent EGFP intensity (WT%)
0
50
100
150
200
250
300
350
400
CR-WT
CR-tp53e7/e7
CR-WT -1μM OV
CR-tp53e7/e7 -1μM OV
N = 12
A.jandaei-mCherry TP531
10^4 CFU/mL association
c
*
**
mCherry+ CFU / larvae on TSA
10^0
10^1
10^2
10^3
10^4
10^5
CR-WT
CR-tp53e7/e7
CR-WT -1μM OV
CR-tp53e7/e7 -1μM OV
N = 15
A.jandaei-mCherry TP531
10^4 CFU/mL association
d
NS
Total CFU/ larvae on TSA
10^0
10^1
10^2
10^3
10^4
10^5
10^6
CR-WT
CR-tp53e7/e7
CR-WT -1μM OV
CR-tp53e7/e7 -1μM OV
N = 15
A.jandaei-mCherry TP531
10^4 CFU/mL association

Figure S14.

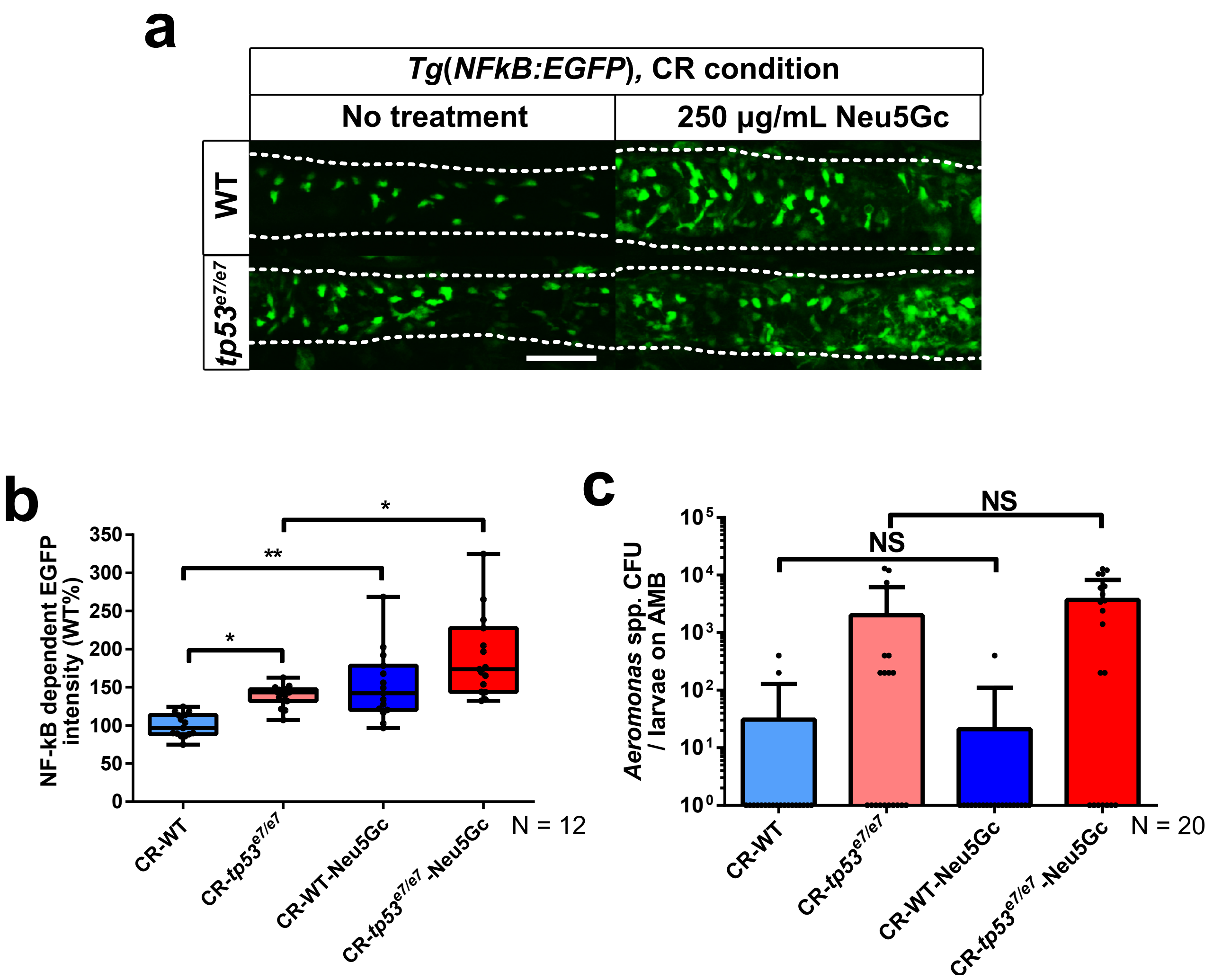

a
Tg(NFkB:EGFP), CR condition
No treatment
250 μg/mL Neu5Gc
WT
tp53e7/e7
b
NF-kB dependent EGFP intensity (WT%)
350
300
250
200
150
100
50
0
*
**
*
CR-WT
CR-tp53e7/e7
CR-WT-Neu5Gc
CR-tp53e7/e7 -Neu5Gc
N = 12
c
Aeromonas spp. CFU / larvae on AMB
10^5
10^4
10^3
10^2
10^1
10^0
NS
NS
CR-WT
CR-tp53e7/e7
CR-WT-Neu5Gc
CR-tp53e7/e7 -Neu5Gc
N = 20

Figure S15.

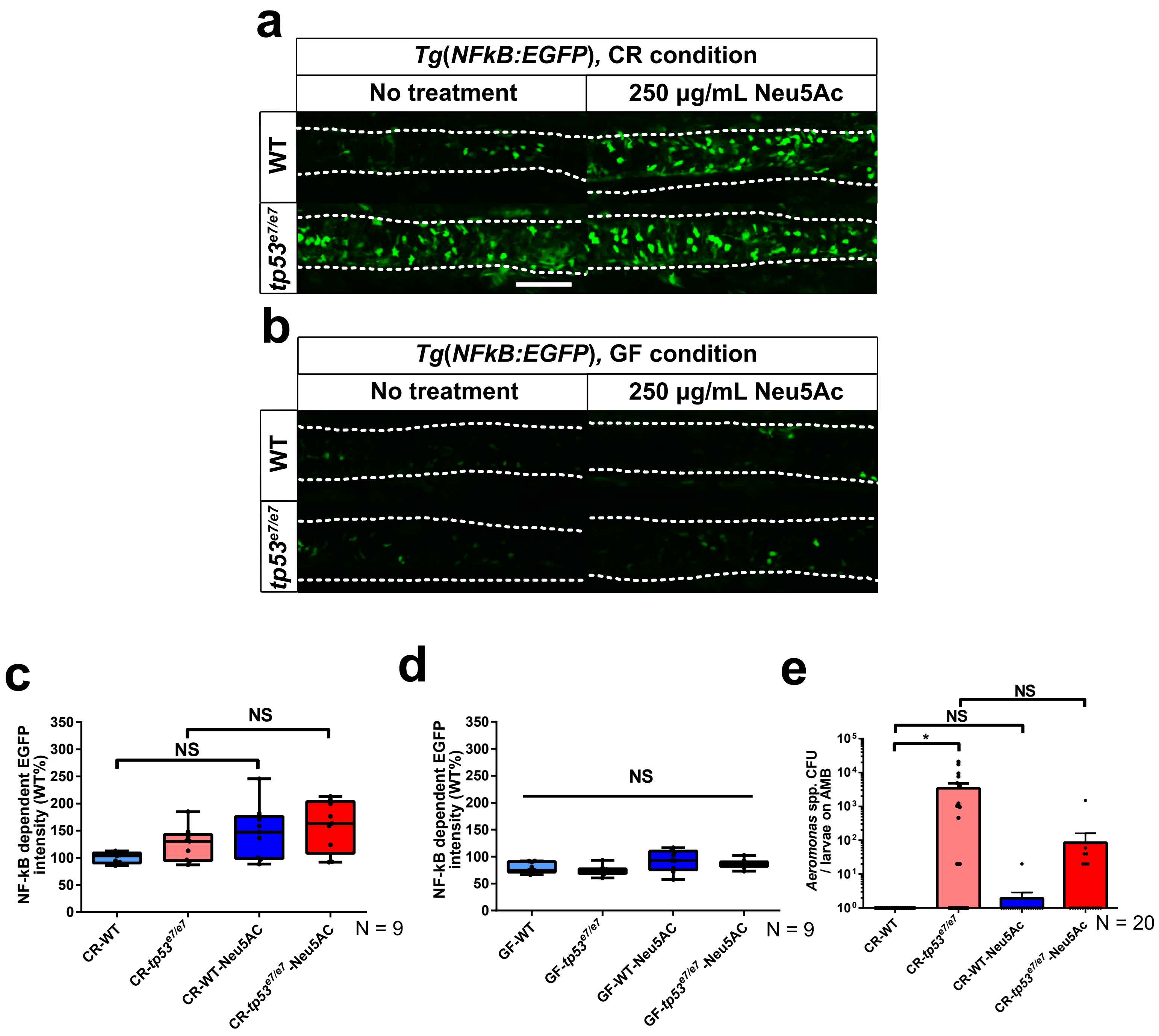
a
Tg(NFkB:EGFP), CR condition
No treatment
250 μg/mL Neu5Ac
WT
tp53e7/e7
b
Tg(NFkB:EGFP), GF condition
No treatment
250 μg/mL Neu5Ac
WT
tp53e7/e7
c
NF-kB dependent EGFP intensity (WT%)
NS
NS
CR-WT
CR-tp53e7/e7
CR-WT-Neu5AC
CR-tp53e7/e7 -Neu5AC
N = 9
d
NF-kB dependent EGFP intensity (WT%)
NS
GF-WT
GF-tp53e7/e7
GF-WT-Neu5AC
GF-tp53e7/e7 -Neu5AC
N = 9
e
Aeromonas spp. CFU / larvae on AMB
*
NS
NS
CR-WT
CR-tp53e7/e7
CR-WT-Neu5AC
CR-tp53e7/e7 -Neu5Ac
N = 20

Figure S16.

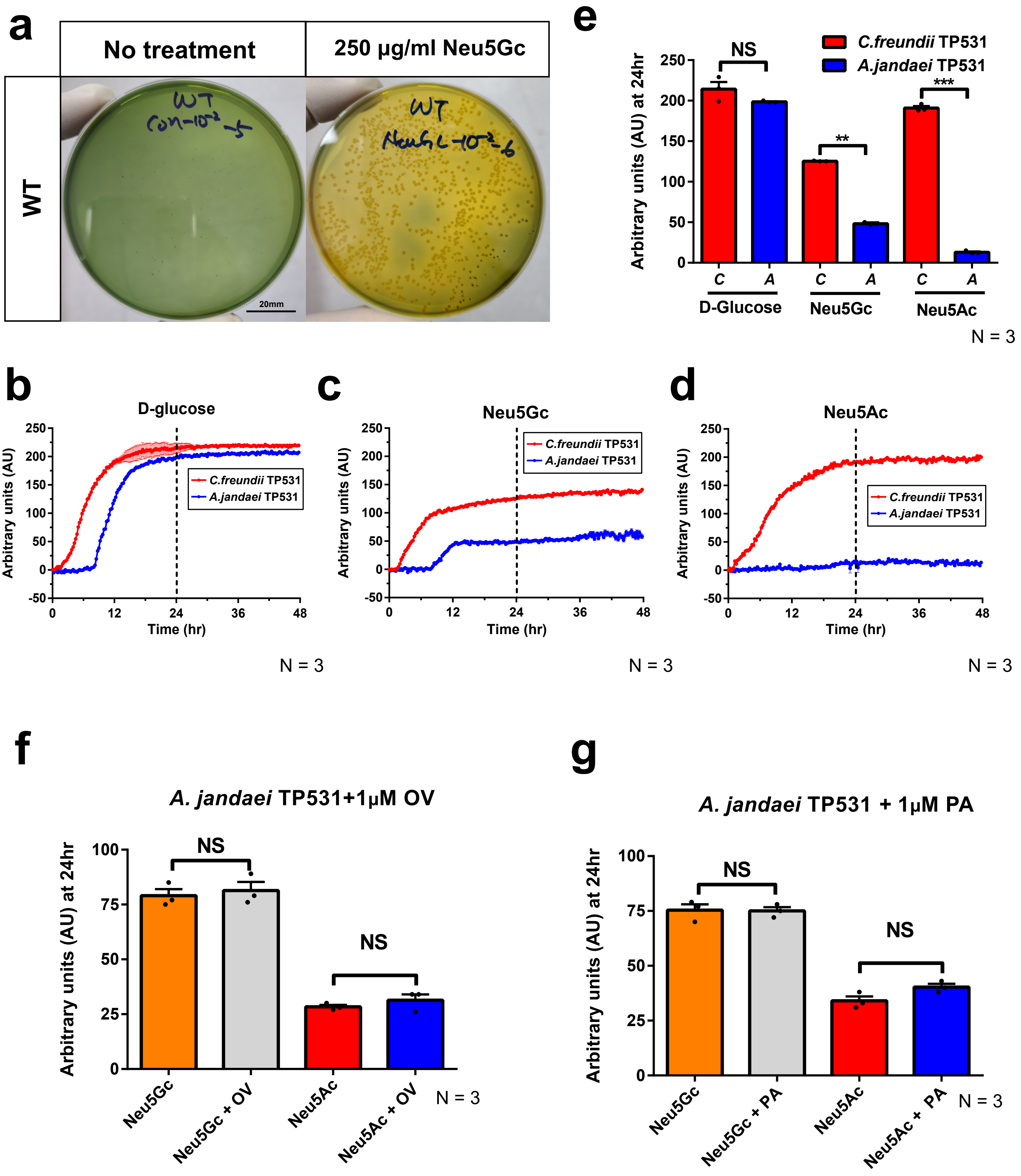
a
No treatment
250 μg/ml Neu5Gc
WT
20mm
b
D-glucose
Arbitrary units (AU)
Time (hr)
C.freundii TP531
A.jandaei TP531
N = 3
c
Neu5Gc
Arbitrary units (AU)
Time (hr)
C.freundii TP531
A.jandaei TP531
N = 3
d
Neu5Ac
Arbitrary units (AU)
Time (hr)
C.freundii TP531
A.jandaei TP531
N = 3
e
C.freundii TP531
A.jandaei TP531
Arbitrary units (AU) at 24hr
NS
**
***
C A C A C A
D-Glucose
Neu5Gc
Neu5Ac
N = 3
f
A. jandaei TP531+1μM OV
Arbitrary units (AU) at 24hr
NS
NS
Neu5Gc
Neu5Gc + OV
Neu5Ac
Neu5Ac + OV
N = 3
g
A. jandaei TP531 + 1μM PA
Arbitrary units (AU) at 24hr
NS
NS
Neu5Gc
Neu5Gc + PA
Neu5Ac
Neu5Ac + PA
N = 3

Figure S17.

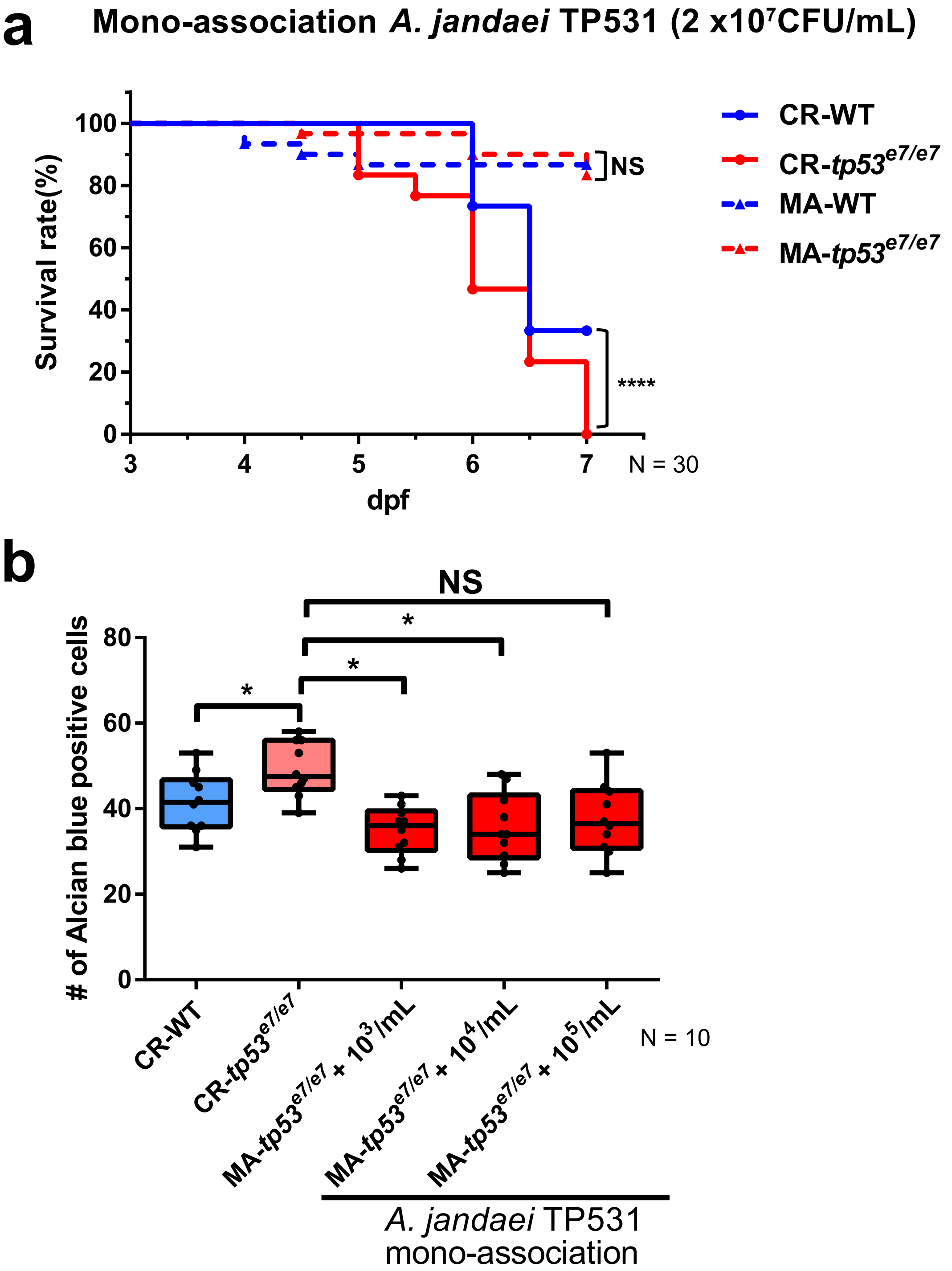
a
Mono-association A. jandaei TP531 (2 x10⁷CFU/mL)
Survival rate(%)
100
80
60
40
20
0
3
4
5
6
7
dpf
N = 30
NS
****
CR-WT
CR-tp53e7/e7
MA-WT
MA-tp53e7/e7
b
# of Alcian blue positive cells
80
60
40
20
0
NS
*
*
*
CR-WT
CR-tp53e7/e7
MA-tp53e7/e7 + 10³/mL
MA-tp53e7/e7 + 10⁴/mL
MA-tp53e7/e7 + 10⁵/mL
N = 10
A. jandaei TP531
mono-association

Figure S18.

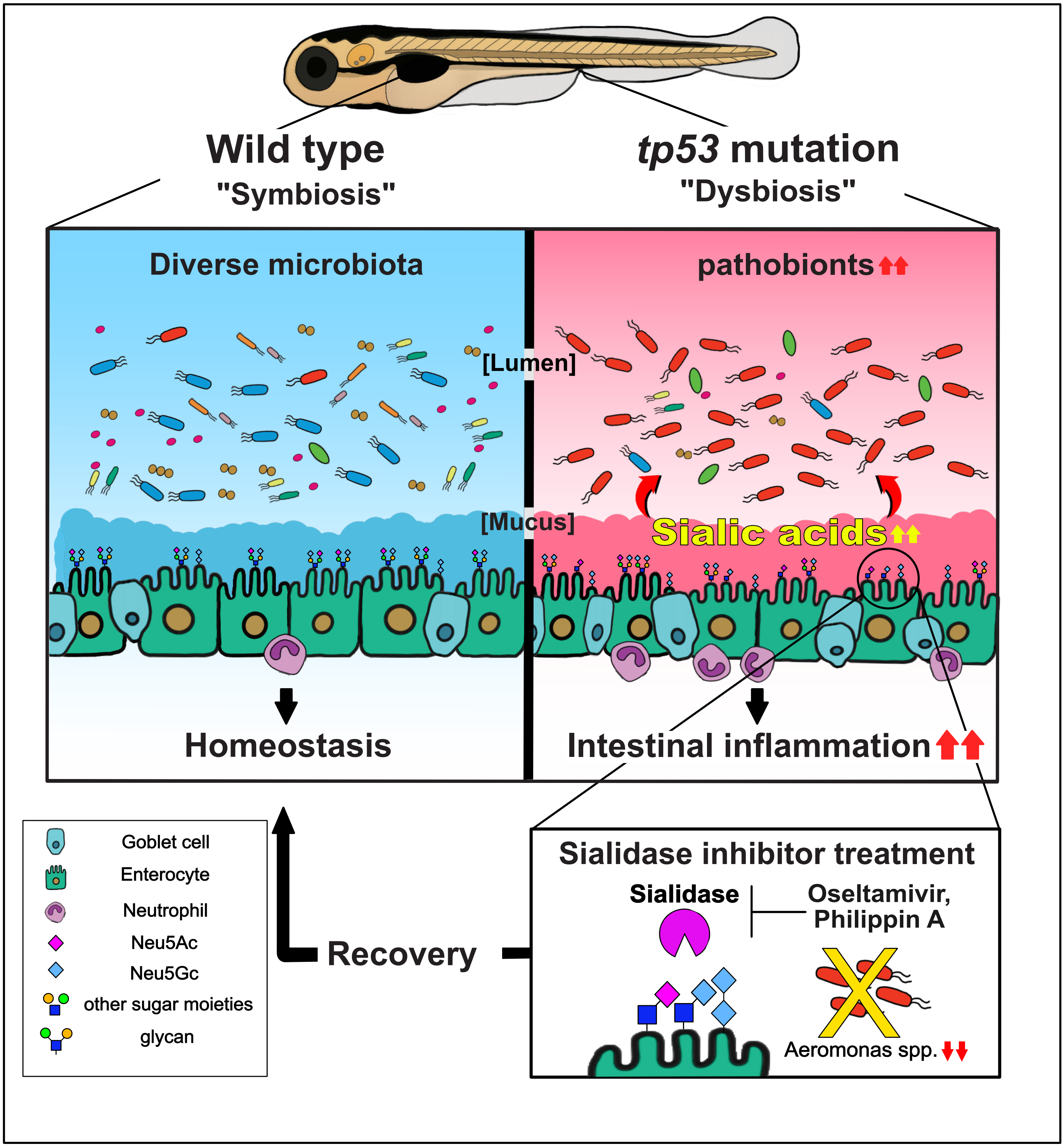

Wild type
"Symbiosis"
tp53 mutation
"Dysbiosis"
Diverse microbiota
pathobionts
[Lumen]
[Mucus]
Sialic acids
Homeostasis
Intestinal inflammation
Goblet cell
Enterocyte
Neutrophil
Neu5Ac
Neu5Gc
other sugar moieties
glycan
Recovery
Sialidase inhibitor treatment
Sialidase
Oseltamivir,
Philippin A
Aeromonas spp.
