# Supplementary material for: Host tp53 mutation induces gut dysbiosis eliciting inflammation through disturbed sialic acid metabolism
Source: Microbiome. 2022 Jan 6;10:3. doi: 10.1186/s40168-021-01191-x (PMC8733924; doi:10.1186/s40168-021-01191-x)
Supplement: Supplementary file 2 — Additional file 1: Supplementary methods and Supplementary Figures. Figure S1. tp53 mutant GITs are aberrantly infiltrated by increased numbers of neutrophils and exhibit hyperimmune responses similar to those induced by DSS treatment. Figure S2. Overview of the experimental procedure and comparisons of gross anatomy between the wild type and tp 53 mutants. Figure S3. The increased number of Alcian blue-positive goblet cells in tp53 mutants are due to Gram (-) bacteria. Figure S4. Gamma-proteobacteria class is enriched in the GITs of tp53 mutants. Figure S5. Aeromonas spp., Citrobacter spp., and Pseudomonas spp. are enriched in the tp53 mutant GITs. Figure S6. E. coli does not induce the increase of Alcian blue-positive goblet cells. Figure S7. Photobacterium damselae DreWT1 isolated from GITs of WT does not induce the increase of NFκB-EGFP activity. Figure S8. Endogenous Aeromonas spp. as well as Pseudomonas spp. and Citrobacter spp. were isolated from tp53 mutants in AMB agar plate culture. Figure S9. tp53 mutation alters metabolic pathways in GITs. Figure S10. SCFA levels show no differences between WT and tp53 mutants. Figure S11. Oseltamivir treatment does not alter free sialic acid levels of the host. Figure S12. Neu5Gc, but not Neu5Ac is utilized as a carbon source by Aeromonas jandaei TP531 for its growth. Figure S13. Elevated inflammation elicited by exogenous addition of mCherry-tagged A. jandaei TP531 in tp53 mutants is abolished by limiting available sialic acids with oseltamivir. Figure S14. Neu5Gc supplementation elevates intestinal inflammation but does barely promote Aeromonas blooming. Figure S15. Neu5Ac supplementation elevates intestinal inflammation in a microbiota-dependent manner, but does not promote blooming of Aeromonas spp.. Figure S16. Citrobacter spp. may outcompete Aeronomas spp. when Neu5Gc and Neu5Ac are supplemented as carbon sources. Figure S17. Monoassociation with A. jandaei TP531 does not induce the lethality and the increase of A [file 40168_2021_1191_MOESM2_ESM.zip › 4. Additional file 1_final ver.docx]

**Additional file 1:**

**Supplementary information**

**Supplementary methods**

**Microinjection and Generation of transgenic zebrafish lines**

To generate NFκB activity reporter and neutrophil-specific transgenic zebrafish, *pNF*κ*B:EGFP* was a gift from John Rawls (Addgene plasmid # 44922; http://n2t.net/addgene:44922; RRID:Addgene_44922) and *ptol2-mpx:mCherry* was a gift from Anna Huttenlocher (Addgene plasmid # 29585; http://n2t.net/addgene:29585; RRID:Addgene_29585). Plasmid preparations were performed by using plasmid midi kit (Qiagen) following the manufacturer’s protocol. Plasmid DNA concentration was measured using a NanoDrop 2000 Spectrophotometer (Thermo). To generate transgenic lines, zebrafish embryos were injected with approximately 1~2 nL of a DNA/RNA solution containing 25 ng/μL Tol2 transposase mRNA and 20 ng/μL Tol2-based transgenic construct into one-cell staged WT eggs [1]. The embryos showing targeted tissue specific fluorescence expression were screened and raised to adulthood and the F0 adult fish were crossed to WT fish for analyzing germline transmission by screening the offspring under the SZX16 microscope. Stable transgenic zebrafish were crossed to WT or *tp53 ^e7/e7^* mutant zebrafish.

**Histological sectioning and H&E staining**

For histology, WT and *tp53* mutant at 7dpf were fixed in 4 % paraformaldehyde (PFA) for overnight at 4^o^C. Following dehydration through increasing alcohol concentrations and xylene washes, specimens of larvae were embedded in paraffin and sagittal sectioned at 7 µm intervals

After deparaffinization and hydration, the slides were stained with hematoxylin & eosin (H&E) staining solution (Merck). The sections were sealed with neutral mounting medium and were observed using Eclipse Ci light microscope (Nikon).

**Confocal microscopic analysis for intestinal neutrophils**

To analyze intestinal neutrophils using *Tg(mpx:mCherry)* with high resolution, *Tg(mpx:mCherry)* of WT and *tp53^e7/e7^* mutant larvae at 7 dpf in CR condition were fixed with 1X staining solution for overnight at 4^o^C. Fixed larvae were washed briefly with 1X PBST and embedded on the glass-bottomed imaging dishes with 1 % low melting point agarose. The mid-distal intestines spanning the region of 250 μm from the anus were imaged using FV1000 confocal microscope (Olympus) with the identical fluorescence laser condition. Confocal z-projections were made by stacking 10~11 sections with 5 μm thickness. The numbers of *mCherry*-positive neutrophils in the mid-distal intestines were counted using the z-projected confocal images.

**Isolation of *Photobacterium damselae* from WT GITs**

In order to isolate commensal bacteria in WT gastrointestinal tracts (GITs), 20 WT zebrafish larvae GITs at 7 dpf under CR condition were dissected using fine pins and forceps and pooled in sterile E3 egg water. After homogenized by pestles, GITs in suspension were serially diluted and plated on 1/10 dilution Trypticase soy agar (TSA, DB) under aerobic conditions at 37 °C. Single colonies with different morphologies were picked and further analyzed by 16S rRNA sequencing. *Photobacterium damselae* from WT GITs, designated as *P. damselae* DreWT1 strain, was successfully cultivated under aerobic culture condition and further characterized for its ability to induce immune responses upon association with the host.

**Treatment of antibiotics**

In order to narrow down specific groups of microbes in the microbiota that regulate intestinal inflammation, WT and *tp53* mutant larvae at 3 dpf in CR condition were treated with 1 μg/mL polymyxin B (Sigma-Aldrich) and 10 μg/mL vancomycin (Sigma-Aldrich) for 4 days (Additional file 1: Figure S2a). Polymyxin B and vancomycin were used to kill gram-negative and gram-positive bacteria, respectively. To confirm the perturbation of microbiota, intestinal microbiota was isolated using TSA plates. Briefly, antibiotics-treated zebrafish larvae were euthanized with 1X tricaine (MS-222) and washed 3 times with sterile 1X PBST to remove bacteria loosely attached to the skin. They were transferred to tubes containing glass beads and added with 200 μL of autoclaved E3 Egg water. After bead beating to homogenize specimens, serial dilutions of suspension were plated on TSA. Colony forming unit (CFU) was counted after overnight incubation at 37 °C. A bacterial colony from the culture plate was emulsified over glass slide in suspension of 3% KOH using the loop. The suspension was stirred continuously for one minute and then loop was gently pulled up from it. The test was considered positive if string was seen within first 30 seconds after mixing in KOH solution. At least 20 colonies were checked by 3% KOH test. When treated with antibiotics, population of more than 90% of microbiota were changed to Gram negative or positive bacteria, respectively.

**DSS treatment**

To induce dysbiosis of microbiota, we used dextran sulfate sodium salt (DSS, MP Biomedicals),a well-known drug for inducing enterocolitis in various animal models. The treatment of DSS was followed as previously reported [2]. A dose of 0.5 % DSS was known to be the highest concentration that induced inflammation but did not cause significant mortality. WT and *tp53* mutant zebrafish embryos at 3 dpf under CR conditions were immersed with 0.5 % DSS for 4 days.

**Quantitative RT–PCR Analysis**

To prepare total RNA, 25 GITs of WT and *tp53 mutant* zebrafish larvae at 7 dpf under CR and GF condition were dissected using fine pins and forceps manually. Total RNA was isolated using Trizol reagent (Invitrogen) and Direct-zol RNA miniprep kit (Zymo Research). RNA quality was assessed by NanoDrop 2000 spectrophotometer (Thermo). cDNA was synthesized using a SuperScript III First-Strand Synthesis System (Invitrogen). For quantitative RT-PCR analysis, ABI Prism 7900 Sequence Detection System (Applied Biosystems) and SyberGreen PCR Core reagents (Applied Biosystems) were used. The synthesized cDNA was amplified by PCR using: the forward primer 5′- CTGGTTCAAGGGATGGAAGA-3′ and reverse primer 5′- CACACGACCCACAGGTACAG-3′ for *ef1alpha*; the forward primer 5′- GCCGTGCAGATCATCAAAC-3′ and reverse primer 5′- CCGCTGTAGTTAGGGAAGGT-3′ for *ikbaa*. mRNA levels were expressed as the relative fold change against the normalized *ef1alpha* mRNA. The comparative cycle threshold (Ct) method (User Bulletin 2, Applied Biosystems) was used to analyze the data. The data represent duplication results (N = 2) of averaged triplicate RT-PCR experiments.

**Assessment of sialic acids on bacteria growth**

To identify the ability of utilization of sialic acids as carbon sources on isolated *Aeromonas jandaei* TP531 and *Citrobacter freundii* TP531, growth kinetic assays of isolated bacteria strains with sialic acids were performed with a modification of a method of altering the Phenotype Microarray (Biolog) based on the reduction of tetrazolium violet as a reporter of active metabolism. All procedures were performed as indicated by the manufacturer guideline and previous study [3] Briefly, bacterial cell suspensions (~10^5^ CFU/mL) were prepared in a chemically defined broth (CDB, 4 μL /1mL amino acids mix solution (Sigma-Aldrich), 10 mg/mL NaCl (Sigma-Aldrich), 135 μg/mL MgSO_4_•7H_2_O (Sigma-Aldrich), 2 μg/mL FeSO_4_g•7H_2_O (Sigma-Aldrich), 1 mg/mL KH_2_PO_4_ (Sigma-Aldrich), 1 mg/mL K_2_HPO_4_ (Sigma-Aldrich)) with either 10 mM Neu5Ac, Neu5Gc or 10 mM D-glucose (Sigma-Aldrich). D-glucose was used as positive control. 1% tetrazolium violet was added to the suspensions and the mixtures were inoculated (100 μL per well). The colors of inoculated wells changed to purple during bacteria growth due to the reduction of tetrazolium violet into formazan. After inoculation, the plates were incubated for 48 hr at 37 °C and recorded every 15 min in the OmniLog incubator/reader (Biolog) with a charge-coupled device camera. The experiments were repeated three times.

**Assessment of sialidase inhibitors on *A. jandaei* TP531 growth**

To test whether growth of isolated *A. jandaei* TP531 was directly suppressed by the sialidase inhibitors, *A. jandaei* TP531 was cultivated with 1 μM OV or PA followed by the Phenotype Microarray described above. Briefly, *A. jandaei* TP531 (~10^5^ CFU/mL) was incubated in a chemically defined broth with 10 mM sialic acids (either Neu5Gc or Neu5Ac), and 1μM OV or 1μM PA was added for 48 hr at 37 °C. Bacteria growth was measured using tetrazolium violet and OmniLog incubator/reader. Experiments were repeated three times. Directly suppressive effects by OV or PA was evaluated by comparing of *A. jandaei* TP531 growth at 24 hr.

**Quantification of short chain fatty acids by Gas Chromatography/Mass Spectrometry (GC/MS)**

To measure of shot chain fatty acids (SCFAs: acetic acid, propionic acid and butyric acid) in zebrafish larvae at 7 dpf, analysis of SCFAs was modified as previously reported [4]. Sodium acetate (Sigma-Aldrich), sodium propionate (Sigma-Aldrich), and sodium butyrate (Sigma-Aldrich) were used as standard chemicals. 50 pooled whole body of zebrafish larvae were homogenized in 0.1% formic acid spiked with 4-methyl valeric acid using bead beating. The 4-methyl valeric acid (Sigma-Aldrich) as internal standard (a final concentration of 1 μg/mL) was used to correct for injection variability between samples and minor changes in the instrument response. After bead beating to extract SCFAs, the samples were centrifuged at 3500 rpm for 10 min. The supernatants were transferred to glass vials for pentafluorobenzyl (PFB, Sigma-Aldrich) derivatization. An equal volume of the catalyst 0.1 M tetrabutylammonium hydrogensulfate (Sigma-Aldrich) was added and the pH was adjusted to 9 using 0.5 M NaOH. After mixing for about 1 min, an equal volume of 0.5 % PFBBr derivatization reagent dissolved in dichloromethane was added. The mixture was heated at 60°C for 20 min and centrifuged at 3000 rpm for 5 min to separate the organic and aqueous layers. The organic layer was transferred to another tube and evaporated using Scan Vac Scan speed vacuum centrifuge 40 (LaboGene). The dried residue was dissolved in 100μL of ethyl acetate. Analysis of SCFAs were carried out using a 5975C gas chromatograph (Agilent) equipped with an HP-5MS column (30 m X 0.25 mm internal diameter, 0.25 μm width; Agilent). The samples were introduced via split (ratio 5:1) injection with the port heated to 250 °C. The initial helium was used as the carrier gas at a flow rate of 1.0 mL/min. The oven temperature was initially held at 80 °C for 1.0 min, increased to 150 °C with a 10 °C per min rate, and then raised to 300 °C with a 20°C per min rate, where it was held for 5 min. The mass spectrometer interface temperature was set to 260 °C. After GC separation, concentrations of SCFAs were quantified by area of MS intensity of samples/area of MS intensity of internal standard, plotted against standard curves of corresponding authentic standards.

**Supplementary data titles and legends**

**Figure S1.** ***tp53* mutant GITs are aberrantly infiltrated by increased numbers of neutrophils and exhibit hyperimmune responses similar to those induced by DSS treatment.** (a) Representative images of *Tg(mpx:mCherry)* visualizing neutrophils in the mid-distal intestine of WT or *tp53* mutant at 7 dpf under CR conditions. White dashed lines denote boundaries of GITs based on DIC bright field images. Scale bar = 50 μm. (b) A boxed plot showing comparison of the number of *mCherry*-positive neutrophils in the mid-distal intestines of WT and *tp53* mutants under CR conditions. The number of *mCherry*-positive neutrophils was significantly increased in *tp53* mutant GITs. N = 13 each. (c) Representative images showing that treatment of 0.5 % dextran sulfate sodium (DSS) elevated intestinal inflammatory response of WT and *tp53* mutants monitored by *Tg(NFκB:EGFP)*. White dashed lines denote boundaries of GITs based on DIC bright field images. Scale bar = 50 μm. (d) A boxed plot showing comparison of *NFκB* dependent fluorescence intensities in the mid-distal intestines of WT and *tp53* mutants with or without 0.5% DSS. (e) Alcian blue staining visualizing of goblet cells in the mid-distal intestines of WT or *tp53* mutants with or without 0.5 % DSS. Scale bar = 50μm. (f) A boxed plot showing comparison of Alcian blue-positive goblet cell numbers in the mid-distal intestines of WT and *tp53* mutants with or without 0.5% DSS. Both *NFκB* dependent EGFP intensities and Alcian blue-positive goblet cell numbers were increased with 0.5% DSS treatment. N = 10 each. The boxed plots were statistically estimated non-parametric Friedman test followed by Dunn's multiple comparisons test. Data are represented as mean ± SEM. * < *p* = 0.05; ** < *p* = 0.01.

**Figure S2. Overview of the experimental procedure and comparisons of gross anatomy between the wild type and *tp53* mutants.** (a) Wild type and *tp53* mutant natural bred embryos were incubated under conventionally raised (CR) or germfree (GF) conditions at 28.5℃. After chemicals or bacteria were treated at 3 dpf, sample collections and detailed analyses using *Tg(NFκB:EGFP)*-based EGFP intensity and Alcian blue-stained goblet cell number counting were conducted at 7 dpf. (b) Representative Bright-field (DIC) images of whole body of WT and *tp53* mutant at 7dpf. There was no generally morphological difference between WT and *tp53* mutant. (c) Representative sagittal sections by H&E(hematoxylin-eosin) staining showed intestinal morphologies in WT and *tp53* mutant. There was no significant morphological difference in intestinal bulb. But goblet cells were increased in *tp53* mutant mid-distal intestine.

**Figure S3.** **Increased number of Alcian blue-positive goblet cells in *tp53* mutants are due to Gram (-) bacteria.** (a) A schematic of antibiotics experiments in which WT or *tp53* mutant embryos under CR conditions were incubated with polymyxin B (1 μg/mL) or vancomycin (10 μg/mL) since 3 dpf for 4days. (b, c) Boxed plots showing Alcian blue-positive goblet cell numbers in the mid-distal intestine of WT (b) and *tp53* mutants (c) with antibiotics treatment at 7dpf. The number of Alcian blue-positive goblet cells was significantly decreased in WT and *tp53* mutant intestines only with polymyxin B, similar to that of GF conditions. N = 10 each. The boxed plots were statistically estimated non-parametric Friedman test followed by Dunn's multiple comparisons test. Data are represented as mean ± SEM. * < *p* = 0.05; ** < *p* = 0.01; *** < *p* = 0.005. NS, not significant.

**Figure S4. Gamma-proteobacteria class is enriched in the GITs of *tp53* mutants.** (a) Metagenome analysis of the intestinal microbiota of WT and *tp53* mutants with or without DSS treatment at a class level. Each class is color-coded according to the key panel on the right. Each group was repeated three times. (b-d) Bar graphs showing the log-ratios of classes of bacilli (b), actinobacteria (c), and gamma-proteobacteria (d) relative to alpha-proteobacteria in the GITs of the WT and *tp53* mutants at 7 dpf with or without DSS treatment by pyrosequencing of 16s rRNA gene. Alpha-proteobacteria class was used as reference frame since this taxon is consistently abundant across experimental conditions. Gamma-proteobacteria class was highly enriched in *tp53* mutants and DSS-treated group. Bar graphs were statistically estimated one-way ANOVA followed by Newman-Keuls multiple comparisons test. Data are represented as mean ± SEM. *** < *p* = 0.005. NS, not significant.

**Figure S5.** ***Aeromonas* spp., *Citrobacter* spp., and *Pseudomonas* spp. are enriched in the *tp53* mutant GITs.** (a) A table showing OTU analysis of bacteria increased in *tp53* mutants with or without DSS treatment, based on pyrosequencing of 16s rRNA gene. (b-d) Bar graphs showing the log-ratios of enriched genera of *Aeromonas* (b), *Citrobacter* (c) and *Pseudomonas* spp. (d) relative to *Acinetobacter* spp. in the GITs of *tp53* mutants at 7 dpf with or without DSS treatment by OUT analysis. Genus of *Acinetobacter* spp. was used as reference frame since this taxon is consistently abundant across experimental conditions. N = 3 each. Bar graphs were statistically estimated one-way ANOVA followed by Newman-Keuls multiple comparisons test. Data are represented as mean ± SEM. ** < *p* = 0.01. NS, not significant.

**Figure S6.** ***E. coli* does not induce the increase of Alcian blue-positive goblet cells.** (a) Lethality curve of *Escherichia coli* DH10B association (1 x10^8^ CFU/mL) with WT or *tp53* mutants starting at 3 dpf for 4 days. N = 60 each. Lethality curve was statistically estimated by Gehan-Breslow-Wilcoxon test. (b, c) Boxed plots of Alcian blue-positive goblet cell numbers in the mid-distal intestines of WT (b) and *tp53* mutants (c) associated with increasing concentrations of *E. coli* (10^3^~10^5^ CFU/mL) under CR conditions. N = 10 each. The boxed plots were statistically estimated non-parametric Friedman test followed by Dunn's multiple comparisons test. Data are represented as mean ± SEM. NS, not significant.

**Figure S7.** ***Photobacterium damselae* DreWT1 isolated from GITs of WT does not induce the increase of *NFκB-EGFP* activity.** (a) A lethality curve of *Photobacterium damselae* DreWT1 isolated from GITs of WT association (1 x10^8^ CFU/mL) with WT or *tp53* mutants host starting at 3 dpf for 4 days. N = 60 each. Lethality curve was statistically estimated by Gehan-Breslow-Wilcoxon test. (b) Representative images showing that association of *P. damselae* DreWT1 (10^5^ CFU/mL) did not elevate intestinal inflammatory response of WT and *tp53* mutants under CR conditions monitored by *Tg(NFκB:EGFP)*. (c) A boxed plot showing comparison of *NFκB* dependent EGFP intensities in the mid-distal intestines of WT and *tp53* mutants with *P. damselae* DreWT1*.* N = 10 each. (d) Representative images showing that association of *A. jandaei* TP531 (10^4^ CFU/mL) elevated intestinal inflammatory response of WT and *tp53* mutants under CR conditions monitored by *Tg(NFκB:EGFP)*. White dashed lines denote boundaries of GITs based on DIC bright field images. Scale bar = 50 μm. (e) A boxed plot showing comparison of *NFκB* dependent EGFP intensities in the mid-distal intestines of WT and *tp53* mutants with *A. jandaei* TP531. N = 13 each. The boxed plot was statistically estimated non-parametric Friedman test followed by Dunn's multiple comparisons test. Data are represented as mean ± SEM. * < *p* = 0.05; ** < *p* = 0.01. NS, not significant.

**Figure S8. Endogenous *Aeromonas* spp. *Pseudomonas* spp. and *Citrobacter* spp. were isolated from *tp53* mutants in AMB agar plate culture. (**a) A representative image of bacteria colonies in an AMB plate where at least three types of colonies with different morphology were cultivated. Scale bar = 5 mm. (b) Agarose gel electrophoresis of PCR amplicons after pan-bacteria- or A*eromonas*-specific amplification using each bacterial genomic DNA which were extracted from cultured bacteria with different morphologies and used as templates for PCR using pan-bacteria- or *Aeromonas-* specific primer pairs. Amplicons of pan-bacteria (27F/1492R) were used to identify isolated bacteria by Sanger sequencing, which turned out to be *Aeromonas veronii*, *Pseudomonas otitidis* and *Citrobacter baraakii*, respectively.

**Figure S9.** ***tp53* mutation alters metabolic pathways in GITs.** (a) A schematic depicting *tp*53 genomic loci based on mRNA sequencing of WT and *tp53* mutant GITs under CR or GF conditions. The point mutation of *tp53* mutants (ATG to AAG conversion, Met to Lys) was confirmed in the *tp53* gene of both conditions. Numbers in small letter denote read depth from RNA-seq data. (b) Parametric analysis of gene set enrichment (PAGE) of gene ontology (GO) analysis related to NFκB pathway of transcriptomes of CR-WT *vs*. CR-*tp53* mutants or GF-WT *vs*. GF-*tp53* mutants, respectively. (c) Quantitative RT-PCR of the *NFκB* target gene *ikbaa* in GITs of WT and *tp53* mutants under CR or GF conditions. N = 2 each. (d, e) Parametric analysis of gene set enrichment (PAGE) of KEGG pathway related to carbohydrate metabolism (d) or lipid metabolism (e) of transcriptomes of CR-WT *vs*. CR-*tp53* mutants or GF-WT *vs*. GF-*tp53* mutants, respectively. Positive number of enrichment scores (Y axis) denotes the increase of the pathway in *tp53* mutants.

**Figure S10.** **SCFA levels show no differences between WT and *tp53* mutants.** (a-c) Bar graphs of quantification of SCFAs including acetic acid (a), propionic acid (b), and butyric acid (c) by Gas Chromatography/Mass Spectrometry (GC/MS) of WT and *tp53* mutants at 7dpf under CR conditions. The 4-methyl valeric acid was used as internal standard (a final concentration of 1 μg/mL) to correct variability among samples and minor changes of the instrument response. N = 4 each. Bar graphs were statistically estimated one-way ANOVA followed by Newman-Keuls multiple comparisons test. NS, not significant.

**Figure S11.** **Oseltamivir treatment does not alter free sialic acid levels of the host.** (a) HPLC-FLD chromatograms of DMB labeled sialic acids from the GITs of WT. The top chromatogram is the mixed sialic acids standard. The bottom chromatogram is the specimen of zebrafish WT. Major sialic acids in the GITs of zebrafish were Neu5Gc and Neu5Ac. (b) Flowchart of the experimental procedure to analyze free or bounded sialic acids. Total SAs is the sum of the free and bounded SAs. (c, d) Bar graphs showing comparison of free Neu5Gc (c) and Neu5Ac (d) level in whole body of WT and *tp53* mutants with or without 1 μM oseltamivir treatment. N = 3 each. (e, f) Bar graphs showing comparison of total Neu5Gc (e) and Neu5Ac (f) level in whole body of WT and *tp53* mutants with or without 1 μM oseltamivir treatment. N = 3 each. Bar graphs were statistically estimated one-way ANOVA followed by Newman-Keuls multiple comparisons test. Data are represented as mean ± SEM. * < *p* = 0.05; ** < *p* = 0.01; NS, not significant.

**Figure S12. Neu5Gc, but not Neu5Ac, is utilized as a carbon source by *Aeromonas jandaei* TP531 for its growth.** (a) Growth curve of *Aeromonas jandaei* TP531 with treatments of 10 mM D-glucose, Neu5Gc and Neu5Ac for 48hr at 37℃. D-glucose was treated as a positive control. Black dashed line denotes time point for analysis of statistics. (b) A bar graph showing the relative growths of *A. jandaei* TP531 with treatments of Neu5Gc, Neu5Ac and glucose at 24hr. N = 3 each. Bar graph was statistically estimated one-way ANOVA followed by Newman-Keuls multiple comparisons test. Data are represented as mean ± SEM. *** < *p* = 0.005; NS, not significant.

**Figure S13. Elevated inflammation by exogenous *mCherry*-tagged *A. jandaei* TP531** **in *tp53* mutants is abolished by limiting available sialic acids with oseltamivir.** (c)Representative images of elevated inflammation and colonization by exogenously added *mCherry*-tagged *A. jandaei* TP531 in WT and *tp53* mutants with or without 1 μM oseltamivir treatment. White dashed lines denote boundaries of GITs based on DIC bright field images. Scale bar = 50 μm. (d) A boxed plot showing that elevated inflammation in the mid-distal intestines of *tp53* mutants by *mCherry*-tagged *A. jandaei* TP531 was abolished upon oseltamivir treatment. (e) A bar graph showing that the enhanced survivability of *mCherry*-tagged *A. jandaei* TP531 in *tp53* mutants was abolished upon oseltamivir treatment. (f) A bar graph showing that oseltamivir treatment did not significantly affect total bacterial populations on TSA plates. N =15 each. The boxed plot and bar graph were statistically estimated by non-parametric Friedman test followed by Dunn's multiple comparisons test. Data are represented as mean ± SEM. * < *p* = 0.05; ** < *p* = 0.01; NS, not significant.

**Figure S14.** **Neu5Gc supplementation elevates intestinal inflammation but does not promote *Aeromonas* blooming.** (a) Representative fluorescence images of the mid-distal intestines of *Tg(NFκB:EGFP)* showing that treatment of 250 μg/mL Neu5Gc (N-Glycolyl-neuraminic acid) elevated NFκB activity under CR conditions. White dashed lines denote boundaries of GITs based on DIC bright field images. Scale bar = 50 μm. (b) A boxed plot showing comparison of the fluorescence intensities of the mid-distal intestines of WT and *tp53* mutants with or without 250 μg/mL Neu5Gc under CR conditions. N = 12 each. (c) A bar graph showing comparison of the *Aeromonas* spp. colony forming unit (CFU) of WT and *tp53* mutants with or without 250 μg/mL Neu5Gc in AMB plates under CR conditions. The number of *Aeromonas* spp. CFU of *tp53* mutant was increased compared with that of WT, while the Neu5Gc treatment of WT or *tp53* mutants did not affect the *Aeromonas* spp. CFU in AMB plates. N = 20 each. The boxed plot and bar graph were statistically estimated by non-parametric Friedman test followed by Dunn's multiple comparisons test. Data are represented as mean ± SEM. * < *p* = 0.05; ** < *p* = 0.01. NS, not significant.

**Figure S15.** **Neu5Ac supplementation elevates intestinal inflammation in a microbiota-dependent manner, but does not promote blooming of *Aeromonas* spp..** (a, b) Representative fluorescence images of the mid-distal intestines of *Tg(NFκB:EGFP)* showing that treatment of 250 μg/mL Neu5Ac (N-acetyl neuraminic acid) elevated NFκB activity under CR conditions (a), which was abrogated under GF conditions (b). White dashed lines denote boundaries of GITs based on DIC bright field images. Scale bar = 50 μm. (c, d) Boxed plots showing comparison of the fluorescence intensities of the mid-distal intestines of WT and *tp53* mutants with or without 250 μg/mL Neu5Ac under CR (c) or GF conditions (d). N = 9 each. (e) A bar graph showing comparison of the *Aeromonas* spp. colony forming unit (CFU) of WT and *tp53* mutants with or without 250 μg/mL Neu5Ac in AMB plates under CR conditions. Dramatically increased number of *Aeromonas* spp. colony in *tp53* mutants was abrogated upon 250 μg/mL Neu5Ac treatment, unexpectedly. N = 20 each. The boxed plot and bar graph were statistically estimated by non-parametric Friedman test followed by Dunn's multiple comparisons test. Data are represented as mean ± SEM. * < *p* = 0.05; ** < *p* = 0.01. NS, not significant.

**Figure S16. *Citrobacter* spp. may outcompete *Aeronomas* spp. when Neu5Gc and Neu5Ac are supplemented as carbon sources.** (a) Representative pictures of bacterial cultures from WT specimens with 250 μg/mL Neu5Gc treatment using *Aeromonas* medium base (AMB). Scale bar = 20 mm. Yellow colonies boomed at the Neu5Gc treatment condition were identified *Citrobacter* spp. (b, c, d) Growth curves showing comparison of between *Aeromonas jandaei* TP531 and *Citrobacter freundii* TP531 with treatments of 10 mM D-glucose (b), Neu5Gc (c) and Neu5Ac (d) for 48hr at 37℃. D-glucose was treated as a positive control. Black dashed line denotes time point for analysis of statistics. To compare growth patterns of *C. freundii* TP531, data of *A. jandaei* TP531 with treatment of carbon source were quoted from the Figure S12. (e) A bar graph showing the relative growths comparisons of between *A. jandaei* TP531and *C. freundii* TP531 with treatments of D-glucose, Neu5Gc and Neu5Ac at 24 hr. *C. freundii* TP531 is able to utilize free sialic acids as carbon source better than *A. jandaei* TP531*.* (f, g) Bar graphs showing the growth *A. jandaei* TP531 with addition of 1 μM OV (f) or PA (g) in the presence of Neu5Gc or Neu5Ac at 24 hr. N = 3 each. Bar graphs were statistically estimated one-way ANOVA followed by Newman-Keuls multiple comparisons test. Data are represented as mean ± SEM. ** < *p* = 0.01; *** < *p* = 0.005; NS, not significant.

**Figure S17. Monoassociation with *A. jandaei* TP531** **does not induce the lethality and the increase of Alcian blue-positive goblet cells.** (a) A lethality curve of *Aeromonas jandaei* TP531 association (2 x10^7^ CFU/mL) with WT or *tp53* mutants starting at 3 dpf for 4 days. N = 30 each. The lethality curve was statistically estimated by Gehan-Breslow-Wilcoxon test. (b) A boxed plot of Alcian blue-positive goblet cell numbers in the mid-distal intestines of *tp53* mutants associated with increasing concentrations of *A. jandaei* TP531 (10^3^~10^5^ CFU/mL) under CR conditions. N = 10 each. The boxed plot was statistically estimated non-parametric Friedman test followed by Dunn's multiple comparisons test. Data are represented as mean ± SEM. * < *p* = 0.05; **** < *p* = 0.001; NS, not significant.

**Figure S18. A proposed working model illustrates dysbiosis and intestinal inflammation by *tp53* mutation via imbalanced sialometabolism and a potential therapeutic intervention.** Zebrafish *tp53* mutant larvae exhibit elevated intestinal inflammation due to dysbiotic gut microbiota with reduced diversity, in contrast to wild type larvae harboring symbiotic microbiota. Enriched pathobionts (e.g. *Aeromonas* spp.) are responsible for enhanced inflammation, with aggressive colonization capability in *tp53* deficiency intestines. *tp53* mutation promotes dysbiosis by supplying excessive intestinal sialic acids and supporting a bloom of sialic acid-exploiting pathobionts. The elevated inflammatory responses due to dysbiosis are efficiently abrogated upon treatment with a specific sialidase inhibitor, oseltamivir and philippin A, that decreases the availability of free sialic acids and prevents the overgrowth of sialic acids-utilizing pathobionts such as *Aeromonas* spp. Therefore, normalizing the imbalance of sialic acid metabolism using a sialidase inhibitor may be a useful therapeutic strategy for *tp53* mutation-driven intestinal dysbiosis and inflammation.

**Supplementary references**

1. Kwan KM, Fujimoto E, Grabher C, Mangum BD, Hardy ME, Campbell DS, Parant JM, Yost HJ, Kanki JP, Chien CB: **The Tol2kit: a multisite gateway-based construction kit for Tol2 transposon transgenesis constructs.** *Dev Dyn* 2007, **236:**3088-3099.

2. Oehlers SH, Flores MV, Hall CJ, Crosier KE, Crosier PS: **Retinoic acid suppresses intestinal mucus production and exacerbates experimental enterocolitis.** *Dis Model Mech* 2012, **5:**457-467.

3. Yi HS, Ahn YR, Song GC, Ghim SY, Lee S, Lee G, Ryu CM: **Impact of a Bacterial Volatile 2,3-Butanediol on Bacillus subtilis Rhizosphere Robustness.** *Front Microbiol* 2016, **7:**993.

4. Park NH, Kim M-S, Lee W, Lee ME, Hong J: **An in situ extraction and derivatization method for rapid analysis of short-chain fatty acids in rat fecal samples by gas chromatography tandem mass spectrometry.** *Analytical Methods* 2017, **9:**2351-2356.
